# Defect-Anchored Dipole Molecules Induce Surface Polarization Facilitating High-Performance Inverted Perovskite Solar Cells

**DOI:** 10.1007/s40820-026-02150-7

**Published:** 2026-03-18

**Authors:** Weichun Pan, Jihuai Wu, Jiexi Pan, Shanyue Wei, Lina Tan, Wenjing Li, Deng Wang, Xuping Liu, Yiming Xie, Jianming Lin, Zhang Lan

**Affiliations:** 1 Engineering Research Center of Environment-Friendly Functional Materials, Ministry of Education, Fujian Key Laboratory of Photoelectric Functional Materials, College of Materials Science and Engineering, Huaqiao University, Xiamen, 361021 People’s Republic of China; 2 Key Laboratory of Environmentally Friendly Functional Materials and Devices, Lingnan Normal University, Zhanjiang, 524048 People’s Republic of China

**Keywords:** Perovskite solar cells (PSCs), 4-aminocyclohexanone hydrochloride (ACHCl), Dipole effect, Surface polarization, Interfacial losses

## Abstract

The improvement in efficiency and stability of inverted perovskite solar cells (PSCs) is primarily constrained by the charge-carrier and energy losses at the interface of perovskite active layer/charge-carrier transport layers. Herein, a kind of dipolar molecule, 4-aminocyclohexanone hydrochloride (ACHCl), is introduced to the surface of perovskite films in PSCs with p-i-n structure. This surface modification ingeniously utilizes the surface defects of perovskite films to anchor the dipolar molecule, thus inducing surface polarization, which not only effectively reduces interfacial defects but also optimizes the energy-level alignment between the interfaces. Specifically, the carbonyl group and chloride ion on ACHCl anchors on the uncoordinated lead ion defects and fills in the halide vacancies on perovskite surface, respectively, which effectively alleviates the trap-state density, thereby reducing the carrier losses caused by defect-assisted recombination at the interface of perovskite layer/hole transport layer. Meanwhile, the anchoring effect of ACHCl facilitates the formation of a relatively ordered cation-dipole layer and induces surface polarization, resulting in more favorable energy-level alignment and enhanced charge-carrier extraction, ultimately reducing interfacial energy losses. Consequently, the effective reduction in interfacial losses facilitates the ACHCl-modified devices to achieve a power conversion efficiency of 26.12% and improved stability.

**Supplementary Information:**

The online version contains supplementary material available at 10.1007/s40820-026-02150-7.

## Highlights

- The carbonyl group and chloride ion of 4-aminocyclohexanone hydrochloride (ACHCl) can synergistically passivate surface defects in the perovskite, thereby mitigating the defect-assisted recombination.
- The ACH^+^ cations can anchor to positively charged surface defects through the carbonyl group, forming a cationic dipole layer.
- The formation of an ACH^+^ cationic dipole layer induces surface polarization, which promotes favorable energy-level alignment and reduces interfacial energy losses.

**Supplementary Information:**

The online version contains supplementary material available at 10.1007/s40820-026-02150-7.

## Graphical abstract

**Figure:**
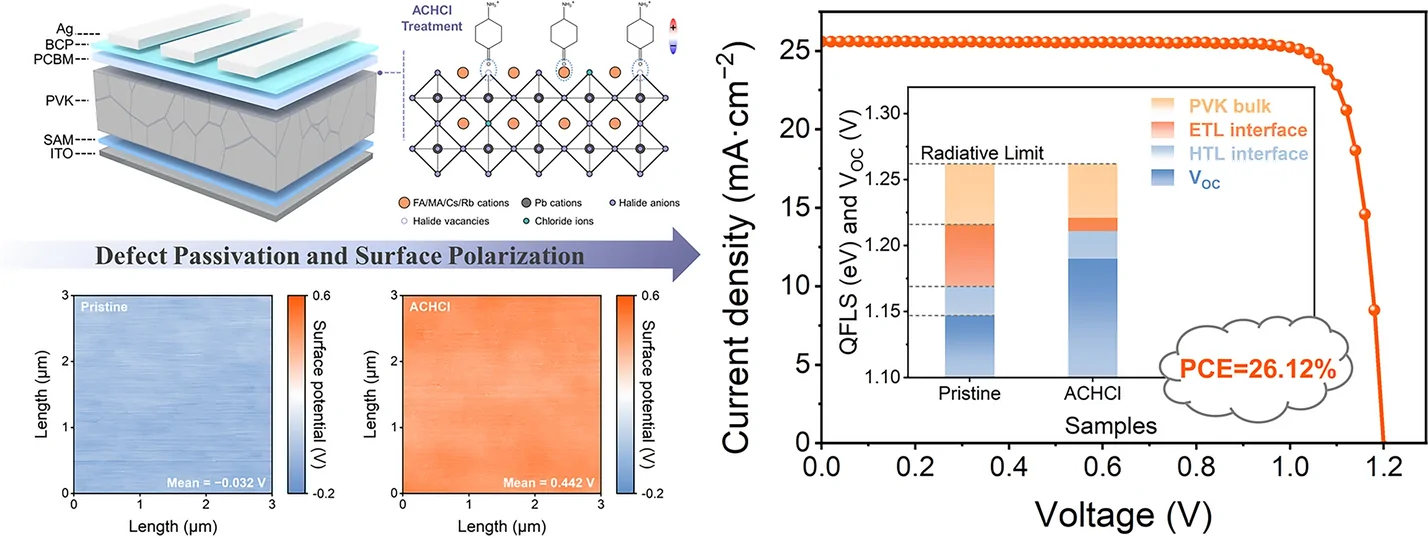

## Introduction

Lead halide amine perovskite solar cells (PSCs), recognized as the most popular next-generation photovoltaic technology, have rapidly increased their photovoltaic conversion efficiency (PCE) from 3.8% to more than 27% in just over a decade [1–3]. Especially, polycrystalline perovskite films have garnered significant attention due to the simplicity of their solution-based preparation methods and their unique photoelectronic features [4–6]. However, interfacial losses between the perovskite active layer (PVK) and the charge transport layers impede the further advancement of PSCs [7–9]. These interfacial losses are primarily composed of carrier non-radiative recombination losses caused by interface defects and energy losses resulting from energy-level misalignment at the interface.

Within the device structure of PSCs, there are two critical interfaces in direct contact with the perovskite film, namely PVK/electron transport layer (ETL) and PVK/hole transport layer (HTL) interfaces. For PSCs with inverted structure, the detrimental effect of loss at the PVK /HTL interface on devices has been significantly inhibited through the development of a wide variety of self-assembled materials [10–13]. However, the suppression of losses at the interface between the PVK and ETL remains the focus of research. To address this, surface modification has been extensively credited to be an effective strategy for reducing interfacial losses to enhance the performance of PSCs [14]. Specifically, a variety of multifunctional materials have been developed and introduced to the perovskite surface to reduce interfacial losses. Besides effectively reducing perovskite surface defects, these materials can also form a physical barrier to perovskite through strong coordination, thereby reducing the environmental impact and mitigating the degradation rate of device performance [15–17]. Through the surface treatment, new heterojunctions can also be formed on the perovskite surface, optimizing the energy-level arrangement to enhance carrier collection efficiency, thereby improving the photovoltaic performance of the PSCs [18–20]. In addition, studies aimed at optimizing the performance of PSCs based on the dipole effect have demonstrated that inducing the formation of permanent dipoles on the perovskite surface can reconstruct the surface energy of perovskite, thereby reducing charge accumulation at the interface [21–23]. Importantly, this dipole effect on the surface normally requires the presence of additional anchoring sites on the surface of the material. These anchoring points facilitate the oriented alignment of dipoles on the surface, leading to a directional redistribution of charges on the perovskite surface and inducing surface charge polarization in the perovskite [24, 25]. Therefore, to minimize the loss of interfacial defects and simultaneously optimize the interfacial energy, it is crucial to strategically utilize the surface defects of perovskite as anchoring points for dipoles to induce directional surface polarization.

In this work, we report a strategy to utilize perovskite surface defects as anchor points to induce surface polarization in perovskite films, which effectively improves the photovoltaic performance of PSCs. Due to the strong attraction between the positively charged defects on the perovskite surface and the carbonyl group, the ACH^+^ cations in 4-aminocyclohexanone hydrochloride (ACHCl) can effectively anchor to the perovskite surface through the carbonyl, resulting in the formation of a relatively ordered cation-dipole layer. Consequently, the ACHCl surface treatment not only effectively reduces surface defects in the perovskite film, thereby suppressing non-radiative recombination losses at the interface, but also induces surface polarization that positively modulates energy levels, ultimately leading to decreased charge accumulation at the interface. The effective suppression of interfacial losses resulted in a significant improvement in the performance of the optimized devices. As a result, after surface treatment with ACHCl, the PCE of inverted PSCs increased from 23.98% to 26.12%. Following 30 days of aging at a relative humidity of 30% ~ 40%, the ACHCl-modified device retained 91% of its initial PCE.

## Experimental Section

### Materials

Rubidium iodide (RbI), N,N-dimethylformamide (DMF, 99.8%), dimethyl sulfoxide (DMSO, 99.7%), chlorobenzene (CB, 99%), and isopropanol (IPA) were purchased from Sigma-Aldrich. Lead iodide (PbI_2_), lead bromide (PbBr_2_), cesium iodide (CsI), (2-(4-(bis(4-methoxyphenyl)amino)phenyl)-1-cyanovinyl)phosphonic acid (MPA-CPA), [6, 6]-phenyl-C61-butyric acid methyl ester (PCBM), and bathocuproine (BCP) were from Xi’an Yuri Solar Co., Ltd. Formamidinium iodide (FAI) and methylammonium bromide (MABr) were provided by Greatcell Solar Materials Pty Ltd. Indium tin oxide (ITO) substrates were supplied by Advanced Election Technology Co., Ltd. 4-aminocyclohexanone hydrochloride (ACHCl) and ethanol were obtained from Aladdin. All chemicals and solvents were obtained directly from the commercial suppliers and used as received without further purification.

### Device Fabrication

ITO glass substrates were sequentially cleaned by ultrasonic treatment in acetone and ethanol (20 min each), followed by exposure to UV ozone for 15 min. A self-assembled monolayer (SAM) was then introduced by spin-coating a 1.0 mg mL^−1^ ethanol solution of MPA-CPA at 3000 rpm for 30 s, followed by thermal annealing at 100 °C for 10 min. The perovskite absorber had the composition Rb_0.05_Cs_0.05_MA_0.05_FA_0.85_Pb(I_0.95_Br_0.05_)_3_, with a precursor solution concentration of 1.5 M. The precursor was prepared by dissolving stoichiometric amounts of RbI, CsI, MABr, FAI, PbI_2_, and PbBr_2_ in a mixed solvent of DMF and DMSO (4:1, v/v), followed by stirring for 2 h. A volume of 50 μL of the precursor solution was spin-coated onto the ITO/SAM substrate at 1000 rpm for 10 s and then at 3000 rpm for 40 s. During the final 15 s of the second step, 150 μL of CB was dropped onto the spinning substrate. The resulting film was annealed at 100 °C for 20 min to ensure complete solvent removal. For surface treatment, IPA solutions of ACHCl with varying concentrations (0.5, 1.0, 1.5, and 2.0 mg mL^−1^) were spin-coated onto the perovskite layer at 3000 rpm for 30 s, followed by thermal annealing at 100 °C for 10 min. After cooling to room temperature, an electron transport layer was deposited by spin-coating a 20.0 mg mL^−1^ PCBM solution in CB at 2000 rpm for 30 s. A 0.5 mg mL^−1^ solution of BCP in IPA was subsequently spin-coated at 5000 rpm for 30 s to form a BCP buffer layer. Finally, an 80-nm Ag electrode was deposited by thermal evaporation.

## Results and Discussion

### Molecular Selection and Its Impact on Photovoltaic Performance

For PSCs, precise modulation of interfacial band alignment is crucial for optimizing charge extraction and suppressing non-radiative recombination. As shown in Fig. S1, the Fermi level of the perovskite surface can be tuned by the orientation of interfacial dipoles [26]. Therefore, introducing a controllable dipole layer at the PVK/ETL interface in inverted PSCs facilitates the formation of an optimal band alignment, promotes efficient electron transport while effectively blocking holes, and ultimately enhances device performance.

To achieve this, three molecules with distinct dipole moments were introduced onto the perovskite surface to investigate their influence on device performance. The selected molecules included 1,4-cyclohexanedione (CHD), 4-aminocyclohexanecarboxylic acid (ACHA), and 4-aminocyclohexanone hydrochloride (ACHCl). As shown in Fig. S2, the electrostatic potential (ESP) distribution calculated using Multiwfn reveals that the CHD molecule possesses a symmetric structure with a dipole moment of 0 Debye [27, 28]. The regions of low electrostatic potential are primarily localized around the oxygen atoms of the two carbonyl (C = O) groups, indicating an electron-rich character that may facilitate the passivation of positively charged defects on the perovskite surface. Although the ACHA molecule exhibits an asymmetric structure, the presence of the carboxylic acid group reduces its overall polarity, resulting in a dipole moment of 3.9 Debye. The carboxylic acid group also features regions of low electrostatic potential, suggesting a moderate capability for defect passivation. In contrast, the ESP distribution of the ACH^+^ cation reveals the presence of a positively charged − NH_3_^+^ group and a negatively charged carbonyl group on the ACH^+^ cation, which exhibits notable dipolar characteristics. The oxygen atom in the carbonyl group exhibits a low electrostatic potential, which indicates a high electron cloud density. The calculated dipole moment of the ACH^+^ cation is 12.3 Debye, which may significantly influence the charge distribution on the perovskite surface.

The influence of the three molecular modifiers on the photovoltaic performance of the devices was evaluated through *J-V* measurements, as shown in Fig. S3 and Table S1. The CHD-treated device showed an increase in fill factor (FF), which is likely attributed to the effective passivation of positively charged defects on the perovskite surface by its dual carbonyl sites. However, the improvement in open-circuit voltage (*V*_*OC*_) remained limited. In contrast, treatment with ACHA, which possesses a weak dipole moment, resulted in a moderate increase in *V*_*OC*_ and a slight enhancement in FF. This suggests that although ACHA possesses a relatively weak dipole moment, its dipole effect likely contributes to the enhancement of the *V*_*OC*_. Notably, the ACHCl-treated device exhibited simultaneous increases in both FF and *V*_*OC*_. These improvements are likely attributed to the energy-level alignment optimization induced by the high dipole moment of the ACH^+^ cation, coupled with the synergistic passivation of surface defects by the carbonyl group and chloride ions. The statistical distribution of photovoltaic parameters shown in Fig. S4 and Table S2 further corroborates the trends described above. All three molecular modifiers improved the *V*_*OC*_ of the devices to varying extents. Compared to CHD, which lacks a dipole, the dipolar molecules ACHA and ACHCl induced a more evident increase in *V*_*OC*_, likely associated with dipole-induced adjustment of the perovskite interfacial energy levels.

To further elucidate the mechanisms by which different molecules influence the *V*_*OC*_ loss in devices, the quasi-Fermi-level splitting (QFLS) was evaluated through photoluminescence quantum yield (PLQY) measurements, providing a quantitative assessment of non-radiative recombination losses. As shown in Fig. S5, for samples with a glass/PVK/ETL structure, the PLQY values of samples treated with CHD, ACHA, and ACHCl increased from 1.22% in the pristine sample to 2.45%, 4.08%, and 6.17%, respectively. Correspondingly, the QFLS values rose from 1.169 eV in the pristine sample to 1.187, 1.201, and 1.211 eV, respectively. Notably, CHD, which lacks dipolar characteristics, shows a limited ability to reduce *V*_*OC*_ losses associated with non-radiative recombination. In contrast, the dipolar molecules ACHA and ACHCl more effectively suppress non-radiative recombination. In particular, the ACHCl-treated sample exhibits a *V*_*OC*_ loss due to non-radiative recombination that is 42 mV lower than that of the pristine sample. Although all three molecules help to mitigate non-radiative recombination losses, the synergistic passivation by the carbonyl and chloride ions in ACHCl, together with its dipole-induced adjustment of the band structure, likely contributes to the lowest degree of non-radiative recombination loss.

### Defect-Selective Anchoring and Molecular Orientation

To verify whether the ACH^+^ cations interact with the perovskite, the intermolecular forces are assessed using the interaction region indicator (IRI) function [29]. The potential interaction mechanism between ACH^+^ cation and FA^+^ cations is illustrated in Fig. S6, where the C = O group in ACH^+^ exhibits strong hydrogen bonding with FA^+^ cation, whereas the − NH_3_^+^ group shows a relatively weak interaction. This indicates that the C = O group on ACH^+^ cations adsorb onto FA^+^ cations via through hydrogen bonding, thereby effectively confining the migration of FA^+^ cations. Furthermore, Fig. S7 presents the interaction between ACH^+^ cation and PbI_2_, revealing a strong attraction between the C = O group and Pb atom, while the − NH_3_^+^ group exhibits weak interaction with the I atoms. The optimized interaction model between ACH^+^ cation and Pb^2+^ ion, further illustrated in Fig. S8, confirms a strong coordination interaction between the C = O group of ACH^+^ and the uncoordinated Pb^2+^ sites within the perovskite film, contributing to enhanced structural stability. Additional structural optimizations were carried out for the ACH^+^-FA^+^ and ACH^+^-PbI_2_ bimolecular models to disentangle the respective contributions of the C = O and − NH_3_^+^ functional groups of ACH^+^ to their interactions with FA^+^ and PbI_2_. The interaction between the ACH^+^ cation and the Pb^2+^ ion was also examined. The quantitative analyses indicate that the interactions involving the C = O group of ACH^+^ are consistently stronger than those mediated by the − NH_3_^+^ group. In particular, the interaction energies between the C = O group of ACH^+^ and FA^+^, PbI_2_, and Pb^2+^ are −0.21,−0.52, and − 0.66 eV, respectively. These findings demonstrate that coordination interactions dominated by the C = O group of ACH^+^ make a more important contribution than hydrogen bonding in the systems studied.

**Fig. 1:**
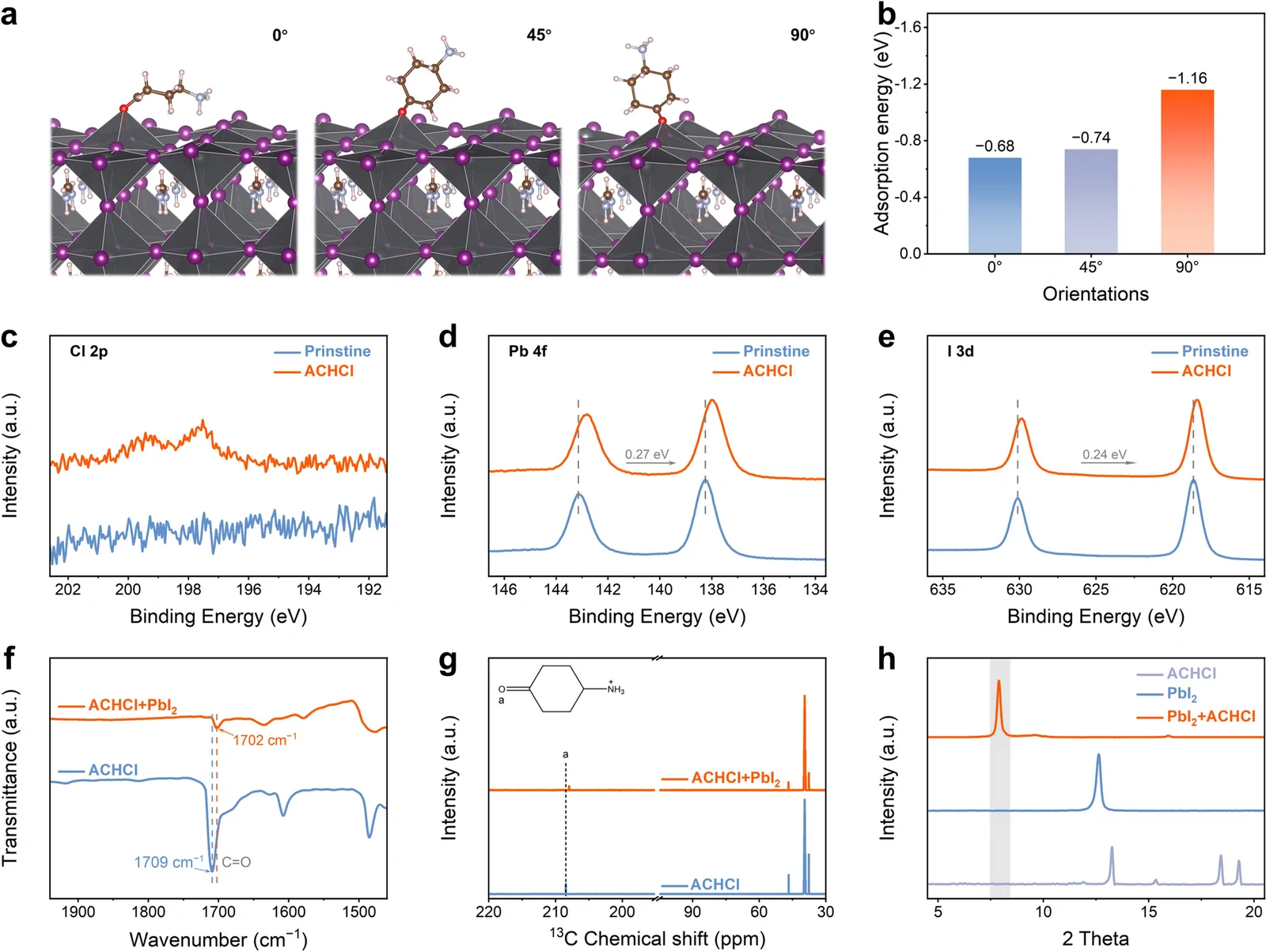
**a** Theoretical models and **b** calculated binding energies of the ACH^+^ cation adsorbed on the PbI_2_-terminated perovskite surface in different orientations. XPS spectra of **c** Cl 2*p*, **d** Pb 4*f*, and **e** I 3*d* for pristine and ACHCl-treated perovskite films. **f** FTIR spectra of ACHCl + PbI_2_ and ACHCl. **g** ^13^C NMR spectra of ACHCl + PbI_2_ and ACHCl in DMSO-d_6_ solution. **h** XRD patterns of ACHCl + PbI_2_, PbI_2_, and ACHCl

The interactions between the ACH^+^ cation and the perovskite are further assessed using density functional theory (DFT) [30]. To investigate the anchoring behavior and orientation mechanism of the ACH^+^ cation on the perovskite surface, the adsorption configurations of the molecule were systematically modeled in three distinct spatial orientations, with the six-membered ring plane forming angles of 0°, 45°, and 90° relative to the PbI_2_-terminated perovskite surface. The optimized adsorption models indicate that, in all orientations, the carbonyl group of the ACH^+^ cation forms Pb − O bonds with uncoordinated Pb atoms on the perovskite surface. As shown in Fig. 1a, b, the vertical orientation (90°) exhibits the lowest adsorption energy (E_A90°_ =  −1.16 eV), significantly more negative than the parallel (0°, E_A0°_ =  −0.68 eV) and inclined (45°, E_A45°_ =  −0.74 eV) configurations, demonstrating a clear preference for vertical alignment of the ACH^+^ cation on the perovskite surface. Further analysis of the Pb − O bond length trends shown in Fig. S9 provides a clearer insight into the impact of molecular orientation on interfacial bonding strength. In the parallel configuration (0°), the Pb − O bond length extends to 2.86 Å, indicating a relatively weak interaction. This is likely due to insufficient orbital overlap between the carbonyl group and the Pb ions, as well as electrostatic repulsion between the − NH_3_^+^ group of the cation and the perovskite surface. As the molecular orientation increases to 45° and 90°, the Pb − O bond lengths decrease to 2.65 and 2.39 Å, respectively, indicating enhanced coordination between the carbonyl oxygen and surface lead ions. The consistency between bond length and adsorption energy results demonstrates that the ACH^+^ cation not only forms shorter Pb − O bonds in the vertical orientation but also achieves the most stable adsorption configuration.

Based on the preferred adsorption orientation identified above, the adsorption behavior of the ACH^+^ cation on FAI-terminated and mixed-terminated perovskite surfaces was further analyzed. The optimized structures (Fig. S10) reveal that ACH^+^ adsorbs on the FAI-terminated surface with an adsorption energy of −0.50 eV. Notably, the oxygen atom of the carbonyl group in ACH^+^ forms hydrogen bonds with two neighboring FA^+^ cations, with bond lengths of 1.81 and 1.96 Å, respectively. The formation of such hydrogen-bonding interactions can stabilize the FA^+^ cations, thereby increasing the energy for cation migration and vacancy formation, as has been widely validated in the literature [31–33]. Differential charge-density analysis (Fig. S11) further reveals pronounced charge transfer between ACH^+^ and the bonded FA^+^ species, providing electronic-level evidence that ACH^+^ can effectively anchor FA^+^ via hydrogen bonding, thereby suppressing FA^+^ migration and vacancy formation. The mixed-terminated perovskite surface models that simultaneously include Pb-deficient and FA-deficient regions were constructed to explicitly probe the feasibility of multi-site interactions under realistic lattice constraints. As shown in Fig. S12, the results show that the ACH^+^ cations preferentially bind to under-coordinated Pb sites via the carbonyl oxygen, with an adsorption energy of −0.68 eV. The resulting Pb − O bond length is approximately 2.55 Å, indicative of a strong coordination interaction. However, the shortest distance between the carbonyl oxygen of ACH^+^ and the hydrogen atoms of − NH_2_ group in the neighboring FA^+^ is 2.93 Å, which exceeds the typical range for effective hydrogen bonding. This result demonstrates that, when the carbonyl group coordinates to Pb^2+^, ACH^+^ cannot simultaneously form a strong hydrogen bond with FA^+^ due to geometric constraints. From Fig. S13, differential charge-density analyses further reveal pronounced charge redistribution between ACH^+^ and the Pb − I framework, confirming the dominant role of Pb − O coordination in this configuration. In contrast, only weak electronic interaction is observed between ACH^+^ and nearby FA^+^ cations in this configuration. These results demonstrate that, under lattice-constrained conditions, ACH^+^ does not simultaneously coordinate Pb^2+^ and hydrogen bond with FA^+^ through the same carbonyl oxygen. Instead, these interactions occur independently on different surface terminations. The carboxyl group predominantly coordinates with Pb^2+^ on Pb-rich surfaces, whereas hydrogen bonding between the carboxyl group and FA^+^ dominates on FA-terminated surfaces. The theoretical results reveal that the interactions between ACH^+^ and Pb^2+^, as well as between ACH^+^ and FA^+^, do not occur through a simultaneous multi-site interaction configuration. These interactions act in a complementary manner on the perovskite surface, depending on the local surface termination and defect environment. From a theoretical perspective, these results further confirm that the ACH^+^ cations preferentially anchor to the perovskite surface via their carbonyl groups, through coordination with Pb^2+^ or hydrogen-bonding interactions with FA^+^, while the − NH_3_^+^ groups orient outward.

Theoretically, ACH^+^ cations can adopt a relatively ordered arrangement on the perovskite surface by anchoring to positively charged defects. To further determine the molecular orientation of the ACH^+^ cation on the perovskite surface, angle-resolved X-ray photoelectron spectroscopy (AR-XPS) was employed to analyze the variations in the N 1*s* and C 1*s* signals of ACH^+^ at different probing depths. The corresponding results are presented in Figs. S14 and S15. Measurements were performed at electron take-off angles of 0°, 30°, and 60°, with larger take-off angles corresponding to shallower probing depths. In the N 1*s* spectra, the ratio of the C − N signal originating from the ACH^+^ cation to the N signal from formamidinium (FA) gradually increases as the probing depth decreases. Similarly, in the C 1*s* spectra, the ratios of the C − N signal associated with ACH^+^ and the C = N signal from FA to the C − C calibration peak (284.8 eV) also increase with shallower probing depths. Conversely, the ratio of the C = O signal from ACH^+^ to the C − C calibration peak decreases at shallower depths. These observations further confirm the molecular orientation of the ACH^+^ cation on the perovskite surface. The molecules are predominantly anchored to positively charged surface defect sites via the carbonyl (C = O) group, while the − NH_3_^+^ moiety is oriented outward. This result provides direct evidence that, even under realistic surface coverage conditions, the arrangement of ACH^+^ cations remains relatively ordered.

### Interaction Mechanisms Governing Defect Passivation

To investigate the actual impact of ACHCl surface treatment on the surface structure of perovskite film, the X-ray photoelectron spectroscopy (XPS) technique is utilized to examine different perovskite films [34], as illustrated in Fig. S16. From Fig. 1c, an XPS signal for Cl 2*p* is detected on the surface of the ACHCl-modified perovskite film, whereas this signal is absent on the surface of the pristine perovskite film. Meanwhile, the N 1*s* XPS spectra presented in Fig. S17 reveal a shoulder peak around 401.5 eV on the surface of the ACHCl-modified perovskite film, which should be attributed to the C − N bond of ACHCl. These additional observed XPS signals confirm the successful introduction of ACHCl onto the surface of the perovskite film. In the C 1*s* XPS spectra shown in Fig. S18, the signal corresponding to the C = O bond of CO_2_ observed in the pristine perovskite film is absent in the ACHCl-modified perovskite film, while a new signal assignable to the carbonyl C = O bond of ACHCl emerges. This observation indicates that the incorporation of ACHCl effectively mitigates the degradation of the perovskite layer. Furthermore, after the introduction of ACHCl, the N 1*s* XPS spectra also reveal that the C = N bond associated with FA^+^ shifts toward lower binding energy. This shift is likely due to hydrogen bonding (N − H∙∙∙O or N − H∙∙∙Cl) between the ACHCl and FA^+^, which alters the electron cloud density around the nitrogen atom, thereby contributing to suppress the formation of FA^+^ vacancies [35, 36]. In Fig. 1d, the shift of the Pb 4*f* XPS signal of the ACHCl-modified perovskite film toward lower E_B_ indicates an increase in the electron cloud density around the Pb atoms [37]. This shift should be attributed to the coordination between the C = O groups or Cl^−^ ions of ACHCl and the under-coordinated Pb^2+^ ions, which can effectively inhibit defects caused by halogen vacancies. Similarly, a shift toward lower E_B_ can be also observed in the I 3*d* XPS signal shown in Fig. 1e, further supporting the interaction between ACHCl and the Pb − I framework, which affects the overall electron cloud density of the [PbI_6_]^4−^ octahedra [38]. The results of XPS characterizations confirm that the introduction of ACHCl inhibits the formation of surface defects in perovskite films and induces alterations in the electron cloud density on the perovskite surface.

To further validate the interaction between ACHCl and the perovskite surface, the interactions between ACHCl and both FAI and PbI_2_ were verified. The liquid-state NMR spectra in Fig. S19 confirm the interaction between ACHCl and FAI. After the introduction of ACHCl, the ^1^H NMR spectra show that the resonance peak corresponding to the − NH_2_ groups of FAI splits into two distinct signals, while the − CH resonance evolves into a more complex multiplet, indicating the presence of hydrogen-bonding or electrostatic interactions between FAI and ACHCl. Moreover, the signal associated with the − NH_3_^+^ group in ACHCl shifts upfield, indicating a weakening of the hydrogen-bonding environment surrounding the − NH_3_^+^. This behavior can be attributed to partial anion redistribution upon mixing with FAI, whereby Cl^−^ ions in the vicinity of − NH_3_^+^ are partially replaced by I^−^ anions, which are weaker hydrogen-bond acceptors, thereby reducing the deshielding effect. Fourier-transform infrared spectroscopy (FTIR) further confirms the coordination interaction between the ACHCl and PbI_2_, as illustrated in Fig. 1f. ACHCl exhibits a characteristic peak corresponding to the C = O stretching vibration at 1709 cm^−1^. After interaction with PbI_2_, this peak shifts to 1702 cm^−1^, indicating the formation of coordination interactions between the Pb^2+^ ions and C = O groups of ACHCl [37]. The observed upfield shift of the carbon signal corresponding to the C = O group in the liquid-state NMR spectra, presented in Fig. 1g, indicates an increase in electron density around the carbonyl carbon. This change is attributed to a coordination interaction between the C = O group of ACHCl and PbI_2_.

To deeply investigate this interaction, PbI_2_ and ACHCl at a mass ratio of 1:5 are dissolved in a DMF-DMSO mixed solution (volume ratio, 9:1). The digital photographs in Fig. S20 show a significant color change in the mixed solution compared to the pure PbI_2_ solution, which is supposed to be the result of the interaction between PbI_2_ and ACHCl. Additionally, the different solutions mentioned above were deposited onto ITO substrates using a spin-coating method, followed by annealing at 70 °C for 1 min. The material structures are identified using X-ray diffraction (XRD) techniques. As observed in Fig. 1h, the mixture of PbI_2_ and ACHCl exhibits a distinct diffraction peak in the 7° ~ 8° range, different from those of the pure PbI_2_ and pure ACHCl samples. This suggests the formation of a new substance by mixing PbI_2_ and ACHCl, further confirming the presence of the interaction between PbI_2_ and ACHCl. Based on theoretical and experimental investigation, it can be inferred that the interaction between ACHCl and perovskite primarily arises from the strong attraction between the electron-rich carbonyl groups and Pb ions. This interaction facilitates the anchoring of ACH^+^ cations to the positively charged defects on the perovskite surface, inducing surface charge polarization of the perovskite film by forming a relatively ordered molecular arrangement.

XRD analysis (Fig. S21) demonstrates the crystal structure of the perovskite samples before and after the introduction of ACHCl. From Fig. S22, with the increase in ACHCl concentrations, the diffraction peak intensity of the (100) crystal plane of the perovskite samples is enhanced, and the position of the diffraction peak shifts accordingly. A similar shift is also observed for the (200) crystal plane, which may be because Cl^−^ ions fill the halide vacancies, causing the surrounding atoms to move inward and thereby resulting in lattice compression [39]. To elucidate the localized distribution of Cl^−^ ions within the perovskite film and their potential role in vacancy passivation, elemental mapping of Cl was performed using energy-dispersive spectroscopy (EDS) on the cross-sectional SEM image of the ACHCl-treated perovskite film. Figure S23 clearly reveals that Cl elements are predominantly distributed near the surface of the perovskite film and gradually decrease toward the ITO substrate. This observation indicates that the Cl^−^ ions introduced by ACHCl are mainly localized near the upper surface of the perovskite layer, where they may help to fill the halide vacancies on the perovskite surface.

The ultraviolet–visible (UV–Vis) absorption spectra and Tauc plots shown in Figs. S24 and S25 indicate that the introduction of ACHCl does not affect the optical absorption and the band gap of the perovskite materials. The Urbach energy (*E*_*U*_) obtained from the UV–Vis absorption spectrum can be used to evaluate the degree of disorder and crystallinity in a material [40]. The smaller the *E*_*U*_, the better the crystallinity of the material. The *E*_*U*_ values of the perovskites treated with different concentrations of ACHCl are shown in Fig. S26, and the *E*_*U*_ values of ACHCl-treated perovskite are lower compared to the pristine perovskite sample, with the most significant reduction observed in the sample treated with a concentration of 1.5 mg mL^−1^. This suggests that a concentration of 1.5 mg mL^−1^ ACHCl is more conducive to enhancing the crystallinity of the perovskite material without inducing negative effects. To further confirm that 1.5 mg mL^−1^ is the optimal ACHCl concentration, twenty devices were fabricated under each of several different concentrations, and their *J-V* characteristics were measured. The statistical distribution of the photovoltaic parameters is presented in Fig. S27 and Table S3. It is observed that as the concentration increases to 1.5 mg mL^−1^, both the average fill factor and average *V*_*OC*_ gradually improve. However, when the concentration exceeds 1.5 mg mL^−1^, reaching 2.0 mg mL^−1^, device performance declines. Although device performance degrades at excessive ACHCl concentrations relative to the optimal concentration, the overall performance at 2.0 mg mL^−1^ remains superior to that of the pristine device. This phenomenon indicates that a relatively ordered molecular orientation is preserved, whereas excessive molecular accumulation likely impedes charge transport. Therefore, subsequent characterization and analysis will primarily focus on this optimal concentration of 1.5 mg mL^−1^.

### Energy-Level Reconstruction Induced by Surface Polarization

**Fig. 2:**
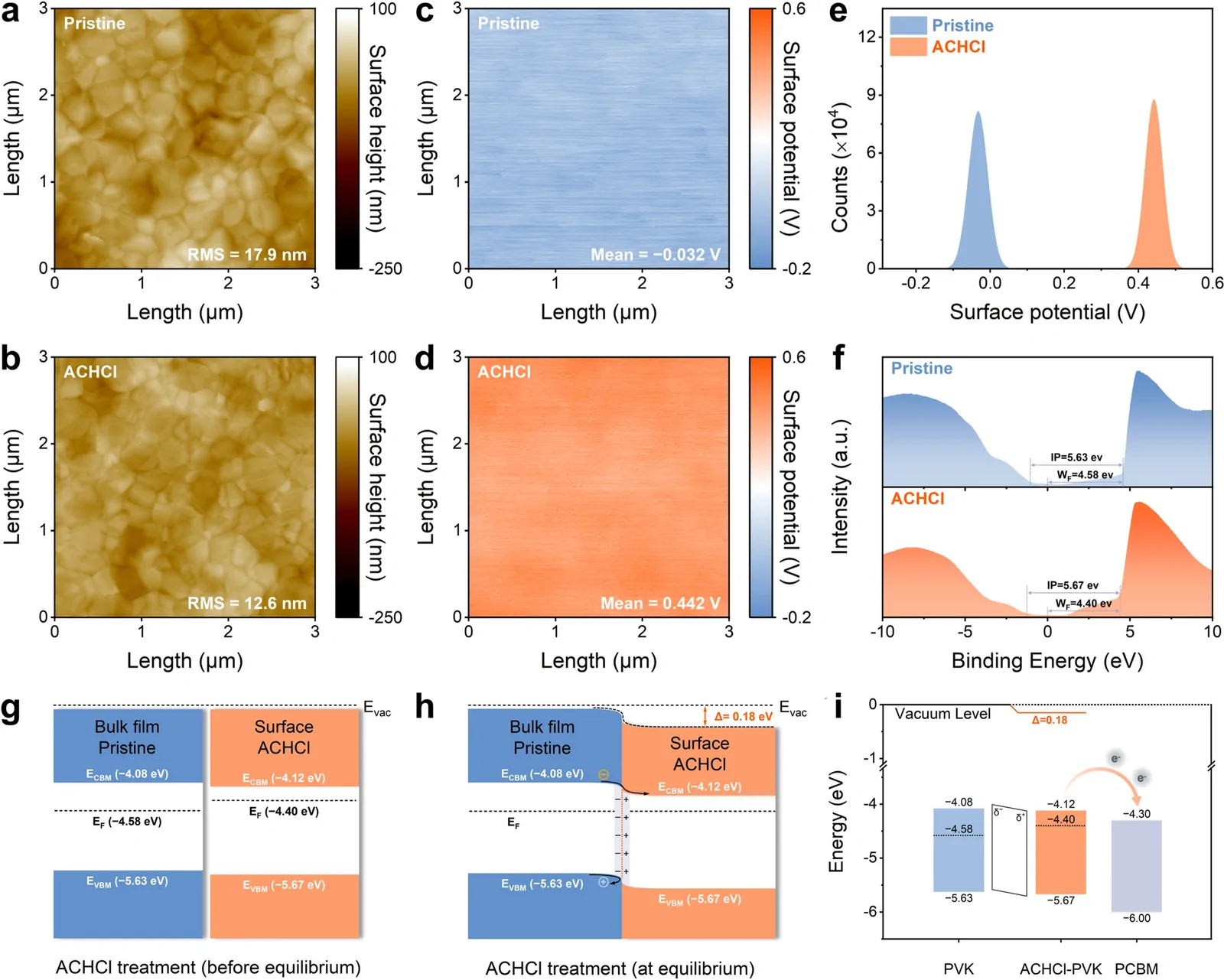
AFM images of **a** pristine and **b** ACHCl-treated perovskite films. KPFM images of **c** pristine and **d** ACHCl-treated perovskite films. **e** Statistical distribution of the surface potential. **f** UPS spectra of pristine and ACHCl-treated perovskite films. **g** Energy levels of pristine and ACHCl-treated perovskite films (before equilibrium), and **h** the corresponding band-bending schematics at equilibrium. **i** Energy band diagram of PCBM layer and different perovskite films

The scanning electron microscope (SEM) images of pristine and ACHCl-treated perovskite films presented in Fig. S28. The surface of the ACHCl-treated perovskite film exhibits significant change compared to the pristine perovskite film, with the grain boundaries becoming less distinct. To further observe the alteration of the surface morphology of perovskite films after ACHCl treatment, the surface roughness of different perovskite films is measured using atomic force microscopy (AFM) technique. In Fig. 2a, b, the root-mean-square (RMS) roughness of the ACHCl-treated perovskite film and the pristine perovskite film is measured to be 12.6 and 17.9 nm, respectively. The reduction in roughness is beneficial for improving the contact between the PVK layer and the PCBM layer, thereby reducing carrier recombination at the interface.

Figure 2c, d illustrates the surface potential of different perovskite films. The ACHCl-treated perovskite film exhibits a higher surface potential compared to the pristine perovskite film. Furthermore, the surface potential distribution in Fig. 2e shows that the surface potential of the ACHCl-treated perovskite film is narrower distributed, indicating that ACHCl treatment leads to a more uniform surface potential across the perovskite film. The significantly increased surface potential proves that the introduction of ACHCl induces a redistribution of charges on the perovskite surface. This effect can be attributed to the formation of a relatively ordered cation-dipole layer by ACH^+^, which directionally modulates the surface charge distribution and induces charge polarization in the film surface. Specifically, carbonyl groups with rich electron characteristics anchor to positively charged defects on the perovskite surface, while the positively charged − NH_3_^+^ groups are oriented toward the exterior of the perovskite layer. This arrangement results in a high density of positive charges on the perovskite surface, which leads to an elevated surface potential of the perovskite film. The surface potential distribution supports the scenario that ACHCl induces surface polarization on the perovskite surface while also confirms that the distribution of ACH^+^ cations is relatively consistent.

To evaluate the impact of ACHCl on the surface energy-level structure of perovskite, ultraviolet photoelectron spectroscopy (UPS) technique is used. From the UPS spectra in Fig. 2f, the introduction of ACHCl results in a reduction in the work function (W_F_) of the perovskite surface, which is consistent with the observed increase in surface potential [41]. The ionization potential (IP) represents the energy difference between the vacuum level (E_vac_) and the valence band maximum (E_VBM_). After ACHCl modification, the IP value of the perovskite increases from 5.63 to 5.67 eV. Relative to the E_VBM_, the Fermi-level (E_F_) position relatively shifts upward from − 4.58 to − 4.40 eV, indicating a significant transition in the surface energy of the perovskite film from n-type to n^+^-type. This transition should be attributed to surface polarization induced by ACHCl, which causes a redistribution of charges on the perovskite surface and subsequently leads to a reconstruction of the surface energy of the perovskite. Based on the energy-level parameters shown in Table S4, the energy-level diagrams are drawn and shown in Fig. 2g, h. Notably, the ACHCl modification results in a downward band bending of the perovskite surface, which facilitates electron transfer and hole block. Referring to the energy-level parameters of PCBM reported in the previous literature [42], Fig. 2i shows that the conduction band minimum (E_CBM_) of the ACHCl-modified perovskite is closer to the E_CBM_ of the PCBM layer compared to the pristine perovskite. The reduction in E_CBM_ arises from surface polarization induced by the cation-dipole layer, which causes downward band bending from the perovskite bulk to the perovskite surface. Consequently, the energy barrier at the PVK/PCBM interface is reduced, favoring electron extraction from PVK into PCBM. The KPFM and UPS characterizations demonstrate that by rationally utilizing dipole effects to induce the surface polarization of perovskite film, a perovskite surface favorable for carrier transport can be effectively constructed, thereby reducing energy losses at the interface due to energy-level mismatch.

### Decoupling the Effects of Defect Passivation and Surface Polarization

**Fig. 3:**
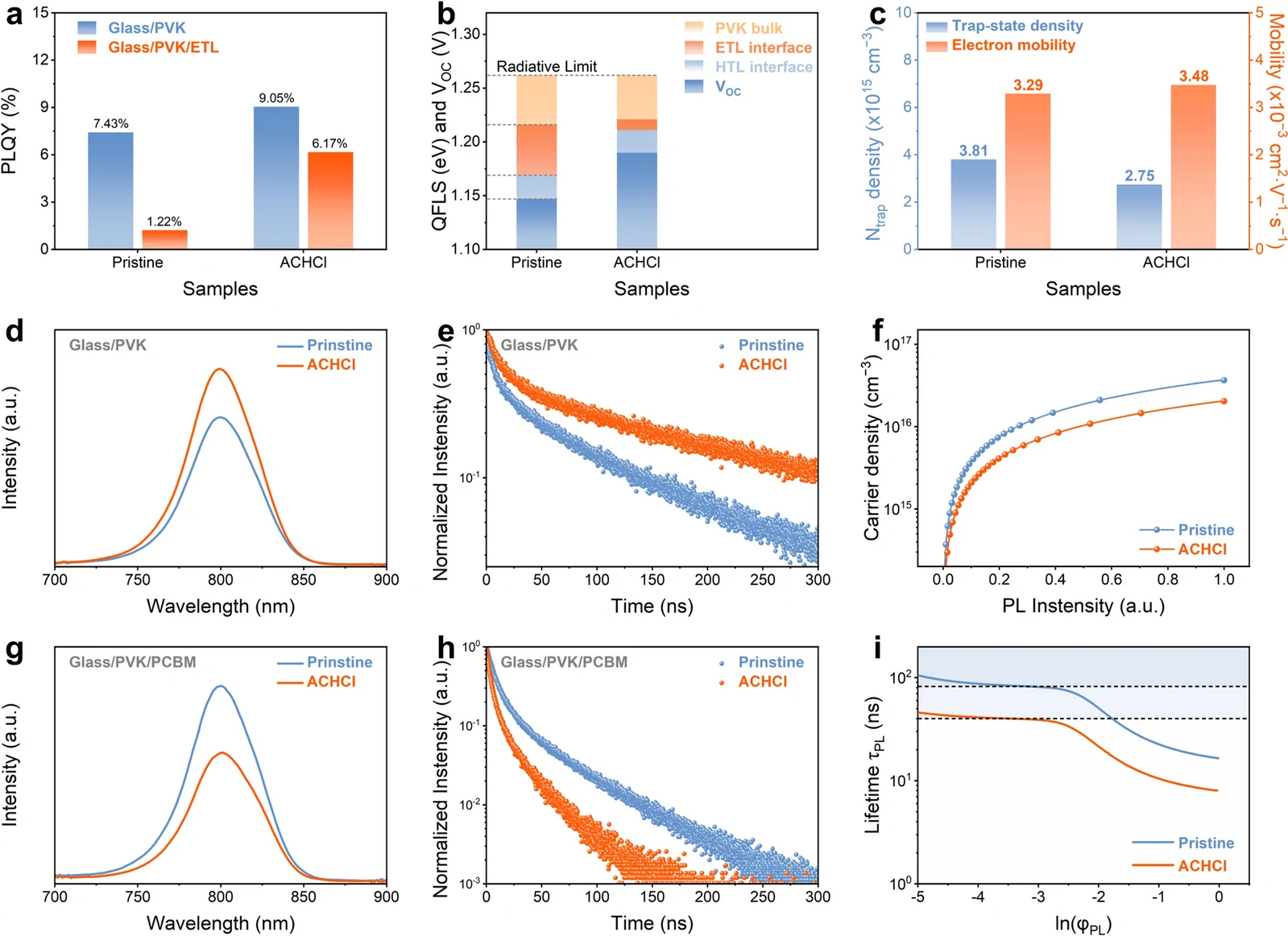
**a** PLQY values of pristine and ACHCl-treated samples with different structures. **b** *V*_*OC*_ loss mechanism of pristine and ACHCl-treated samples. **c** Trap-state density and electron mobility of different devices. **d** PL and **e** TRPL spectra of the samples with glass/PVK structure. **f** Photogenerated carriers versus the PL intensity. **g** PL and **h** TRPL spectra of the samples with glass/PVK/PCBM structure. **i** Photoluminescence lifetime versus the logarithm of PL intensity

The contributions of defect passivation and surface polarization were further distinguished by PLQY measurements, as presented in Fig. 3a. For the glass/PVK samples, ACHCl treatment increases the PLQY from 7.43% to 9.05%, indicating effective suppression of defect-assisted non-radiative recombination within the perovskite film. This improvement is attributed to interactions between ACH^+^ and Pb^2+^/FA^+^, as well as the occupation of I^−^ vacancies by Cl^−^ ions, which together lead to effective defect passivation. These results directly reflect the contribution of defect passivation to the improved optoelectronic quality of the perovskite layer. In contrast, when an ETL is introduced, the PLQY of the pristine sample decreases sharply from 7.43% to 1.22%, revealing substantial interfacial carrier losses at the PVK/ETL interface. Notably, the ACHCl-treated sample exhibits a much smaller reduction, from 9.05% to 6.17%. This pronounced difference indicates that ACHCl treatment substantially mitigates interfacial energy losses and enhances charge-carrier extraction efficiency at the PVK/ETL interface. Previous molecular screening studies indicate that, compared with defect passivation, dipole-induced effects are more effective in reducing *V*_*OC*_ losses in devices. Therefore, such a pronounced reduction in interfacial PLQY loss cannot be attributed solely to defect passivation within the perovskite film. Instead, it points to a marked enhancement of interfacial charge-carrier extraction and transport, which may be primarily attributed to surface polarization induced by ACH^+^ dipoles at the perovskite surface. This surface polarization can effectively reduce the energy barrier for carrier extraction and transport.

Further analysis of the *V*_*OC*_ loss mechanisms supports this distinction, as shown in Fig. 3b. While ACHCl treatment reduces non-radiative recombination within the perovskite film through defect passivation, its effect on suppressing interfacial recombination losses at the PVK/ETL interface is substantially stronger. This observation underscores the dominant role of surface polarization in improving interfacial charge transport and reducing energy loss at the PVK/ETL interface. Therefore, the improvement in the intrinsic optoelectronic properties of the perovskite films can be attributed to the interactions between ACH^+^ and Pb^2+^/FA^+^, as well as the occupation of I^−^ vacancies by Cl^−^ ions, which together result in effective defect passivation. Meanwhile, the enhanced carrier transport and reduced energy loss at the PVK/ETL interface are primarily attributable to the surface polarization effect. Based on the PLQY and QFLS analyses, it can be concluded that ACHCl treatment effectively suppresses non-radiative recombination losses in both the perovskite bulk and the PVK/ETL interface, thereby offering the potential to reduce the *V*_*OC*_ loss and FF of the device.

To more intuitively evaluate the defect states of the devices, the space-charge-limited current (SCLC) technique is used. From Fig. S29, the ACHCl-modified electron-only device with the structure of ITO/SnO_2_/PVK/PCBM/Ag shows a smaller trap-filled limit voltage (*V*_*TFL*_) compared to the pristine electron-only device, which suggests that the introduction of ACHCl can effectively reduce the trap-state density of the device [43]. The trap density estimated from SCLC analysis primarily represents the total density of electrically active traps that impede charge transport under space-charge-limited conditions, thereby reflecting the integrated impact of these traps on carrier transport. Based on the SCLC measurement of electron-only devices, the impact of ACHCl treatment on electron transport was investigated, and the electron mobilities were determined (Fig. S30). Figure 3c illustrates that the ACHCl-treated electron-only device exhibits a lower defect-state density and enhanced electron mobility. The SCLC measurement indicates that the introduction of ACHCl effectively reduces surface defects and optimizes the energy-level structure by surface polarization, thereby improving electron extraction in the device.

To assess the influence of surface treatment on the charge-carrier transport dynamics of the PVK layer, steady-state photoluminescence (PL) and time-resolved photoluminescence (TRPL) characterization are used. Figure 3d shows that the PL intensity of the ACHCl-treated perovskite film is higher than that of the pristine perovskite film, indicating a reduction in defect-induced non-radiative recombination after surface treatment [44]. From the TRPL spectra in Fig. 3e and the fitted parameters in Table S5, the average carrier lifetime (*τ*_*avg*_) of the ACHCl-treated perovskite film is extended, likely as a result of surface treatment suppressing defect recombination on the perovskite surface [45, 46]. Furthermore, a theoretical model correlating photoinjected carrier density with integrated PL intensity was further used to fit the TRPL data and evaluate the trap density in different perovskite films [47]. In Fig. 3f, the curves of the integrated PL intensity versus photoinjected carrier density different samples reveal that the injected carrier density in the ACHCl-treated perovskite film is significantly lower than that in the pristine perovskite film, which should be attributed to the lower defect density in the perovskite film after surface treatment.

Given the close correlation between defect states and ion migration in lead halide perovskites, the influence of the reduced defect density induced by ACHCl treatment on ion migration was further investigated. As shown in Fig. S31, PL spectra of pristine and ACHCl-treated perovskite films were recorded under continuous one-sun illumination. After 12 h of illumination, the pristine film exhibits a pronounced red shift in the PL peak, indicating the formation of iodide-rich, low-bandgap domains. In contrast, the ACHCl-treated film exhibits only a negligible spectral shift, demonstrating that ACHCl treatment effectively suppresses halide ion migration and associated phase segregation. To quantitatively assess ion migration, temperature-dependent conductivity measurements were performed on devices with an ITO/PVK/Ag structure to extract the ion migration activate energy (E_a_). From Fig. S32, the ACHCl-treated perovskite film exhibits a higher E_a_ (0.225 eV) than the pristine film (0.153 eV), confirming that ACHCl treatment effectively increases the energetic barrier for ion migration. A higher activation energy implies a reduced propensity for the formation of ionic defects in both the bulk and interfacial regions of the perovskite, which is beneficial for mitigating device degradation and improving operational stability.

To investigate the effect of ACHCl introduction on carrier transport at the PVK/ETL interface, the heterojunctions with a glass/PVK/PCBM structure were fabricated. Figure 3g, h demonstrates that the ACHCl-modified heterojunction exhibits lower PL intensity and faster carrier quenching compared to the pristine heterojunction, indicating that the introduction of ACHCl facilitates improved carrier extraction from the PVK layer into the PCBM layer [44, 48]. Although the enhanced carrier extraction efficiency can partly be attributed to defect passivation, which reduces surface defect density, it primarily arises from a surface polarization effect, which optimizes the energy-level alignment at the PVK/ETL interface and thereby promotes carrier extraction and transport. The photoluminescence lifetime curve based on TRPL decay can further distinguish the effects of the bulk and the interface on carrier transport in the perovskite film. Figure S33 directly confirms effective carrier transfer at the ACHCl-treated interface. In the relationship between photoluminescence lifetime (*τ*_*PL*_) and the logarithmic TRPL intensity (*φ*_*PL*_) depicted in Fig. 3i, the *τ*_*PL*_ plateau of the devices is influenced by the complex interaction between the bulk Shockley–Read–Hall (SRH) lifetime and the surface recombination velocity [49]. Notably, the effective SRH lifetime of the ACHCl-modified device is significantly lower than that of the pristine device, indicating a higher surface recombination velocity. In devices with an ETL, the surface recombination velocity reflects the efficiency of electron extraction from the PVK layer to the PCBM layer. The relationship between *τ*_*PL*_ and *φ*_*PL*_ demonstrates that the surface polarization induced by ACHCl is beneficial for carrier transport at the PVK/PCBM interface.

### Charge-Carrier Dynamics in Devices

**Fig. 4:**
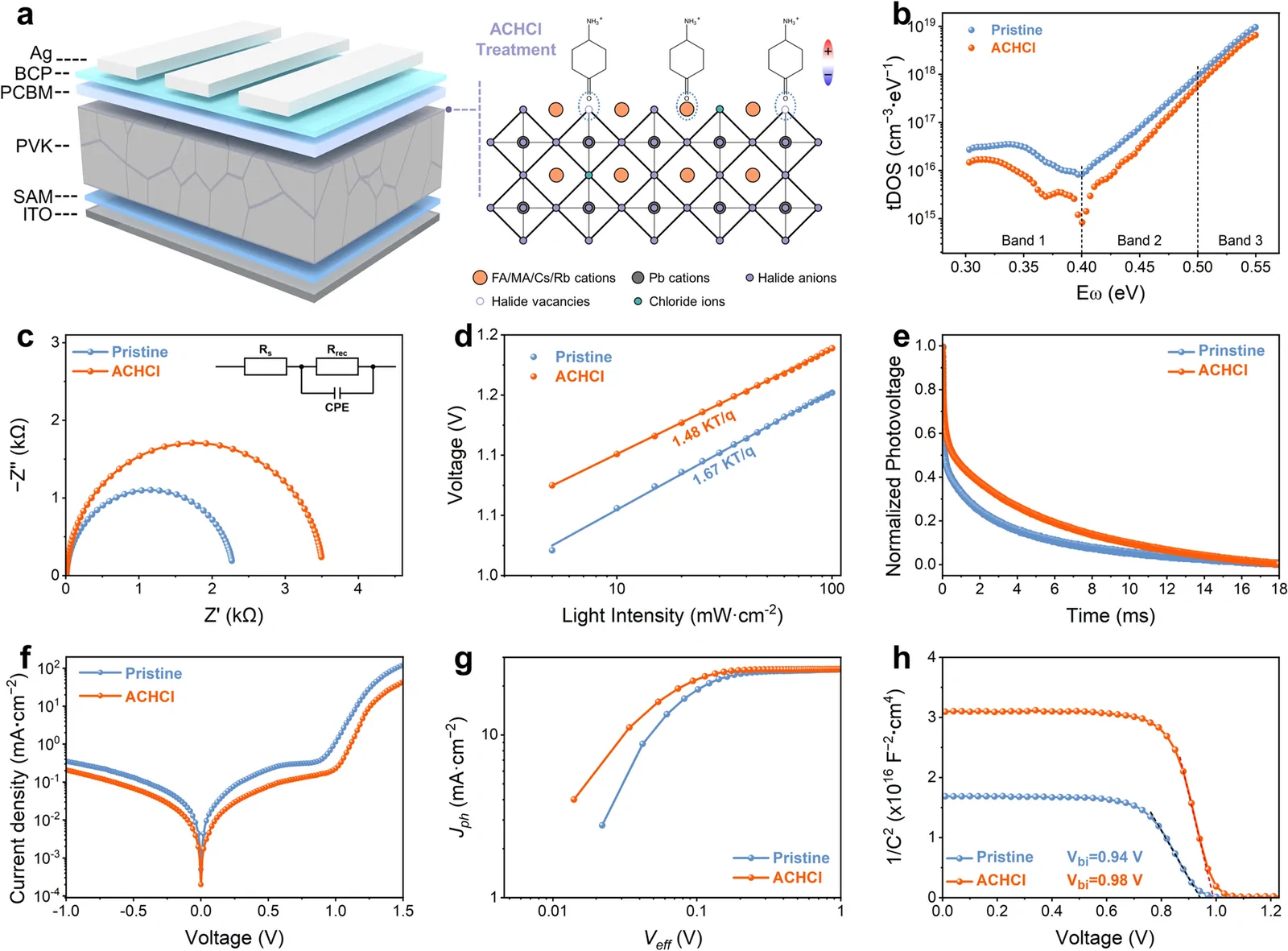
**a** Schematic diagram of the device structure and the mechanism by which ACHCl treatment induces surface polarization via defect-anchored dipole molecules. **b** tDOS obtained from admittance spectroscopy of pristine and ACHCl-treated devices. **c** Impedance profiles of pristine and ACHCl-treated devices in the dark state. **d** *V*_*OC*_ versus light intensity curves for pristine and ACHCl-treated devices. **e** TPV curves of pristine and ACHCl-treated devices. **f** *J-V* curves of pristine and ACHCl-treated devices in the dark state. **g** Photocurrent versus effective bias characteristics curves for pristine and ACHCl-treated devices. **h** M-S curves of pristine and ACHCl-treated devices

Figure 4a illustrates the device structure of the ACHCl-treated inverted PSCs, along with a schematic illustration of the mechanism by which ACHCl treatment induces surface polarization via defect-anchored dipole molecules. Benefiting from the molecular structure of ACHCl, its introduction can suppress uncoordinated Pb^2+^ defects and halogen vacancies through the carbonyl groups and chloride ions. By stabilizing the octahedral structure of lead halide, ACHCl effectively prevents the collapse of the perovskite lattice. Furthermore, the strong electron-donating properties of the carbonyl group, owing to its lone pair electrons, enables ACH^+^ cations to anchor onto uncoordinated Pb^2+^ ions and FA^+^ cations via coordination and hydrogen bonding, respectively. This allows the ACH^+^ cation to be firmly positioned on the perovskite surface with the − NH_3_^+^ groups oriented outward. The formation of this relatively ordered cationic dipole layer leads to charge redistribution at the perovskite surface, thereby inducing surface polarization. The interactions of ACH^+^ with Pb^2+^ and FA^+^, together with the filling of halogen vacancies by Cl^−^ ions, synergistically result in effective defect passivation. This defect passivation enhances the structural stability of the perovskite and suppresses trap-assisted non-radiative recombination in both the bulk and at the surface, which is expected to contribute to an improved FF of the devices and enhanced device stability. Meanwhile, surface polarization induced by a relatively ordered cationic dipole layer optimizes the energy-level alignment between the perovskite and PCBM, thereby facilitating electron extraction while simultaneously blocking hole transport. This surface polarization effect significantly reduces interfacial energy losses, which is beneficial for minimizing *V*_*OC*_ losses in the devices. Collectively, these complementary effects may synergistically enhance device performance and stability.

To assess the effect of ACHCl treatment on the performance of PSC devices, the complete devices with a p-i-n structure were fabricated. The trap density of states (tDOS) obtained from admittance spectroscopy of complete devices is illustrated in Fig. 4b, which can be utilized to analyze shallow and deep trap states of the devices [48]. The tDOS of the ACHCl-modified device is lower than that of the pristine device in the whole trap depth region. To quantitatively distinguish whether the defects suppressed by ACHCl treatment are predominantly shallow or deep trap states, we calculated the total decrease in defect density within different energy regions. Comparative analysis reveals that ACHCl treatment reduces the defect densities in the shallow-level region (Band 1, 0.30 ~ 0.40 eV) and the deep-level region (Band 2, 0.40 ~ 0.50 eV) by 1.44 × 10^17^ and 1.17 × 10^18^ cm^−3^, respectively. These results reveal that ACHCl treatment leads to a pronounced suppression of defects in the deep-level region, which is commonly associated with surface-related defects. This finding indicates that ACHCl treatment of perovskite surface effectively suppresses surface defects. These observations demonstrate that the reduction in total electrically active trap density observed in SCLC measurements is primarily driven by the passivation of deep trap states, rather than solely shallow traps. The dark-state impedance spectra of the devices shown in Fig. 4c, with their fitted electrical impedance presented in Table S6, reveal that the ACHCl-modified device exhibits a higher recombination resistance. This indicates that the introduction of ACHCl effectively reduces defects, thereby suppressing defect-assisted recombination in the device [22].

Based on the relationship between light intensity and the open-circuit voltage (*V*_*OC*_) and short-circuit current density (*J*_*SC*_) of the device, the extent of defect recombination within the device can be evaluated [24, 43]. From Fig. 4d, the ideality factor (n) for the ACHCl-modified device is 1.48, while that of the pristine device is 1.67. When only radiative recombination is considered, the value of n is equal to 1. The larger the n value, the more severe the non-radiative recombination in the device. Therefore, a smaller n value in the ACHCl-modified device l reflects a reduction in defects after surface treatment. The exponential factor (α) obtained from the *J*_*SC*_ versus light intensity curve (Fig. S34) indicates that the ACHCl-modified device has an α value closer to 1. This suggests that the introduction of ACHCl reduces interface recombination, prompting more carriers to be collected by the corresponding electrodes. Moreover, as shown in Figs. 4e and S35, transient photovoltage (TPV) and transient photocurrent (TPC) measurements reveal that the ACHCl-modified device exhibits slower photovoltage decay and faster photocurrent decay [45]. This suggests that the introduction of ACHCl effectively inhibits defect recombination within the device, thereby reducing the recombination rate of charge carriers and enhancing the carrier extraction efficiency.

The dark *J-V* curve of the complete device can be used to estimate current loss resulting from defect-assisted recombination [22]. From Fig. 4f, the dark current of the device with ACHCl is lower than that of the pristine device. This indicates that the surface treatment with ACHCl effectively reduces non-radiative recombination within the device, thereby preventing current leakage. The capacitance–voltage curves presented in Fig. S36 show that the ACHCl-modified device has a lower capacitance, which implies that the introduction of ACHCl effectively reduces charge accumulation at the interface. This reduction is advantageous for minimizing current losses in the device [50]. The characteristic curves of photocurrent versus effective bias, as shown in Fig. 4g, were obtained from *J-V* measurements under different conditions. At the same effective bias, the ACHCl-modified device clearly exhibits higher photocurrent compared to the pristine device, indicating an improvement in carrier collection efficiency. The Mott–Schottky curves of Fig. 4h can determine the ability to separate the photogenerated carriers within the devices [38]. The built-in potentials of the ACHCl-modified device and the pristine device can be determined to be 0.94 and 0.98 V, respectively. The enhanced built-in electric field due to the introduction of ACHCl indicates improved separation efficiency of holes and electrons, as well as an enhanced driving force for carriers to move toward the corresponding electrodes. The effective suppression of current loss and the enhanced carrier collection efficiency can likely be attributed to the introduction of ACHCl, which mitigates recombination caused by surface defects in the perovskite and induces surface polarization that leads to well-aligned energy levels. These positive effects are beneficial for improving the photovoltaic performance of the device.

### Device Performance and Stability

**Fig. 5:**
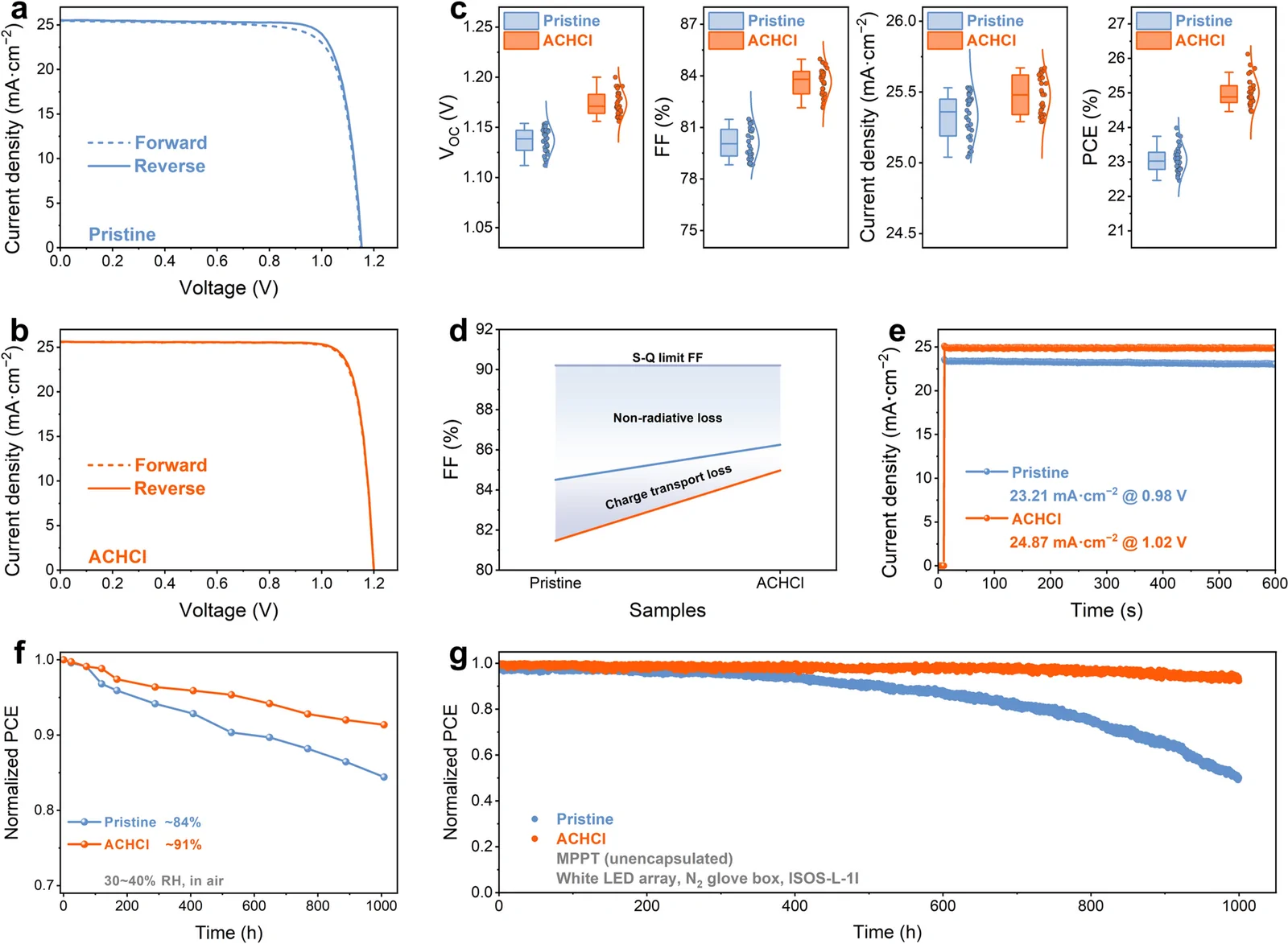
*J-V* curves of the **a** pristine and **b** ACHCl-treated devices. **c** Statistics distribution of photovoltaic parameters obtained from *J-V* measurements for pristine and ACHCl-treated devices. **d** FF loss analysis of pristine and ACHCl-treated devices. **e** Steady-state output current of pristine and ACHCl-treated devices at the voltage of the maximum power point. **f** Environmental stability during storage at 30% ~ 40% RH for 1000 h. **g** MPP stability tracking of unencapsulated devices following the ISOS-L-1I protocol

To evaluate the influence of the introduction of ACHCl on photovoltaic performance of the PSCs, *J-V* measurements were conducted under AM 1.5G illumination. Figure 5a, b shows the *J-V* curves under forward and reverse scan of the pristine device and the ACHCl-optimized device, with the specific photovoltaic parameters detailed in Table S7. From the reverse scan *J-V* measurements, the pristine device exhibits a PCE of 23.98%, a *V*_*OC*_ of 1.153 V, an FF of 81.47%, and a *J*_*SC*_ of 25.53 mA cm^−2^. On the other hand, the introduction of ACHCl significantly enhances the photovoltaic performance of the device, with the champion device achieving a PCE of 26.12%, a *V*_*OC*_ of 1.200 V, an FF of 84.97%, and a *J*_*SC*_ of 25.62 mA cm^−2^. Moreover, compared to the pristine device, the ACHCl-treated device exhibits a lower hysteresis factor, which can be attributed to the effective suppression of surface defects and the optimized energy-level alignment induced by surface polarization.

The incident photon-to-electron conversion efficiency (IPCE) spectra presented in Fig. S35 reveal that the integrated *J*_*SC*_ values for the ACHCl-treated device and the pristine device are 24.55 and 24.39 mA cm^−2^, respectively. The integrated *J*_*SC*_ values exhibit a discrepancy of approximately 5% compared to those obtained from the *J-V* curves, confirming the reliability of the *J*_*SC*_ values derived from the *J-V* measurements. To evaluate the reproducibility of the photovoltaic performance of PSC devices, a batch of 30 PSCs with p-i-n structure were fabricated and subjected to *J-V* measurements. Figure 5c and Table S8 illustrate the statistical distribution of photovoltaic parameters for ACHCl-treated device compared to the pristine devices. It is evident that the data distribution for ACHCl-treated device is more concentrated, and all photovoltaic parameters exhibit superior performance relative to those of the pristine devices. Notably, the enhancement in PCE primarily arises from increases in both *V*_*OC*_ and FF after surface treatment. These improvements are largely attributed to the effective passivation of surface defects by the carbonyl and chloride ions of the ACHCl, which reduces defect-assisted recombination at the interface. Additionally, the carbonyl groups of the ACH^+^ cations anchor onto Pb^2+^ defects and FA^+^ cations on the perovskite surface via coordination and hydrogen bonding, forming a relatively ordered cation-dipole layer that positively modulates the energy-level alignment and promotes efficient carrier separation and extraction. As illustrated in Fig. 5d and Table S9, the FF loss between the Shockley–Queisser limit and the actual FF is jointly influenced by non-radiative losses and charge transport losses [34]. In comparison with the pristine device, the ACHCl-modified device shows smaller non-radiative recombination losses and charge transfer losses. When the effect of charge transfer loss is neglected, the FF_max_ of the pristine device is 84.51%, which is lower than the 86.25% FF_max_ of the ACHCl-modified device.

Despite improvements in the photovoltaic performance of the devices, their stability remains a critical issue that requires further investigation. To investigate the photostability of the devices, the steady-state output tests (Figs. 5e and S38) were conducted on different devices. Under standard sunlight irradiation, the steady-state output current of the pristine device exhibited a significant downward trend after 10 min, while the ACHCl-modified device maintained a relatively stable output, demonstrating markedly superior photostability compared to the pristine device. The average steady-state PCE of the ACHCl-modified device is 25.37%, whereas the pristine device exhibits an average steady-state PCE of only 22.74%. The environmental stability of the devices was also monitored under conditions of approximately 25 °C and 35% relative humidity (RH), as shown in Fig. 5f. After 30 days of monitoring environmental stability, the PCE of the pristine device remained at only 84% of its initial efficiency, whereas the ACHCl-treated device retained 91% of its initial efficiency. After thermal stability test at 85 °C for 120 h, the high-temperature stability of ACHCl-modified device in air can reach 81% of the initial efficiency, while the pristine device decreased to 69% of the initial efficiency, as shown in Fig. S39.

Furthermore, the operational stability of the unencapsulated device under continuous illumination was further evaluated by conducting maximum power point tracking (MPPT) tests using a white LED array in a nitrogen glove box, in accordance with the ISOS-L-1I protocol. In Fig. 5g, the ACHCl-modified device maintained 92% of its initial efficiency after 1000 h, outperforming the pristine device, which retained only 50% of its initial PCE. To strengthen the reliability of the stability assessment, device stability under ultraviolet (UV) illumination was further evaluated. The unencapsulated devices were continuously exposed to 365 nm UV light (20 mW cm^−2^) in air at 25 °C and a relative humidity of approximately 30%. As shown in Fig. S40, the ACHCl-treated device retained approximately 72% of its initial power conversion efficiency after 120 h of continuous UV irradiation, whereas the unencapsulated pristine device retained only about 46% under the same conditions. This result demonstrates enhanced resistance to UV-induced degradation following ACHCl treatment. This improvement can be attributed primarily to the effective reduction in perovskite defect density and stabilization of the perovskite lattice induced by ACHCl, which together suppress UV-accelerated degradation pathways.

The enhancement in device stability can be attributed to the introduction of ACHCl, which reduces the perovskite surface defects that lead to perovskite degradation. In order to investigate the degradation of perovskite films, XRD technique was used to detect ACHCl-modified perovskite and pristine perovskite film under the condition of about 60% RH. From Fig. S41, it can be noticed that after 100 h, the diffraction peak of the (100) crystalline plane of the pristine perovskite films decreased and the diffraction peaks of the δ-phase FAPbI_3_ and PbI_2_ began to appear [22]. After 200 h, more severe phase separation and degradation occurred in the pristine perovskite films. In contrast, the ACHCl-modified perovskite film retained a stable α-phase after 200 h under the same storage conditions, which proved that the introduction of ACHCl effectively inhibited the degradation of the perovskite film. Furthermore, the hydrophobic cyclohexyl groups in ACHCl-modified film can also act as a barrier against moisture intrusion, serving to prevent the degradation of perovskite films. As shown in Fig. S42, the ACHCl-modified perovskite film obviously has a larger water contact angle than the pristine perovskite film, which implies that the introduction of ACHCl enhances the ability of the perovskite film to resist water erosion. Benefiting from the reduced perovskite surface defects and enhanced moisture resistance, the stability of ACHCl-modified devices is superior to that of pristine devices.

## Conclusions

In summary, we successfully introduced dipole molecule ACHCl into the interface between the perovskite layer and the electron transport layer in PSCs with p-i-n structure, effectively modulating the interfacial properties of the perovskite film, thereby achieving high-performance. Based on the experimental investigation, the functional terminals of the negatively charge carbonyl group on ACHCl can not only effectively reduce the uncoordinated Pb^2+^ defects and inhibit defect-assisted recombination but also can anchor on the perovskite surface to form a relatively ordered cation-dipole layer that induce surface polarization of the perovskite film, thus minimizing energy loss at the PVK/ETL interface. This surface modification effectively reduces interfacial defects and optimizes the interfacial energy-level arrangement, resulting in a champion PCE of 26.12% for the ACHCl-treated PSC device. Additionally, the optimized devices exhibit excellent stability. This work provides insight into the surface polarization induced by dipoles in perovskite films for achieving efficient and stable PSCs.

## Supplementary Information

Below is the link to the electronic supplementary material.

Supplementary file1 (DOCX 40703 KB)
