# Supplementary material for: Defect-Anchored Dipole Molecules Induce Surface Polarization Facilitating High-Performance Inverted Perovskite Solar Cells
Source: Nanomicro Lett. 2026 Mar 18;18:289. doi: 10.1007/s40820-026-02150-7 (PMC12996491; doi:10.1007/s40820-026-02150-7)
Supplement: Supplementary file 1 — Supplementary file1 (DOCX 40703 KB) [file 40820_2026_2150_MOESM1_ESM.docx]

Supporting Information for

**Defect-Anchored Dipole Molecules Induce Surface Polarization Facilitating High-Performance Inverted Perovskite Solar Cells**

Weichun Pan^1^, Jihuai Wu^1,^ *, Jiexi Pan^1^, Shanyue Wei^1^, Lina Tan^1^, Wenjing Li^1^, Deng Wang^2^, Xuping Liu^2^, Yiming Xie^1^, Jianming Lin^1^, Zhang Lan^1,^ *

^1^ Engineering Research Center of Environment-Friendly Functional Materials, Ministry of Education, Fujian Key Laboratory of Photoelectric Functional Materials, College of Materials Science and Engineering, Huaqiao University, Xiamen, 361021, P. R. China

^2^ Key Laboratory of Environmentally Friendly Functional Materials and Devices, Lingnan Normal University, Zhanjiang, 524048, P. R. China

*Corresponding authors. E-mail: [jhwu@hqu.edu.cn](mailto:jhwu@hqu.edu.cn) (Jihuai Wu); [lanzhang@hqu.edu.cn](mailto:lanzhang@hqu.edu.cn) (Zhang Lan)

**S1 Supplementary Methods**

**S1.1 Film Characterization**

The surface morphology of the samples was examined using SEM (SU-8000, Hitachi) and AFM (Multimode-8j, Bruker). Crystallographic properties were characterized by XRD with Cu-Kα radiation (SmartLab, Rigaku). Intermolecular interactions were further probed using nuclear magnetic resonance (AVANCE III 500 MHz, Bruker). Changes in atomic states and surface energy-level structures were analyzed using photoelectron spectroscopy (K-Alpha+, ThermoFisher). The UPS spectra provided the electron onset energy (E_onset_) and the electron cutoff energy (E_cutoff_) of the samples. From these measurements, the work function (W_F_), valence band maximum (VBM), and conduction band minimum (CBM) were determined using the following equation [S1]:

$$W_{F}=hv-E_{\mathrm{cutoff}}$$

$$E_{\mathrm{VBM}}=-W_{F}-E_{\mathrm{onset}}$$

$$E_{\mathrm{CBM}}=E_{\mathrm{VBM}}+E_{g}$$

where *hv* denotes the incident photon energy (21.22 eV) and E_g_ represents the bandgap of the material. UV-Vis absorption spectra were recorded using a Lamda 950UV/VIS/NIR spectrometer (PerkinElmer). The Urbach energy (*E_U_*) is a parameter that describes the strength of the interaction between the energy band structure of solid materials and photons. The structure and defects of the material can affect the magnitude of *E_U_*. The relationship between Urbach energy (*E_U_*) and the absorption coefficient (*α*) is described by the following formula [S2]:

$$\alpha=\alpha_{0}\exp\frac{hv}{E_{U}}$$

where *α_0_* is a material-specific constant. SSPL measurements were performed using a highly sensitive fluorescence spectrophotometer (LF-1303003, Thermo Scientific). TRPL decays were recorded using a single-photon counting system (Pico harp 300) coupled with an Omin-k Monochromator/Splitter (Zolix). TRPL decay curves were analyzed by fitting to a bi-exponential model, expressed as [S3]:

$$f\left( t \right)=A_{1}e^{\frac{-t}{\tau_{1}}}+A_{2}e^{\frac{-t}{\tau_{2}}}+B$$

where *τ_1_* and *τ_2_* represent the fast and slow decay lifetimes, respectively. A_1_ and A_2_ are the corresponding amplitudes. The average carrier lifetime (*τ_avg_*) was determined using the following equation [S4]:

$$\tau_{avg}=\frac{{{A_{1}\tau}_{1}}^{2}+{{A_{2}\tau}_{2}}^{2}}{{A_{1}\tau}_{1}+{A_{2}\tau}_{2}}$$

The relationship between the photogenerated charge carrier density (*n_c_*) and integrated PL intensity (*I_PL_*) was conformed using the following equation [S5]:

$$I_{PL}=k\int_{0}^{\infty} \frac{n_{c}\left( t \right)}{\tau}\mathrm{dt}$$

where *τ* is the PL lifetime, *t* is the time, and *k* is a sample-dependent constant. Differential lifetime simulation of TRPL data were performed according to the following equation [S6]:

$$\tau_{PL}=\left( -\frac{1}{m}\frac{d\ln\left( \varphi_{PL} \right)}{dt} \right)^{-1}$$

where *φ_PL_(t)* is the PL intensity at time *t* following pulse excitation, and the *m* is a factor related to the charge injection level.

**S1.2 Device Characterization**

The J-V characteristics of PSCs were measured using a Keithley 2420 source meter under AM 1.5G illumination provided by a solar simulator (Oriel Sol 3A, Newport). The light intensity was calibrated using a Si reference solar cell certified by NREL. Corresponding dark J-V curves were obtained under identical conditions in the absence of illumination. The maximum power point tracking (MPPT) measurements were performed using a commercial MPPT system (YH-VMPP-IV-16, Suzhou Yanghua Electronic Equipment). The MPPT testing fixture was placed inside an N_2_-filled glovebox and cooled by forced-air circulation. Under continuous operation, the device temperature was maintained at approximately 25°C, corresponding to the ISOS-L-1I protocol for light-soaking stability measurements. In addition, the white LED light source itself was equipped with combined water and air cooling to minimize thermal fluctuations during long-term testing. The LED array comprises 16 individual LEDs with different peak wavelengths, producing a composite spectral range of approximately 400-800 nm, which adequately covers the strong absorption region of the perovskite absorber (bandgap of 1.55 eV). Electron-only devices were used to evaluate electron trap densities via the space-charge-limited current (SCLC) method. Once the trap-filled limit voltage (*V_TFL_*) was determined, the trap density (*N_trap_*) was calculated as follows [S7]:

$$N_{trap}=\frac{2\varepsilon\varepsilon_{0}V_{TFL}}{eL^{2}}$$

where *e* is the elementary charge, *L* is the perovskite layer thickness, *ε* is the relative permittivity of the perovskite, and *ε_0_* is the vacuum permittivity. Light-intensity-dependent open-circuit voltage (*V_OC_*) measurements were performed under various illumination levels (*I*), following the relation [S8]:

$$V_{oc}=\frac{nKT}{e}\ln\left( I \right)+constant$$

where *n* is the ideality factor, *K* is the Boltzmann constant, and *T* is the absolute temperature. TPV, TPC, C-V, M-S, EIS, and tDOS measurements were carried out using an electrochemical workstation (IM6e, Zahner). The built-in electric potential (*V_bi_*) was determined from M-S measurements to evaluate charge extraction efficiency, following [S9]:

$$\frac{1}{C^{2}}=\frac{2}{\varepsilon\varepsilon_{0}{eN}_{D}S^{2}}\left( V_{bi}-V \right)$$

where *S* is the device active area and *N_D_* is the doping density. To obtain the tDOS curves, the angular frequency-dependent capacitance was used, following the formula [S10]:

$$N_{t}\left( E_{\omega} \right)=-\frac{\omega}{KT}\frac{V_{bi}}{eW}\frac{dC}{d\omega}$$

where *E_ω_* is the demarcation energy, *N_t_* is the trap density, *ω* is the angular frequency, and *W* is the depletion width. The corresponding *E_ω_* can be determined by the equation [S10]:

$$E_{\omega}=KT\ln\left( \frac{\omega_{0}}{\omega} \right)$$

where *ω_0_* signifies the attempt-to-escape frequency. To quantify hysteresis in the devices, the hysteresis index (HI) can be calculated using the formula [S1]:

$$HI=\frac{\mathrm{PCE}_{\mathrm{reverse}}-\mathrm{PCE}_{\mathrm{forward}}}{\mathrm{PCE}_{\mathrm{reverse}}}$$

where PCE_reverse_ and PCE_forward_ are the power conversion efficiencies derived from J-V curves measured in reverse and forward scanning directions, respectively. The loss in the measured FF from the Shockley-Queisser limit is attributed to charge transport and non-radiative losses. The maximum fill factor (FF_max_) without charge transport loss can be calculated using the following equation [S11]:

$$\mathrm{FF}_{\max}=\frac{v_{\mathrm{oc}}-\ln(v_{\mathrm{oc}}+0.72)}{v_{\mathrm{oc}}+1}$$

$$v_{\mathrm{oc}}=\frac{eV_{\mathrm{OC}}}{nkT}$$

The quasi-Fermi level splitting (QFLS) of samples with different structural configurations deposited on glass substrates was determined through photoluminescence quantum yield (PLQY) measurements. The direct relationship between QFLS and PLQY can be expressed as follows [S12]:

$$QFLS=KT\cdot ln\left( PLQY\frac{J_{G}}{J_{0,rad}} \right)$$

where, QFLS is the difference between the electron and hole quasi-Fermi levels in the perovskite layer, *J_G_* is the generation current density under illumination, and *J_0,rad_* is the dark radiative recombination saturation current density. *J_0,rad_* can be calculated from the following equations [S12]:

$$J_{0,rad}=e\int_{0}^{\infty} {EQE}_{PV}(E)\Phi_{BB}(E)ⅆE$$

$$\Phi_{BB}\left( E \right)=\frac{2\pi E^{2}}{h^{3}c^{2}}\cdot\frac{1}{exp\left( \frac{E}{KT} \right)-1}$$

where *EQE_PV_* is the photovoltaic external quantum efficiency, *E* is the photon energy, *Φ_BB_* is the black-body radiative spectrum, *h* is the Planck constant, and *c* is the light speed in a vacuum. Based on the above equation, the *J_0,rad_* was calculated similarly 7.52×10^−20^ mA∙cm^−2^. Besides, the V_OC_ non-radiation recombination loss ${\Delta V}_{OC}^{non-rad}$ can be obtained according to the following equation [S12]:

$${\Delta V}_{OC}^{non-rad}=\frac{KTln\left( PLQY \right)}{e}$$

**S1.3 Computational details**

All electronic structure calculations were carried out using the CP2K package [S13] employing the Quickstep module within the Gaussian and plane-wave (GPW) formalism. The open-source code Multiwfn [S14] was used to generate input files and analyze electronic structures. Geometry optimizations were performed with the B3LYP hybrid functional including Grimme’s D3 dispersion correction. The TZVP-MOLOPT-GTH basis sets were applied for C, H, O, and N atoms, while Pb and I atoms were described using the corresponding MOLOPT basis sets combined with Goedecker-Teter-Hutter (GTH-PBE) pseudopotentials. The Broyden-Fletcher-Goldfarb-Shanno (BFGS) algorithm was used for structural relaxation until the convergence thresholds for energy and force were satisfied. Frequency analyses confirmed that all optimized geometries correspond to true minima with no imaginary frequencies. The molecular geometry of CHD, ACHA, and ACH^+^ were optimized in the gas phase to obtain their ground-state structures, which were subsequently used for electronic structure and interaction analyses. The structural optimizations of ACH^+^ cation interacting with PbI_2_ and FAI were performed in the implicit solvent environment of isopropanol, introduced through the self-consistent reaction field (SCRF) module implemented in CP2K. The electrostatic potential (ESP) [S15] distributions and interaction region indicator (IRI) [S16] analysis were carried out using Multiwfn, and molecular visualizations were produced using VMD [S17].

First-principles density functional theory (DFT) calculations were further performed using CP2K to investigate the adsorption behavior of the ACH^+^ cation on the (001) surfaces of FAPbI_3_ perovskite. To elucidate the anchoring behavior and preferred orientation of ACH^+^ on the perovskite surface, three distinct adsorption configurations were constructed, in which the six-membered ring plane formed angles of 0°, 45°, and 90° relative to the PbI_2_-terminated surface. In addition, the adsorption behavior of the ACH^+^ cation on the FAI-terminated and mixed-terminated perovskite surfaces was also analyzed. The Perdew-Burke-Ernzerhof (PBE) functional with Grimme’ D3 dispersion correction was used to account for long-range van der Waals interactions. The GPW hybrid basis scheme employed DZVP-MOLOPT basis set in combination with GTH pseudopotentials, and a plane-wave cutoff energy of 400 Ry was applied for the auxiliary density. A vacuum spacing of 20 Å was introduced to eliminate interlayer interactions. The Brillouin zone was sampled with a Γ-centered 2×2×1 Monkhorst-Pack grid. All atomic positions were relaxed until the total energy and residual forces converged below 1×10^−5^ eV and 0.02 eV/Å, respectively. The adsorption energy (*E*_A_) was calculated using the expression:

$$E_{A}=E_{slab+molecule}-E_{\mathrm{slab}}-E_{\mathrm{molecule}}$$

where *E*_slab+molecule_ is the total energy of the adsorbed system, *E*_slab_ is the energy of the relaxed defective slab, and *E*_molecule_ is that of the isolated molecule. A more negative *E*_A_ indicates stronger adsorption.

S2 Supplementary Figures


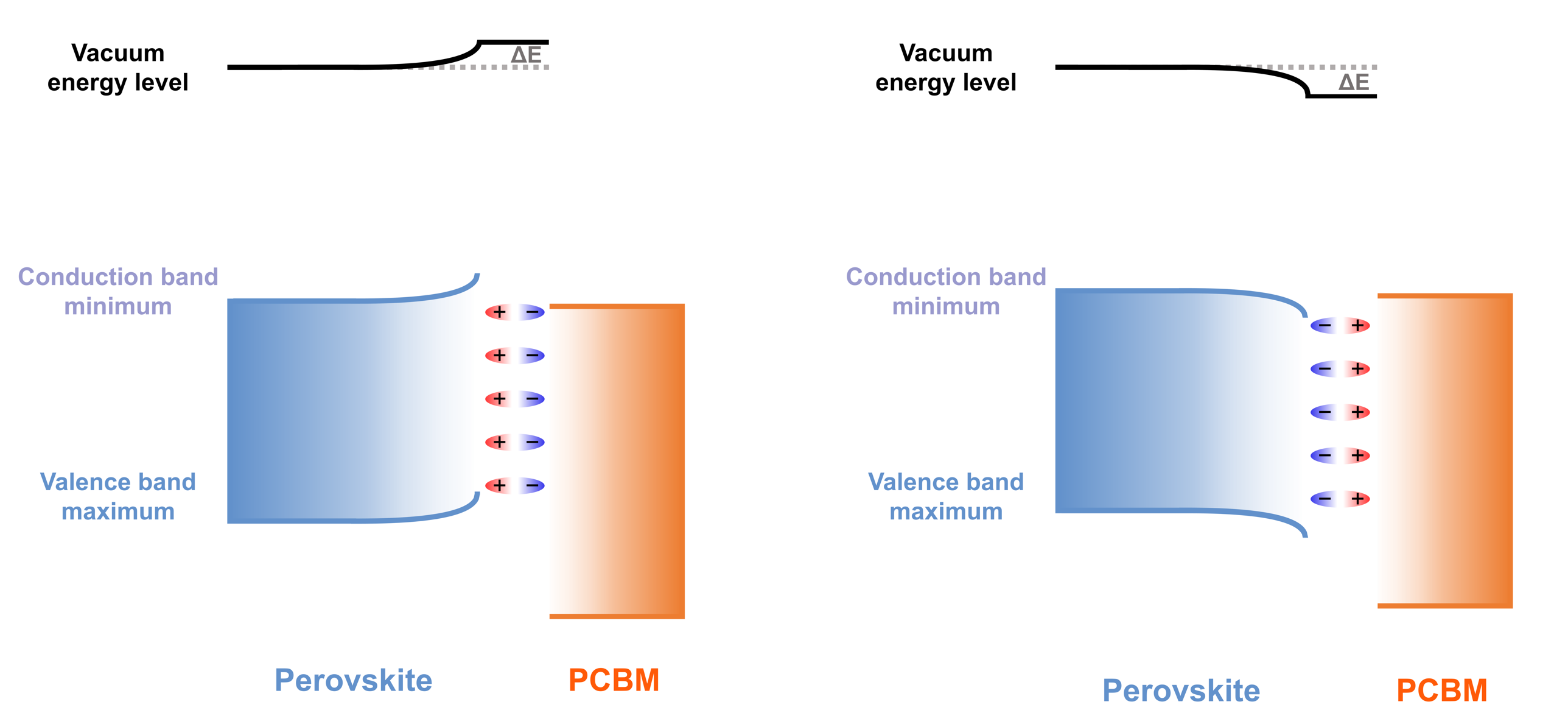


**Fig. S1** Schematic of the energy bands at the PVK/ETL interface based on the dipole effect


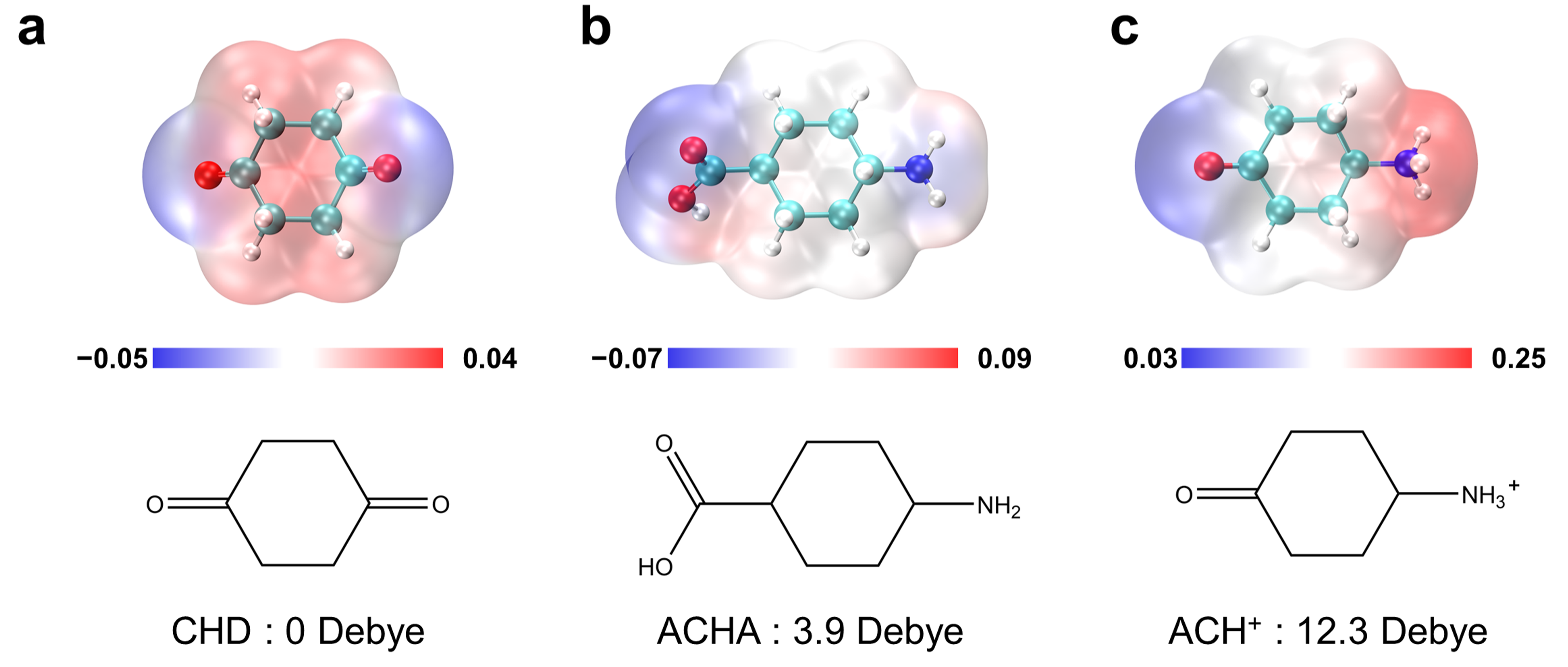


Fig. S2 ESP images and molecular structures of CHD, ACHA, and ACH^+^ cation


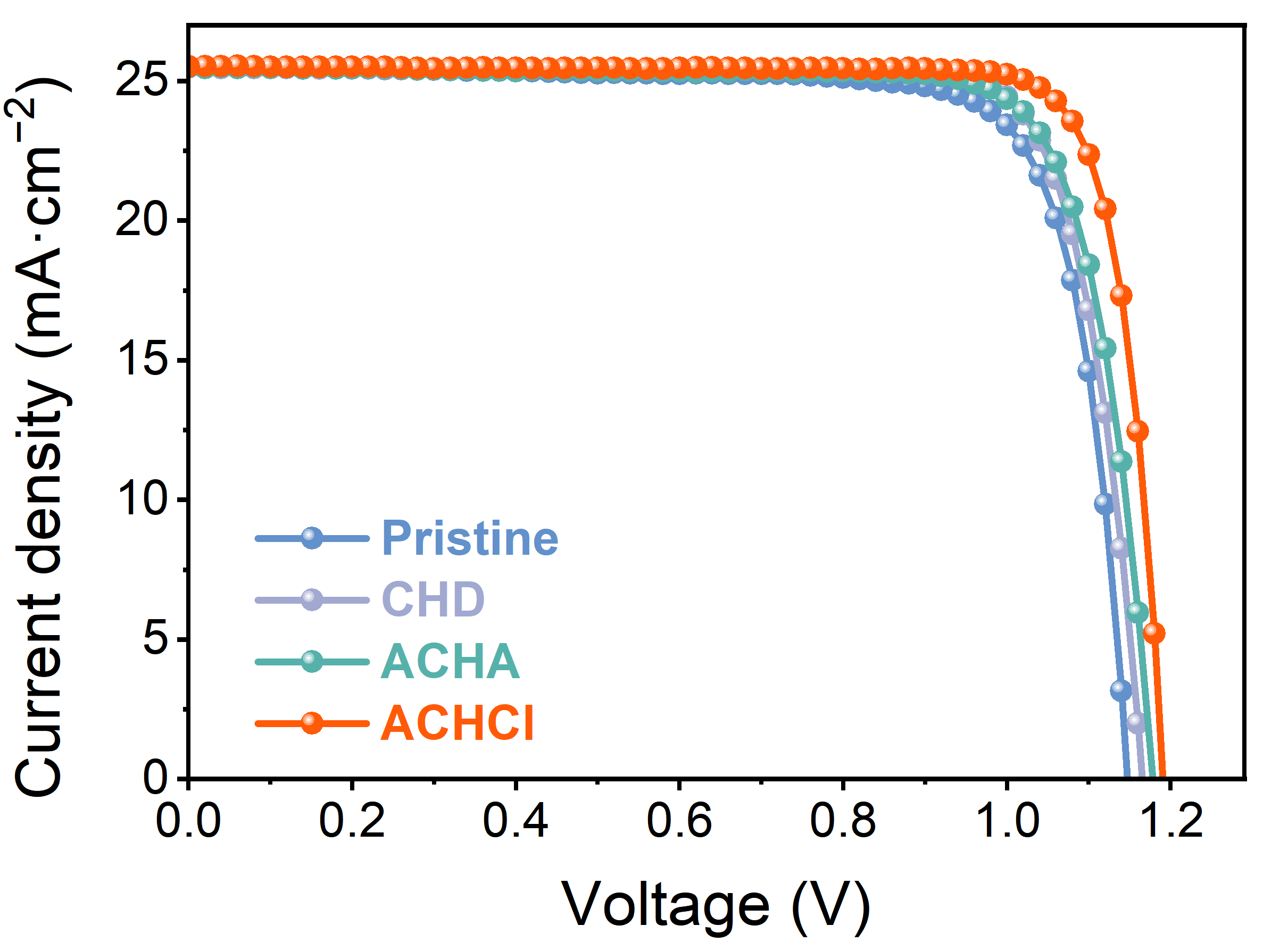


Fig. S3 The *J-V* curves of the devices treated with different molecules


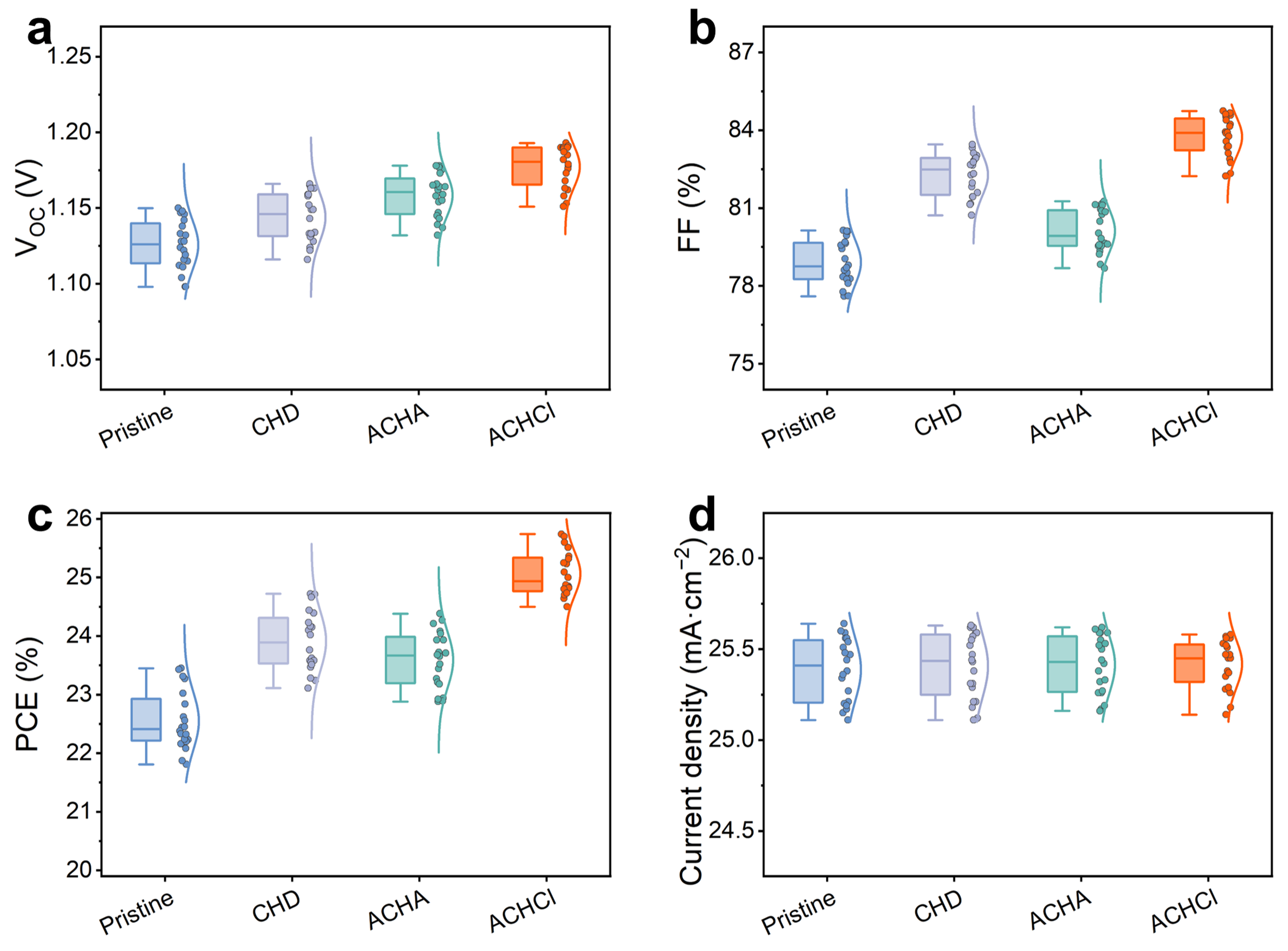


Fig. S4 The statistics distribution of photovoltaic parameters obtained from J-V measurements for the devices treated with different molecules


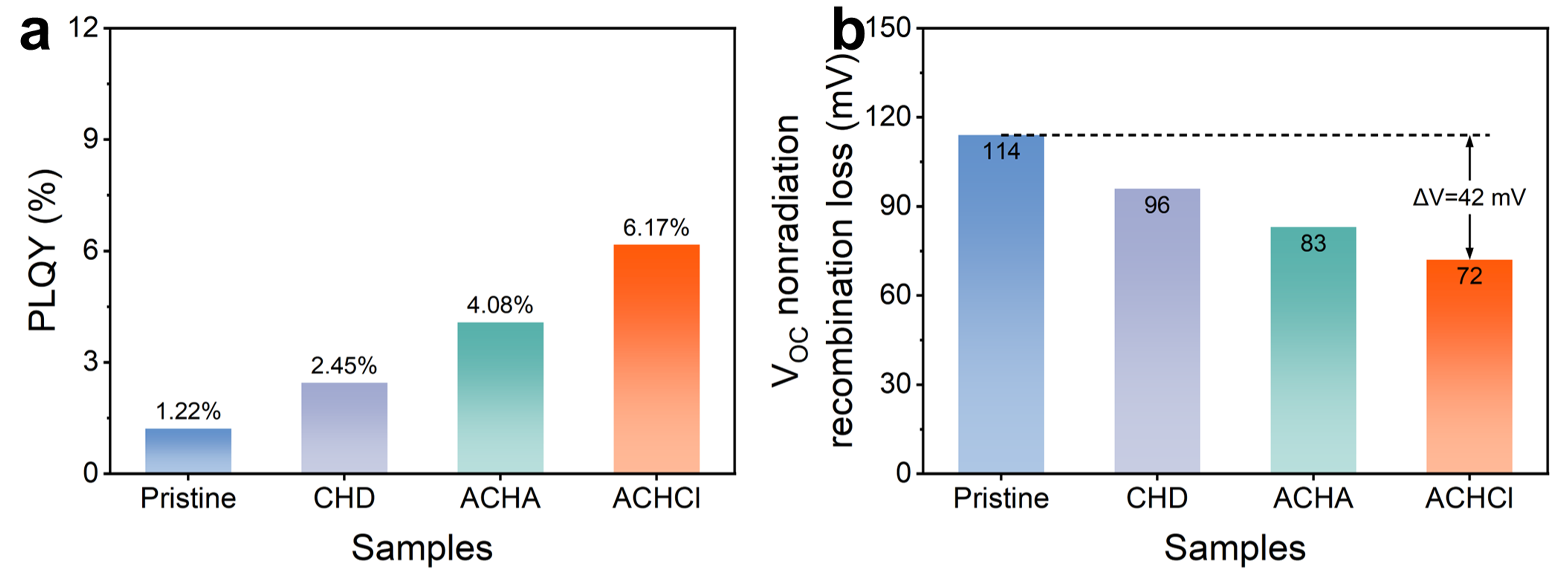


Fig. S5 (a) PLQY values of different samples based on the Glass/PVK/ETL structure. (b) The calculated V_OC_ non-radiation recombination loss values of different samples based on the Glass/PVK/ETL structure


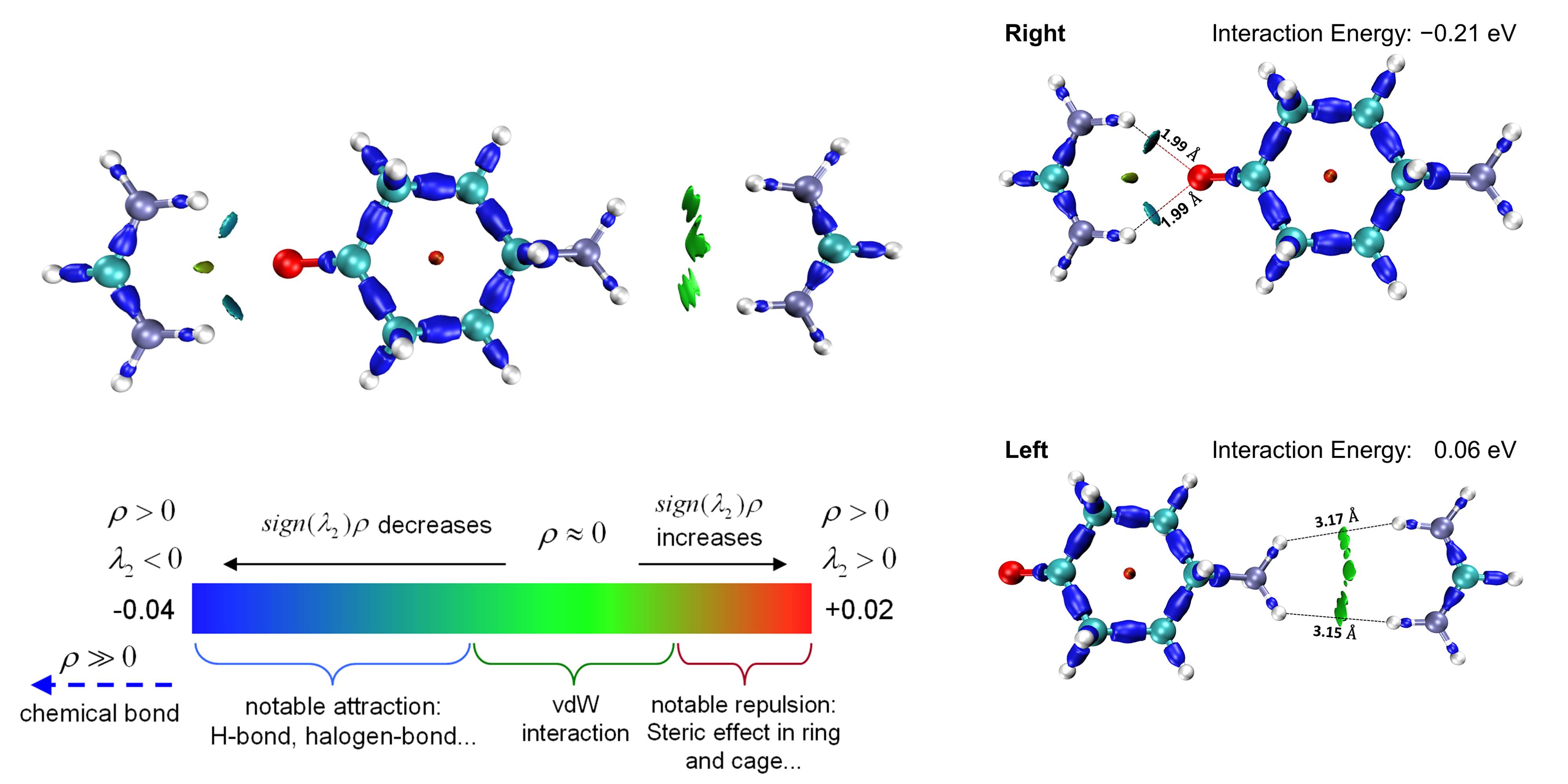


**Fig. S6** The IRI images of the interactions between the ACH^+^ cation and FA^+^ cations. The structures of the ACH^+^-FA^+^ bimolecular models were further optimized to elucidate the respective contributions of the two functional groups in ACH^+^ to their interactions with FA^+^. The resulting interatomic distances and interaction energies were annotated


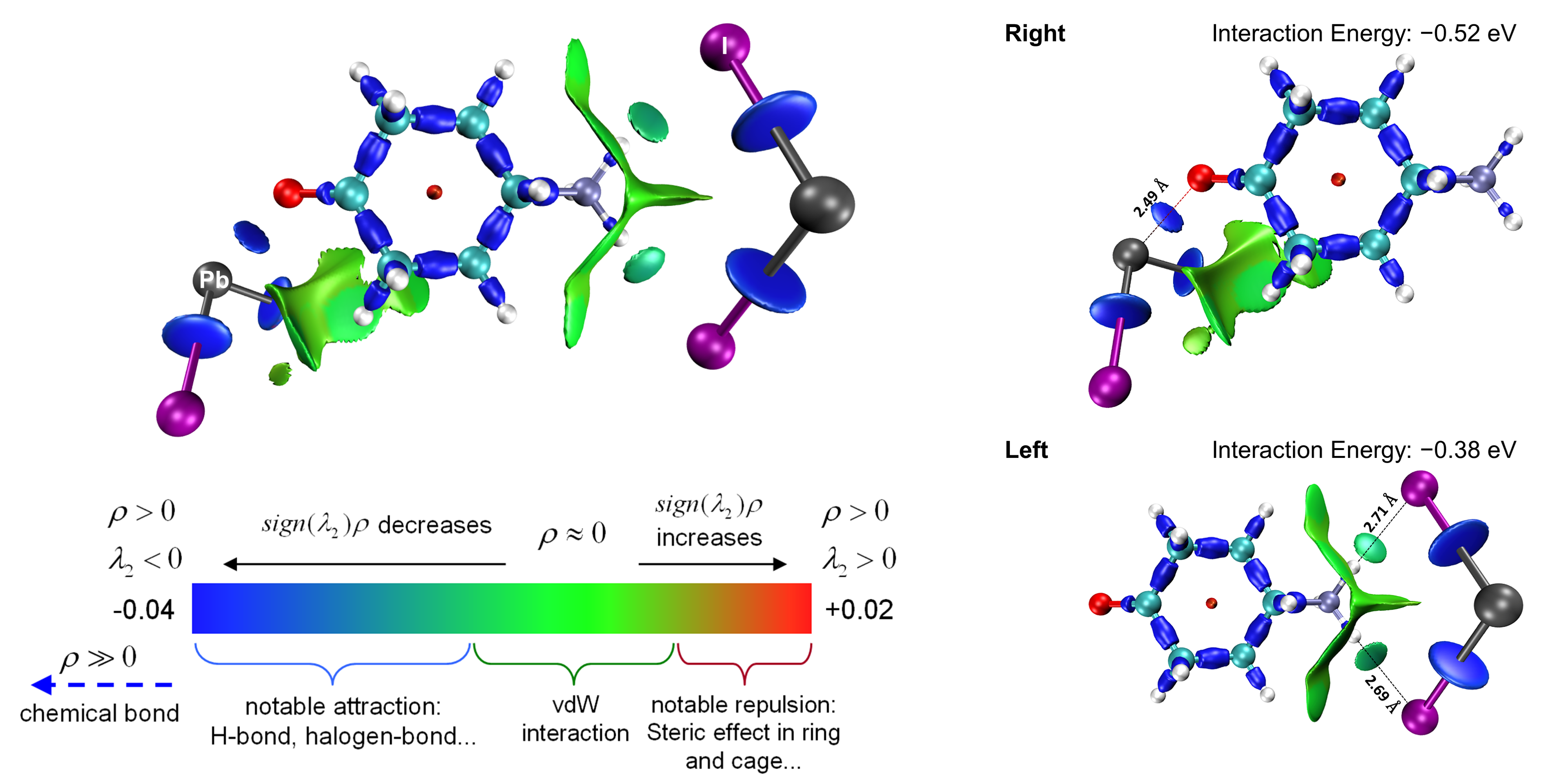


**Fig. S7** The IRI images of the interactions between the ACH^+^ cation and PbI_2_. The structures of the ACH^+^-PbI_2_ bimolecular models were further optimized to elucidate the respective contributions of the two functional groups in ACH^+^ to their interactions with PbI_2_. The resulting interatomic distances and interaction energies were annotated


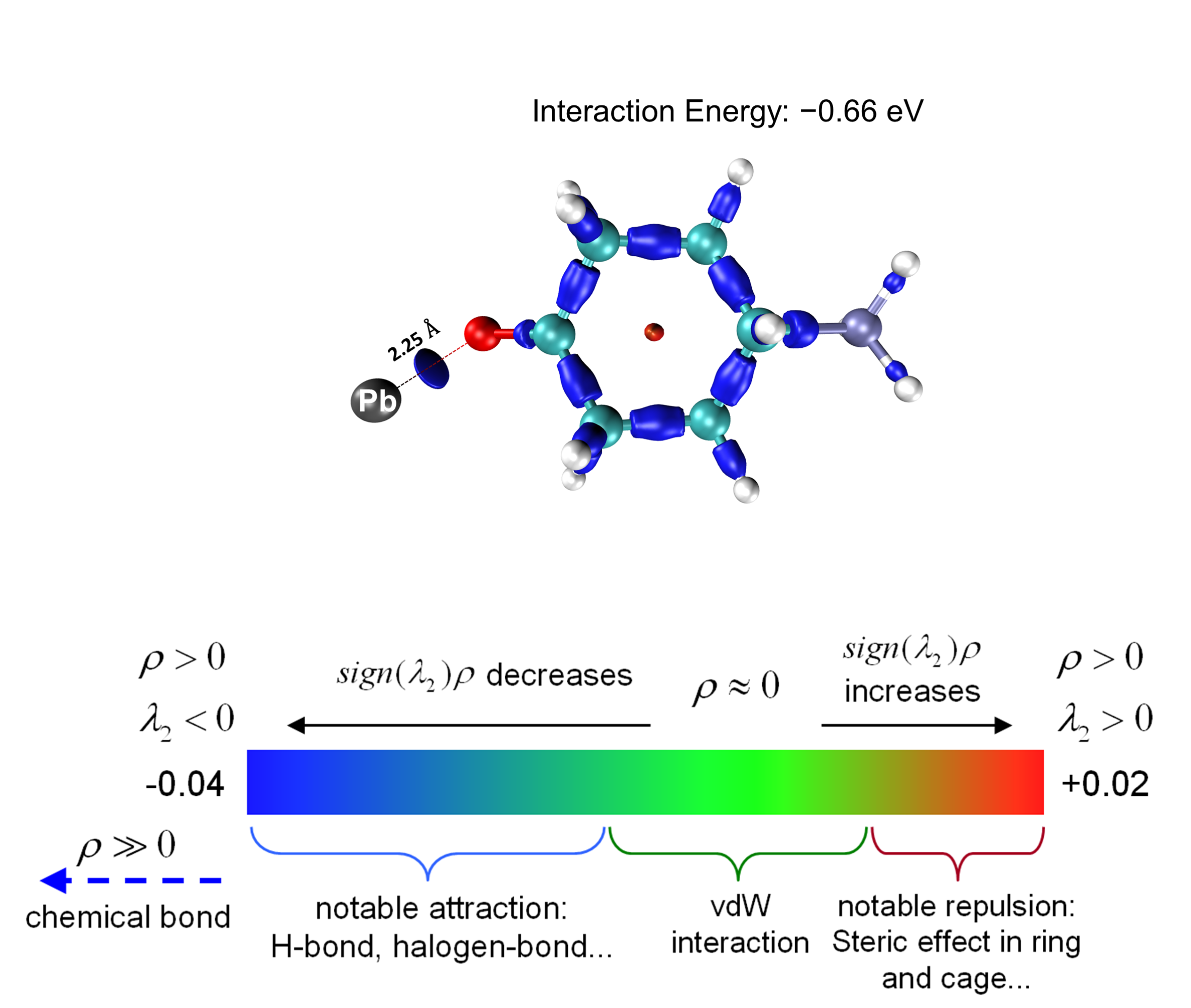


**Fig. S8** The IRI image of the interactions between the ACH^+^ cation and Pb^2+^ ion. The resulting interatomic distances and interaction energies were annotated


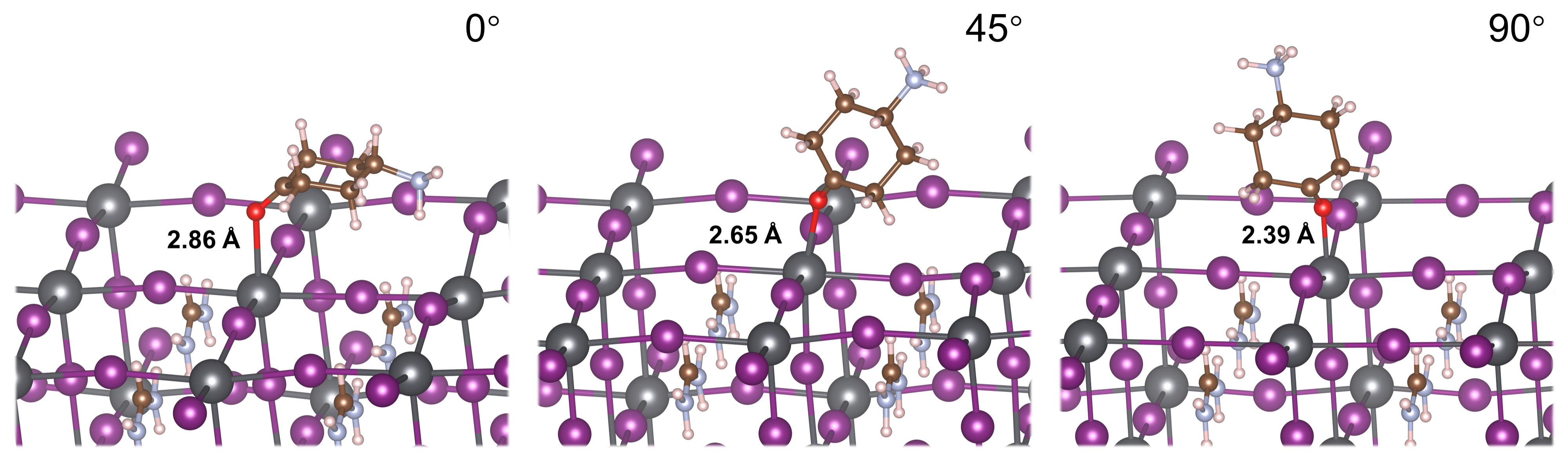


Fig. S9 Bond lengths of the Pb−O bonds corresponding to the ACH^+^ cation adsorbed on the perovskite surface at different orientations

**
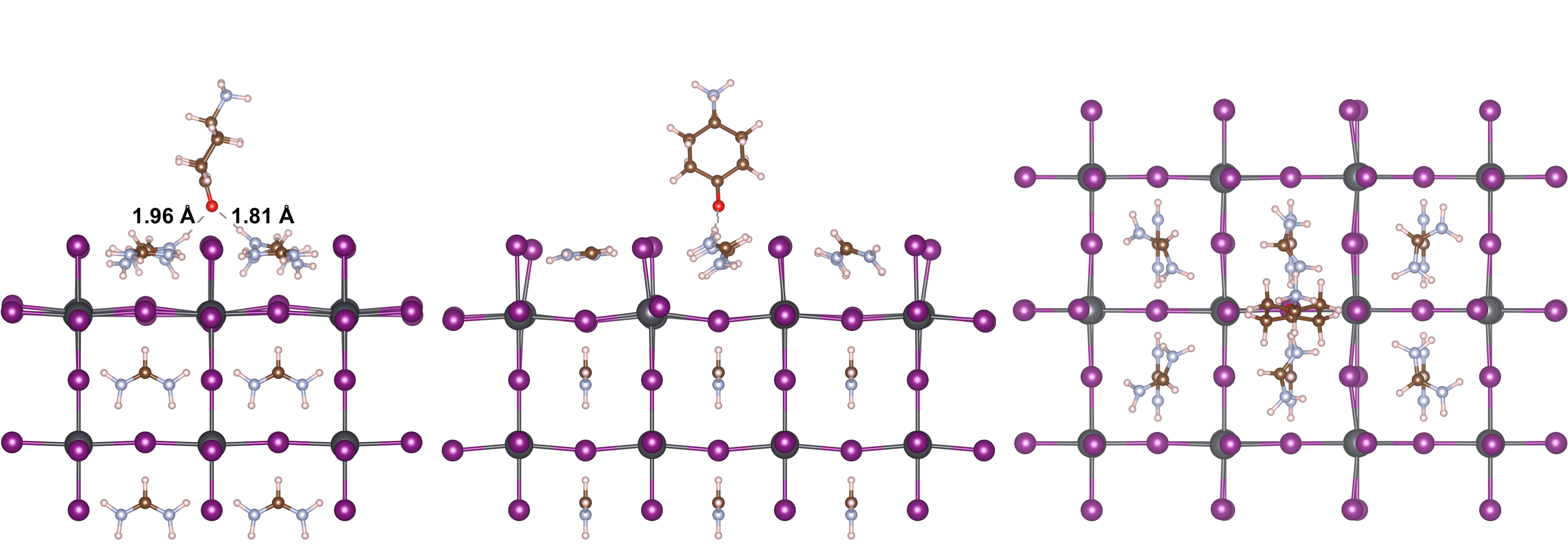
**

**Fig. S10** Theoretical model of the ACH^+^ cation adsorbed on the FAI-terminated perovskite surface

**
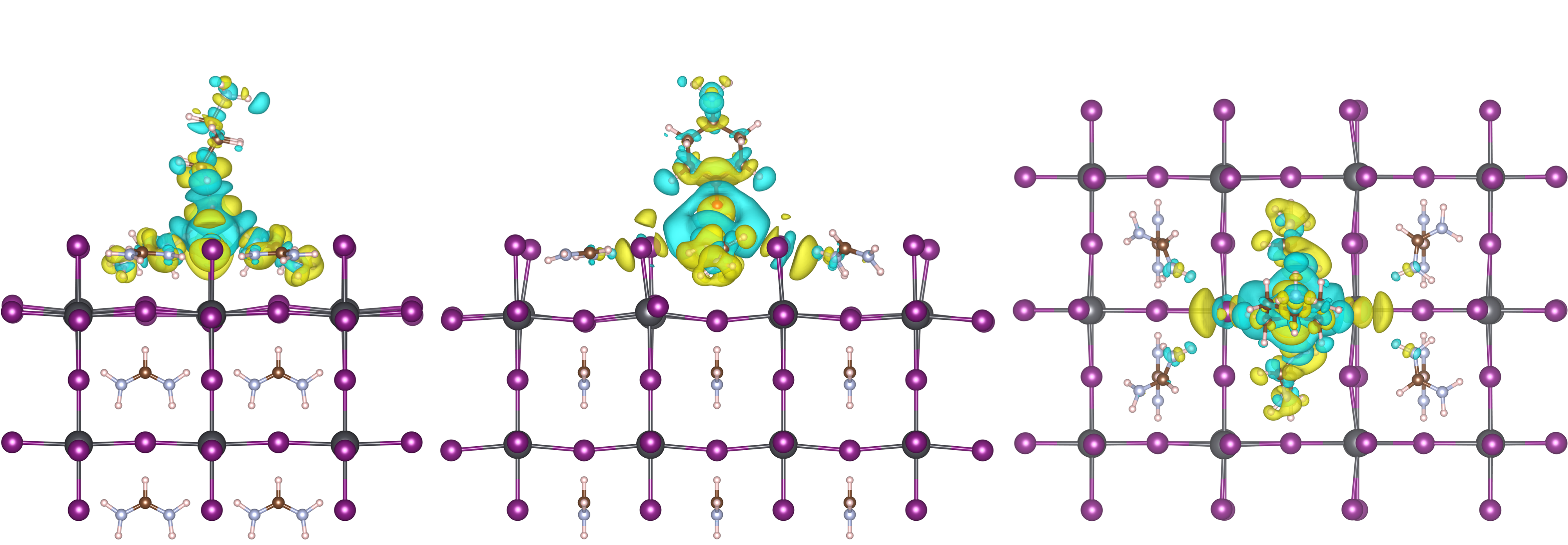
**

**Fig. S11** Differential charge density for the theoretical model of ACH^+^ cation adsorbed on the FAI-terminated perovskite surface

**
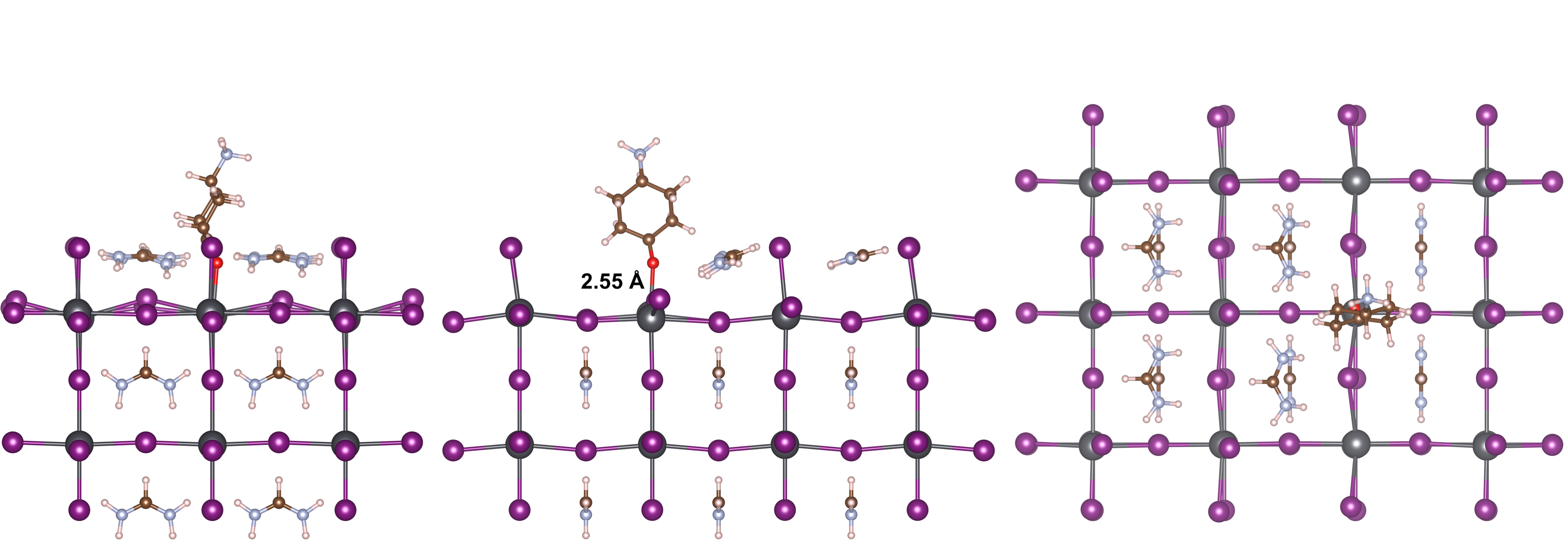
**

**Fig. S12** Theoretical model of the ACH^+^ cation adsorbed on the mixed-terminated perovskite surface


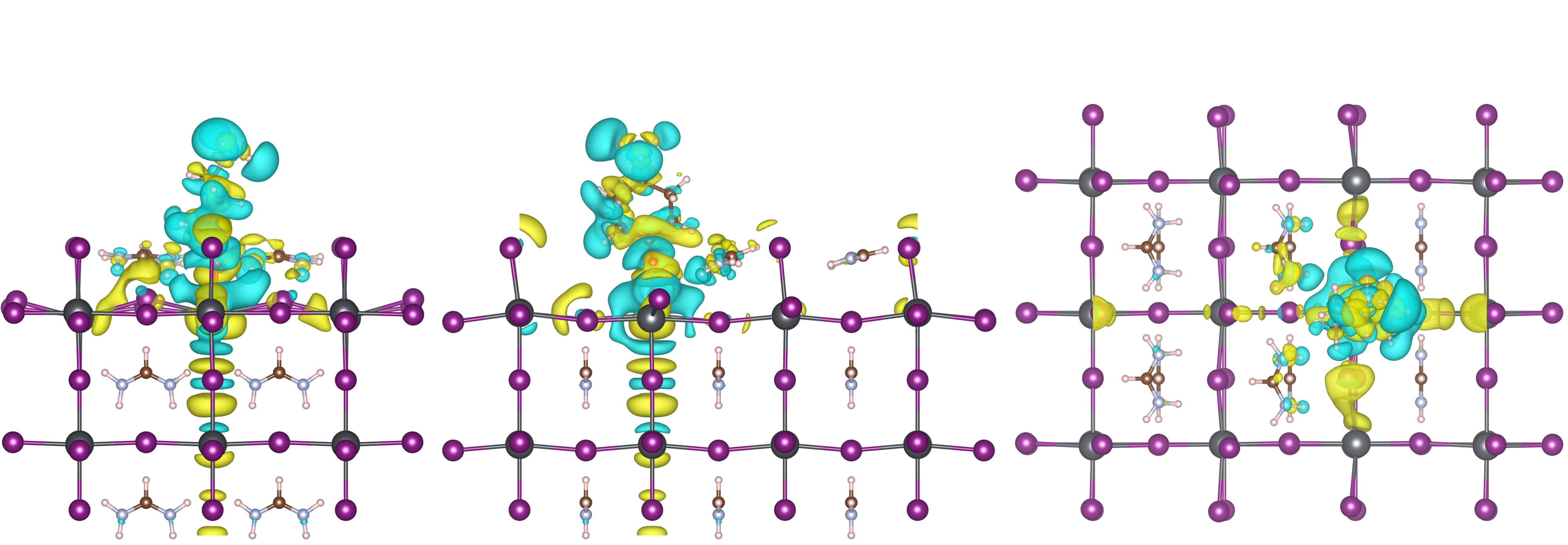


**Fig. S13** Differential charge density for the theoretical model of ACH^+^ cation adsorbed on the mixed-terminated perovskite surface


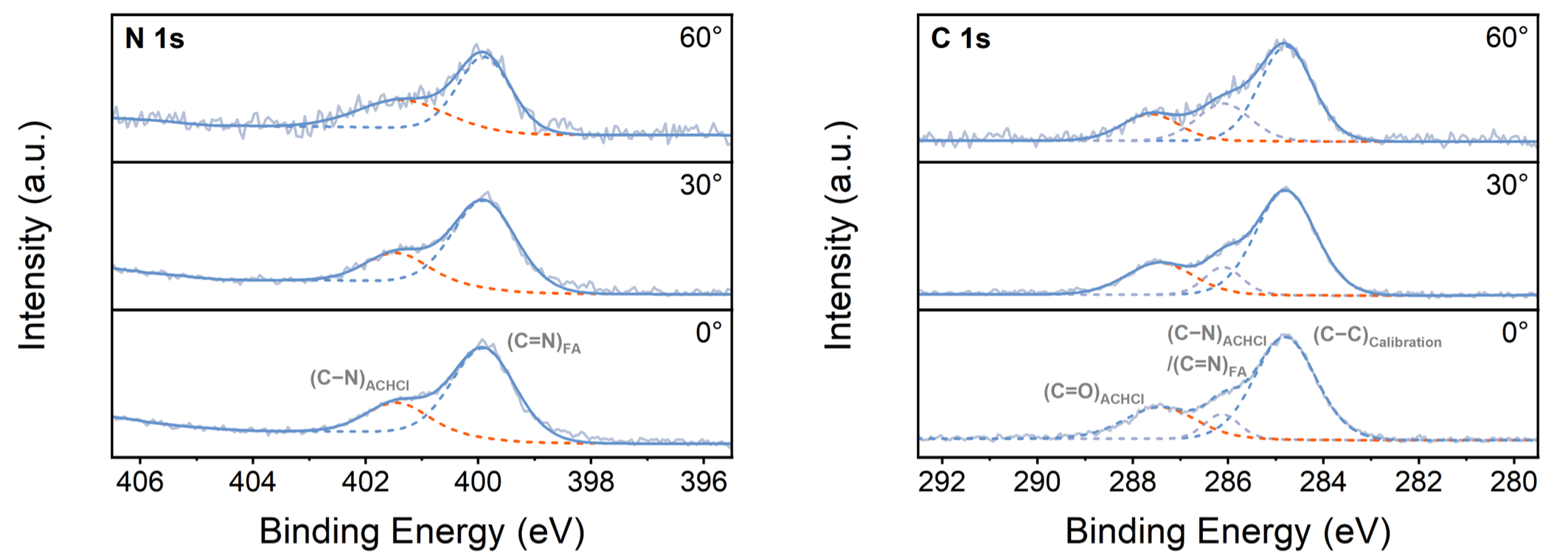


Fig. S14 AR-XPS N 1s and C 1s spectra of the perovskite film as a function of electron take-off angles of 0°, 30°, and 60°


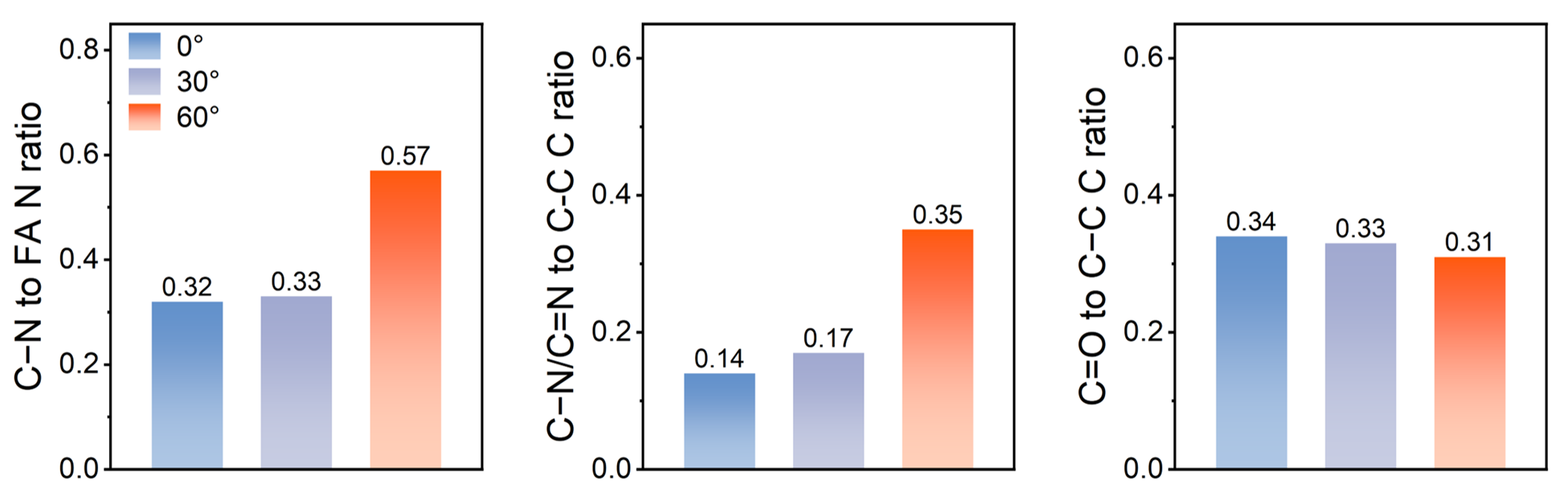


Fig. S15 C−N to FA N ratios, C−N/C=N to C−C C ratios, and C=O to C−C C ratios for the perovskite film at electron take-off angles of 0°, 30°, and 60°


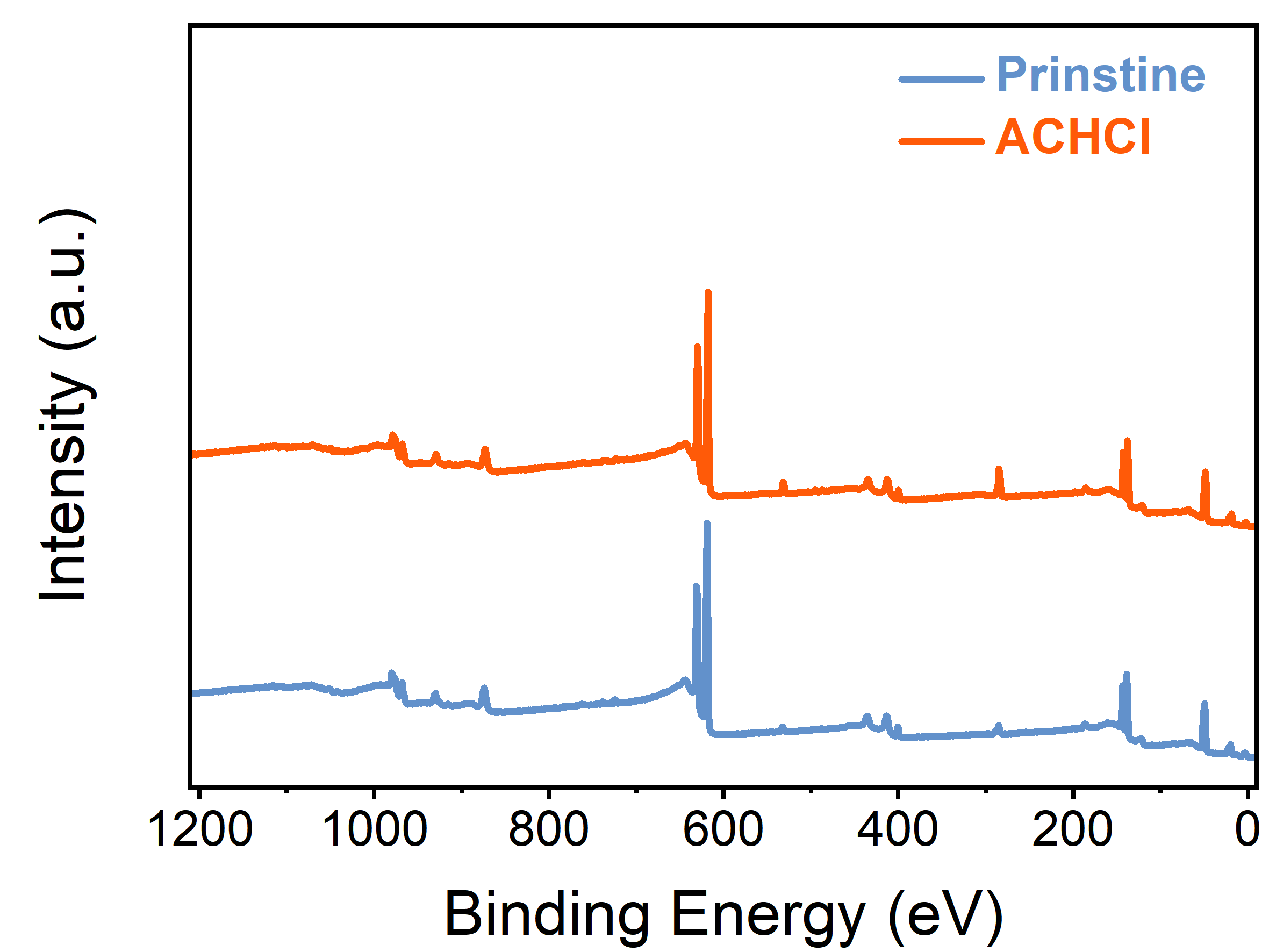


**Fig. S16** Full XPS spectra of pristine and ACHCl-treated perovskite films


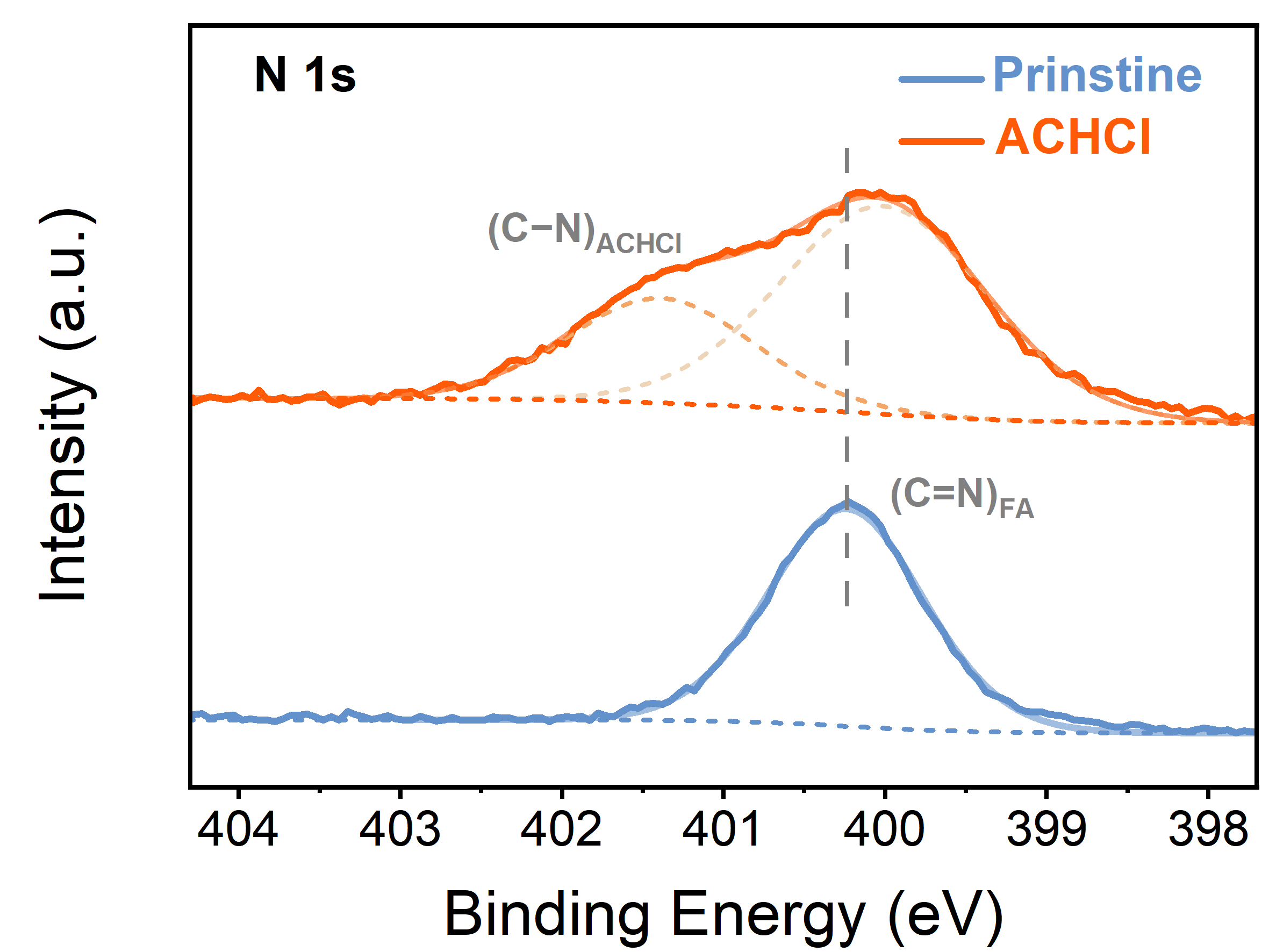


**Fig. S17** XPS spectra of N 1s for pristine and ACHCl-treated perovskite films


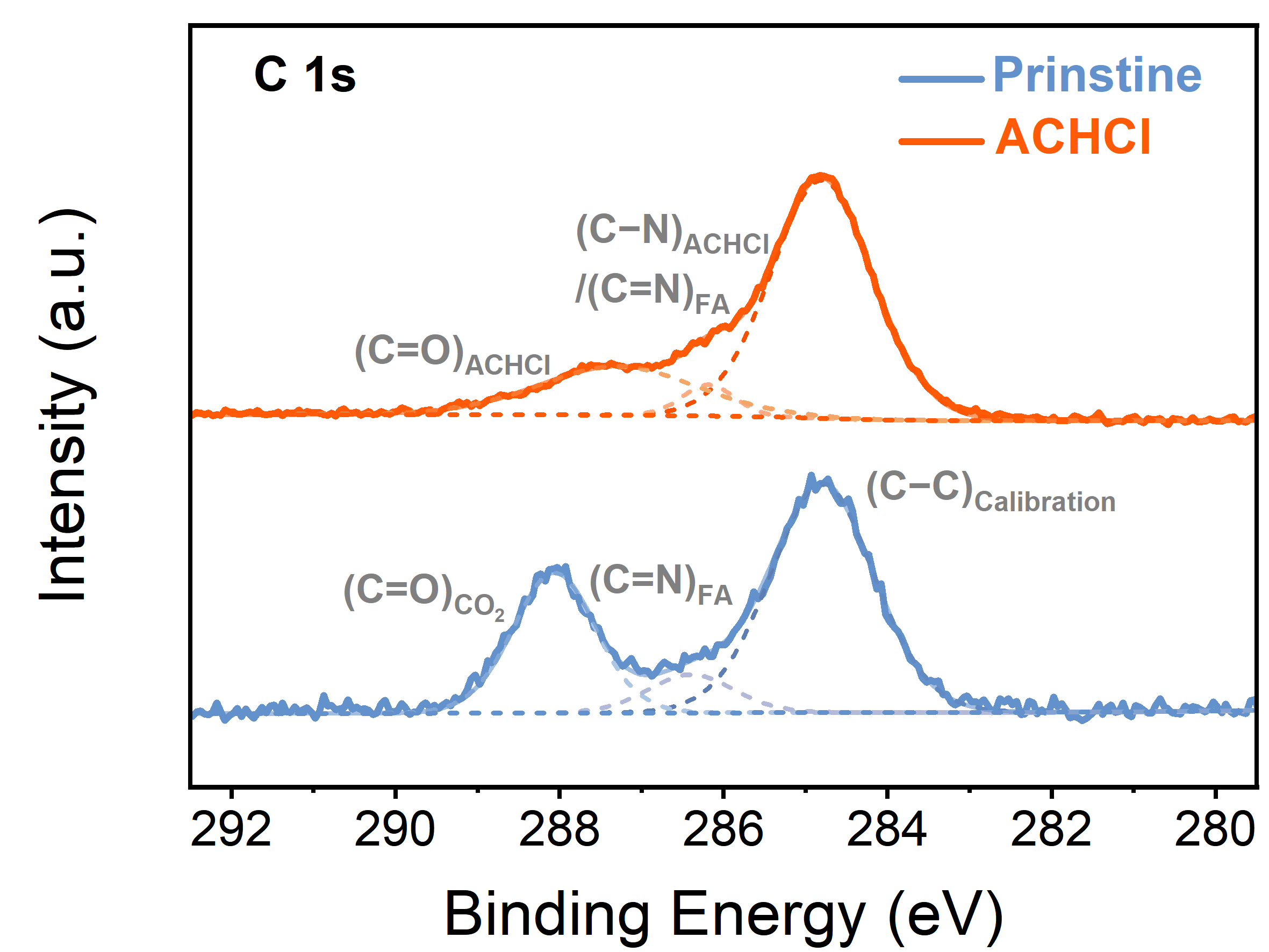


**Fig. S18** XPS spectra of C 1s for pristine and ACHCl-treated perovskite films


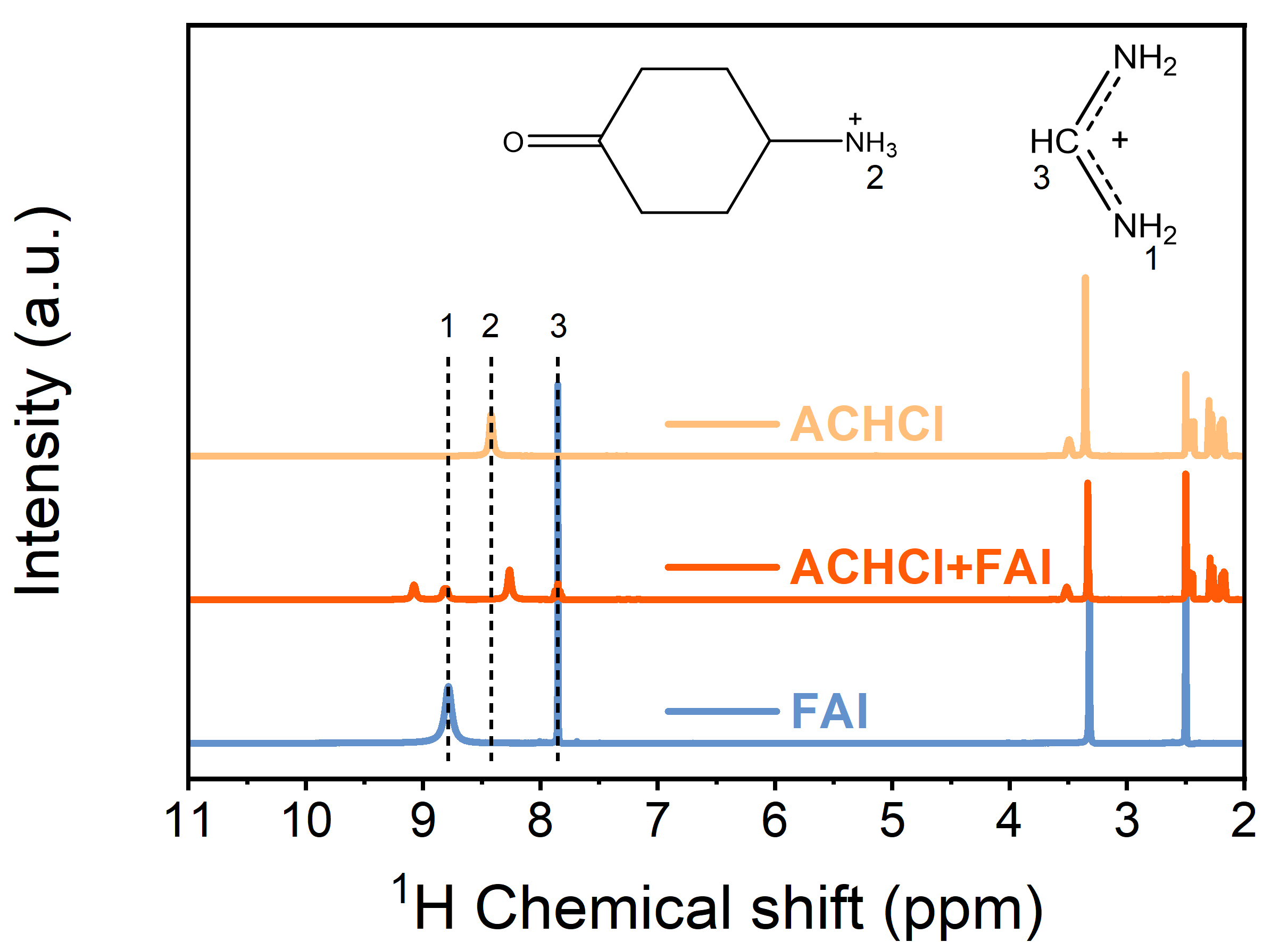


**Fig. S19** ^1^H NMR spectra of FAI, ACHCl + FAI, and ACHCl in DMSO-d_6_ solution


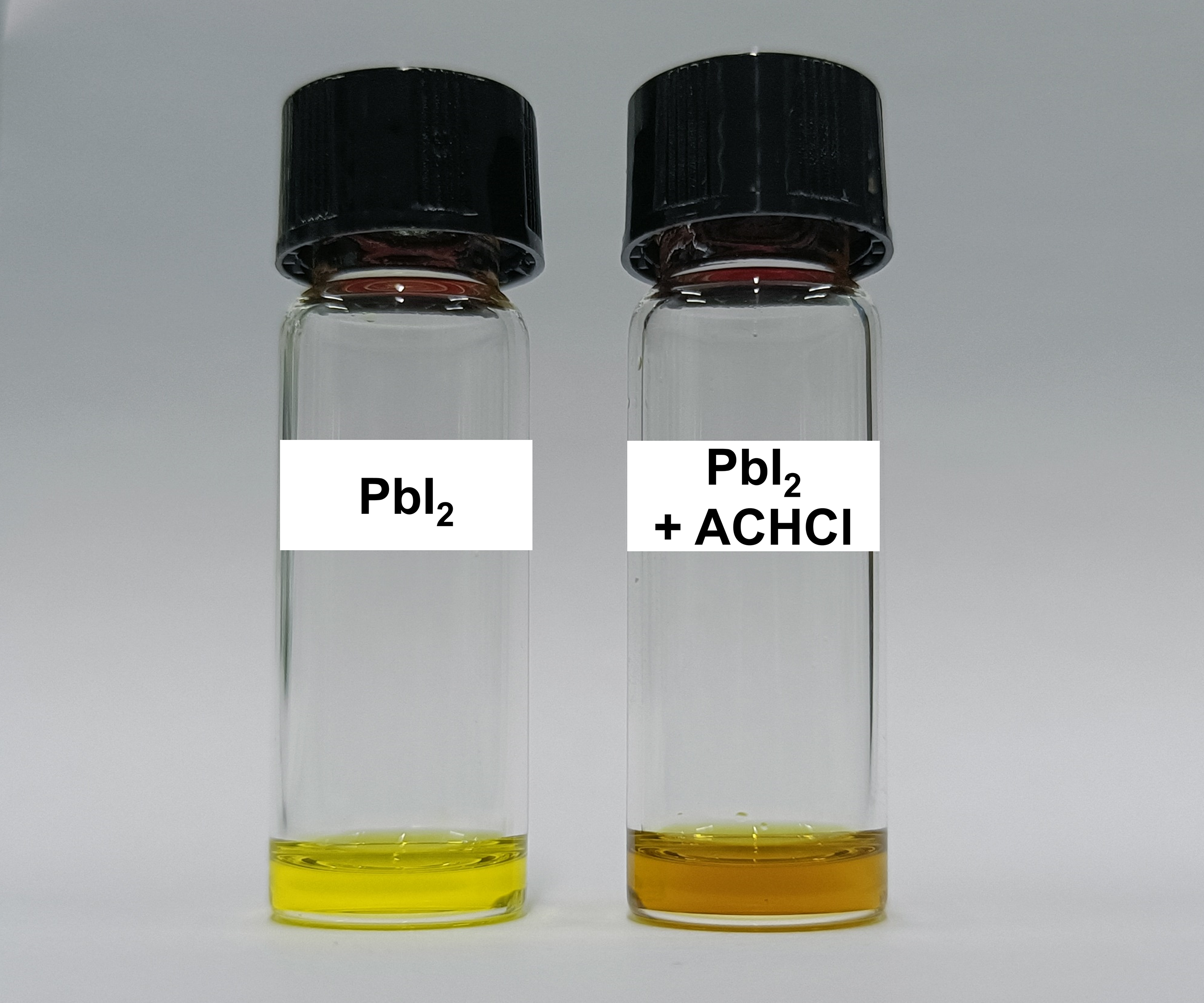


**Fig. S20** The digital photos of PbI_2_ and PbI_2_+ACHCl in mixed solution of DMF:DMSO (vol. ratio, 9:1)


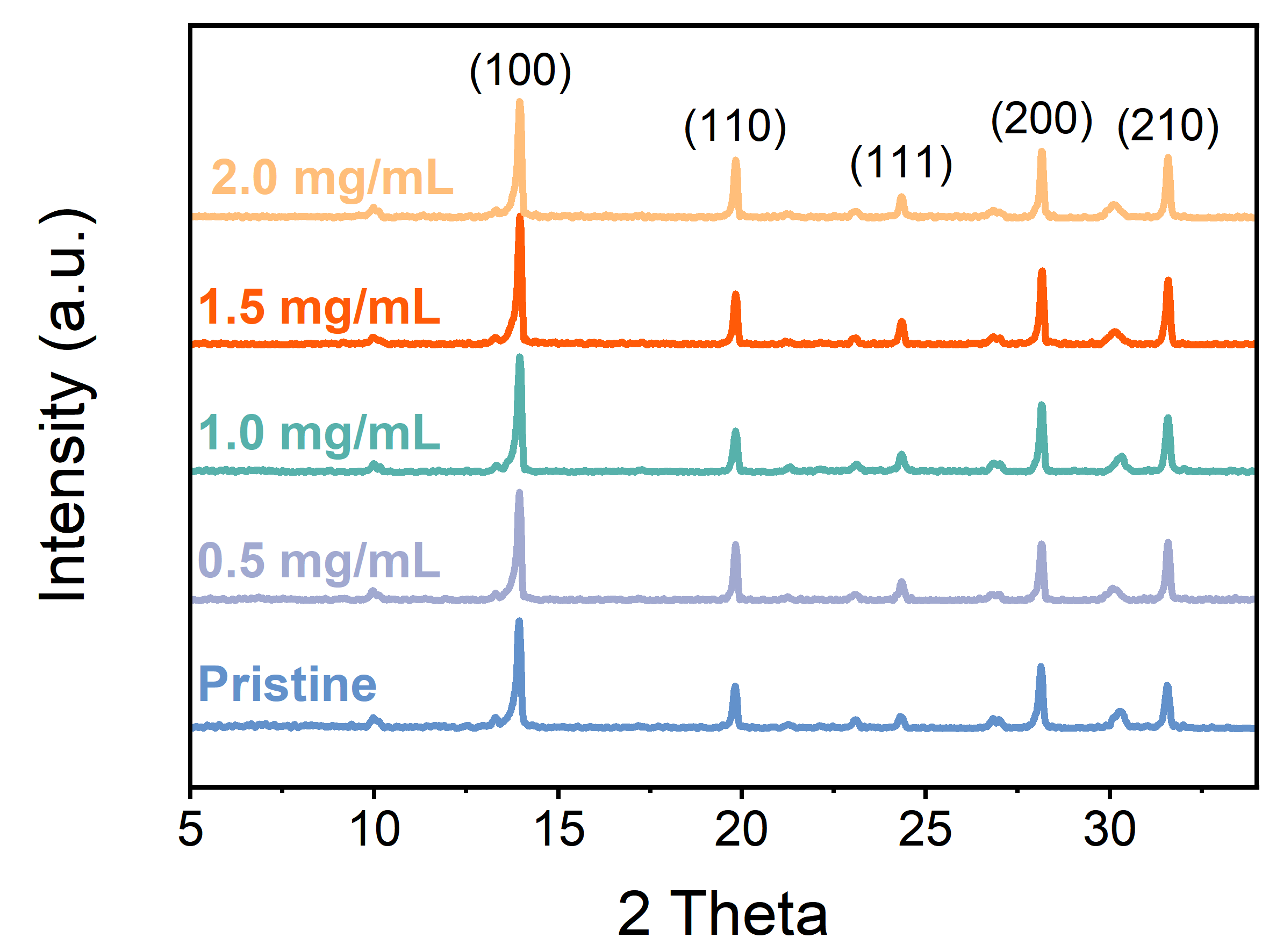


**Fig. S21** XRD patterns of perovskite films modified with different ACHCl concentrations


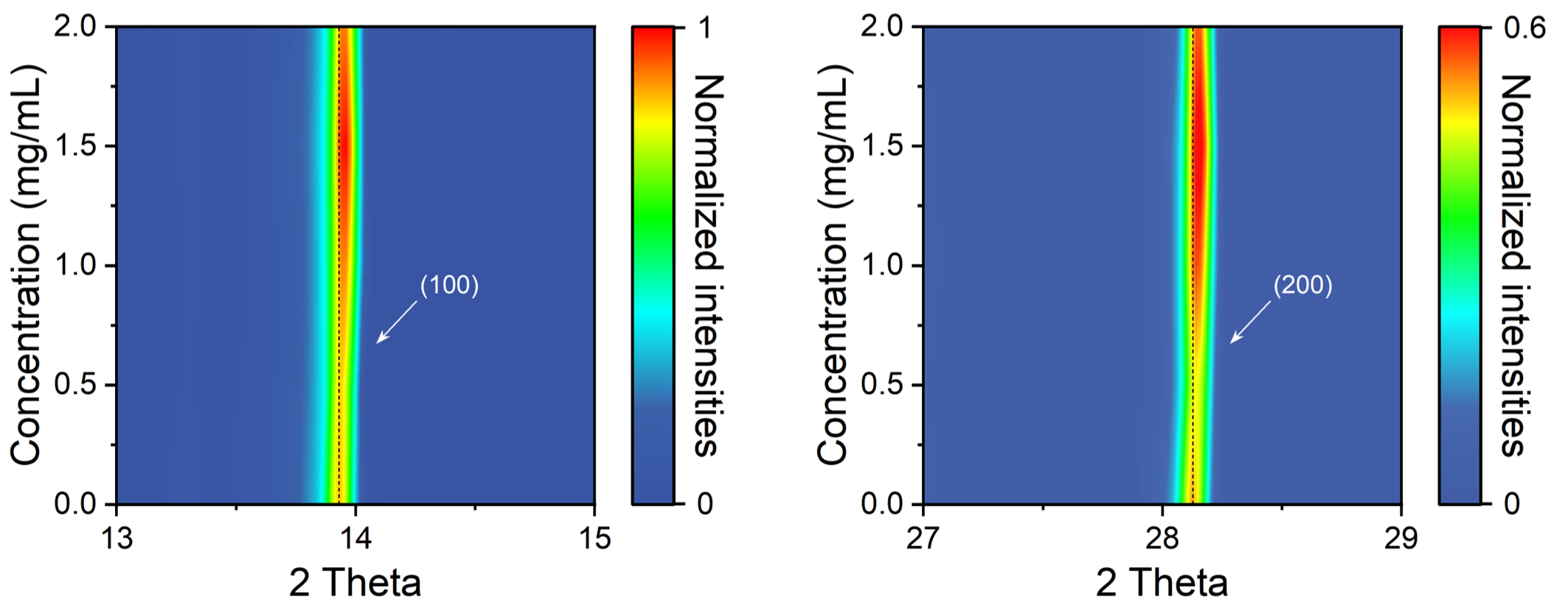


**Fig. S22** Pseudocolor map of (100) and (200) crystalline plane diffraction peaks for the perovskite films modified with different ACHCl concentrations


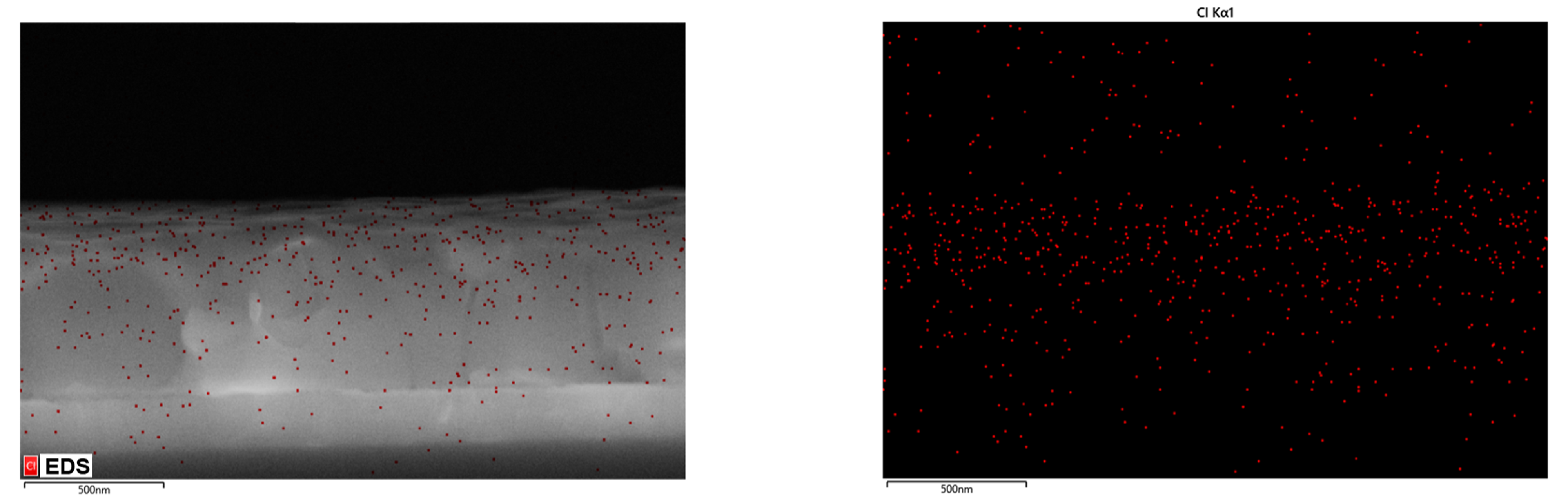


**Fig. S23** Cross-sectional SEM image of the ACHCl-treated perovskite film and the corresponding EDS elemental mapping of Cl


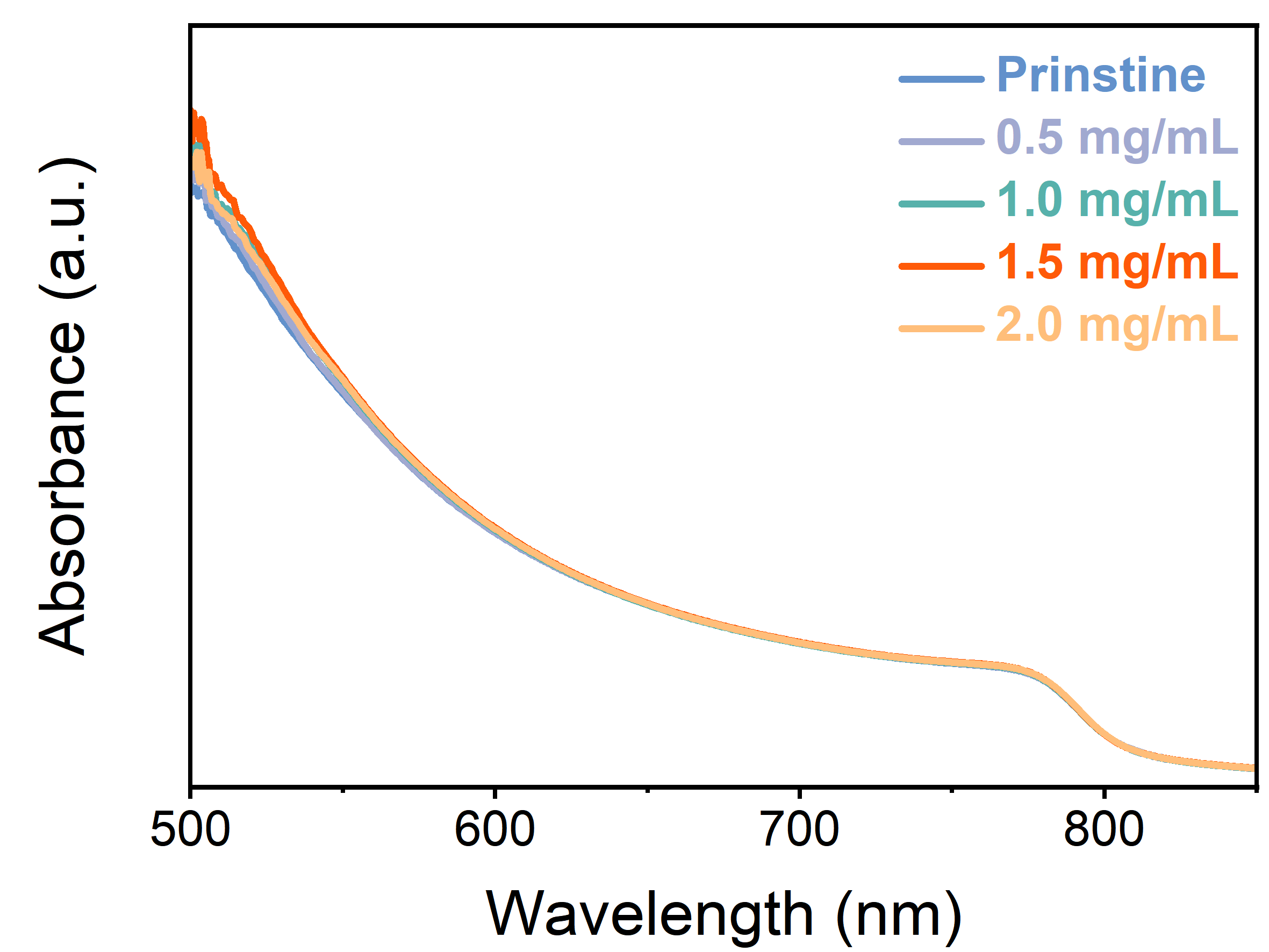


**Fig. S24** UV-Vis absorption spectra of perovskite films at different ACHCl concentrations


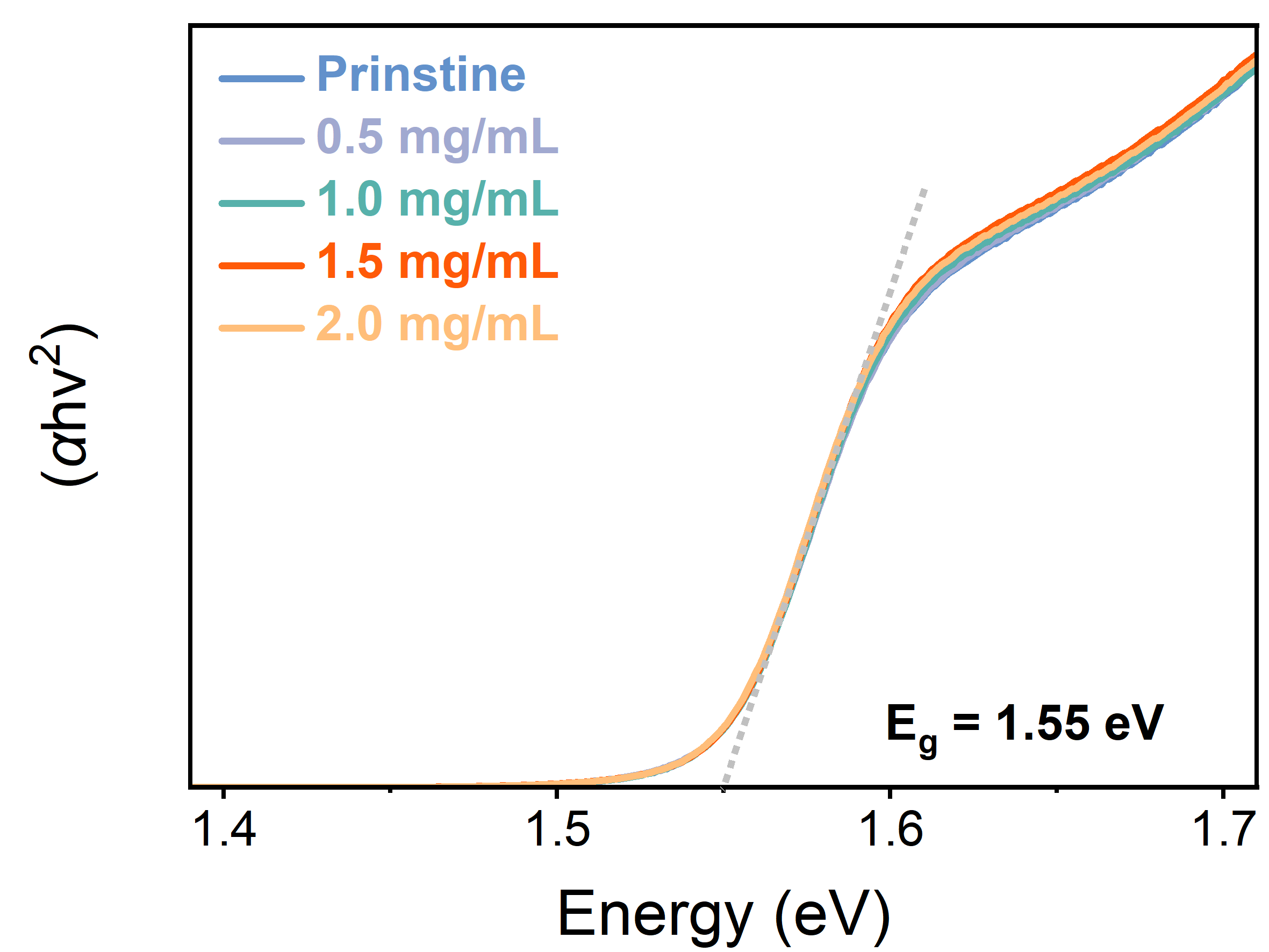


**Fig. S25** Tauc plots of perovskite films at different ACHCl concentrations


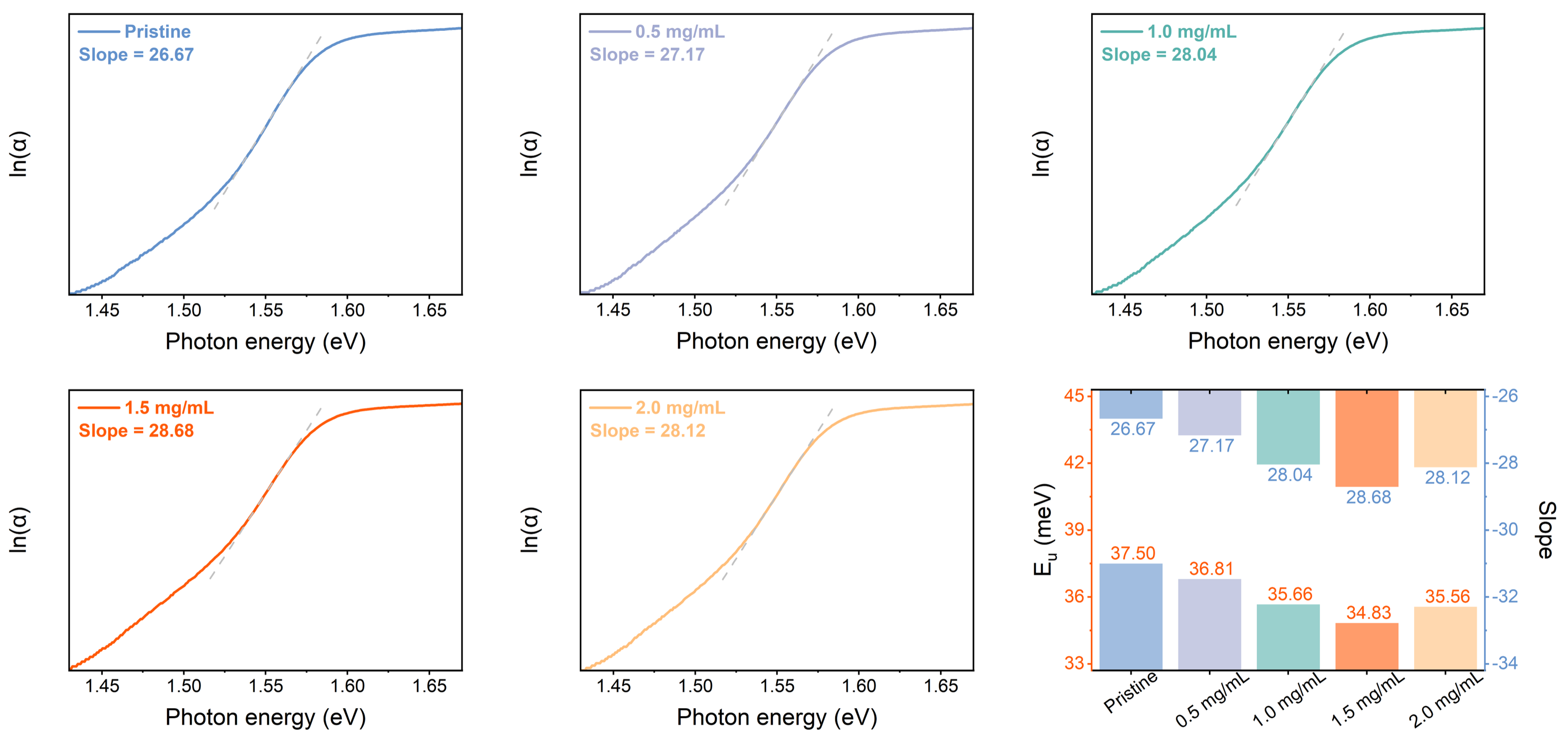


**Fig. S26** *E_U_* of perovskite films at different ACHCl concentrations


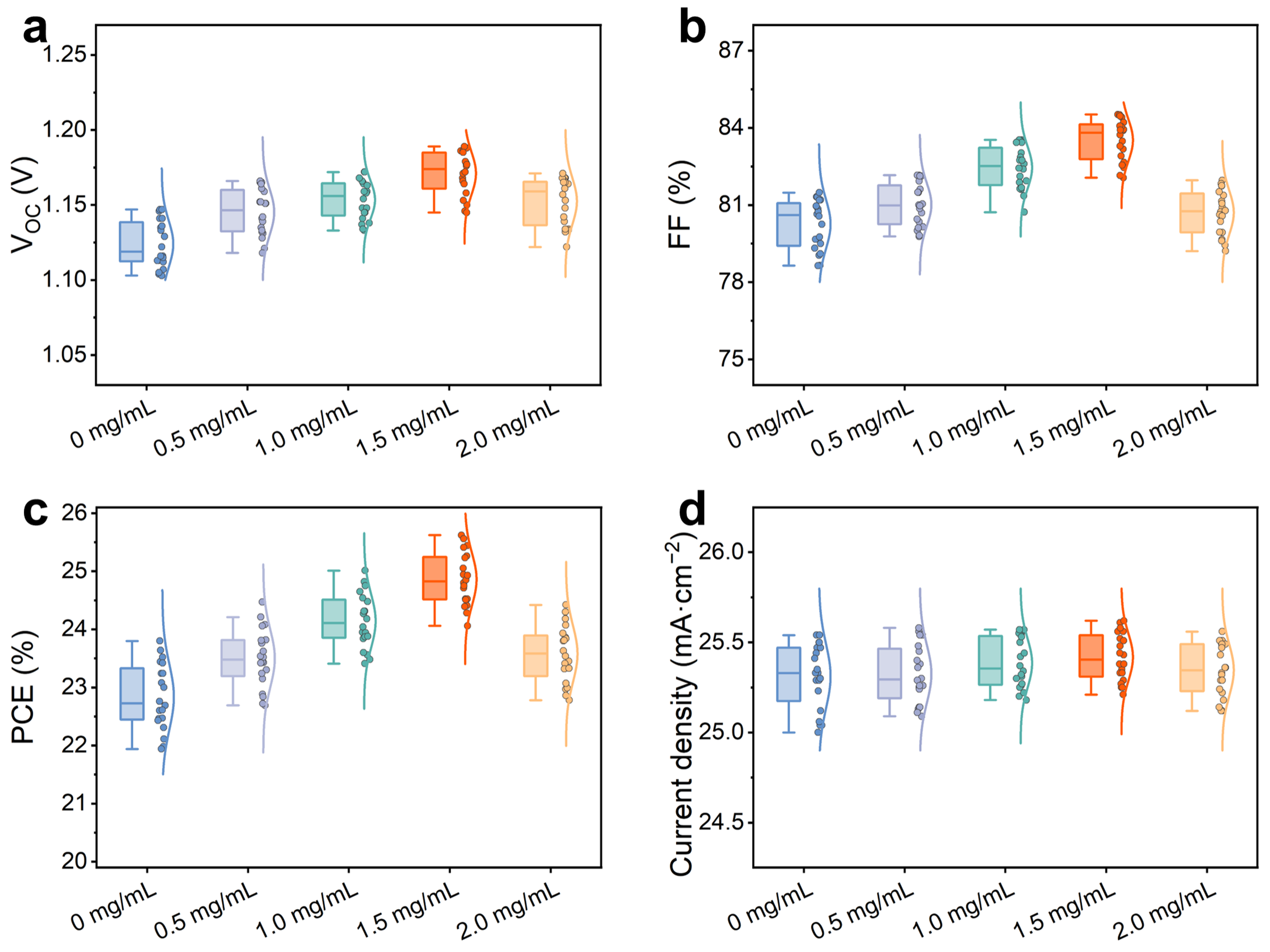


**Fig. S27** The statistics distribution of photovoltaic parameters obtained from *J-V* measurements for the devices treated with different ACHCl concentrations


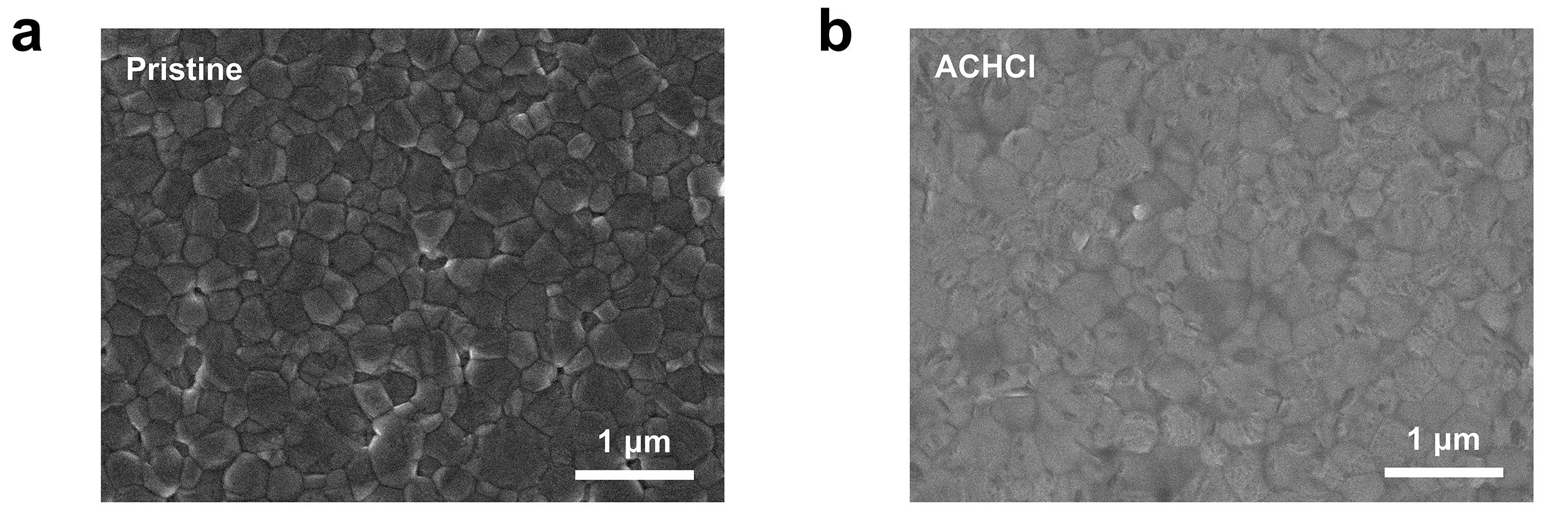


**Fig. S28** SEM images of pristine and ACHCl-treated perovskite films

**
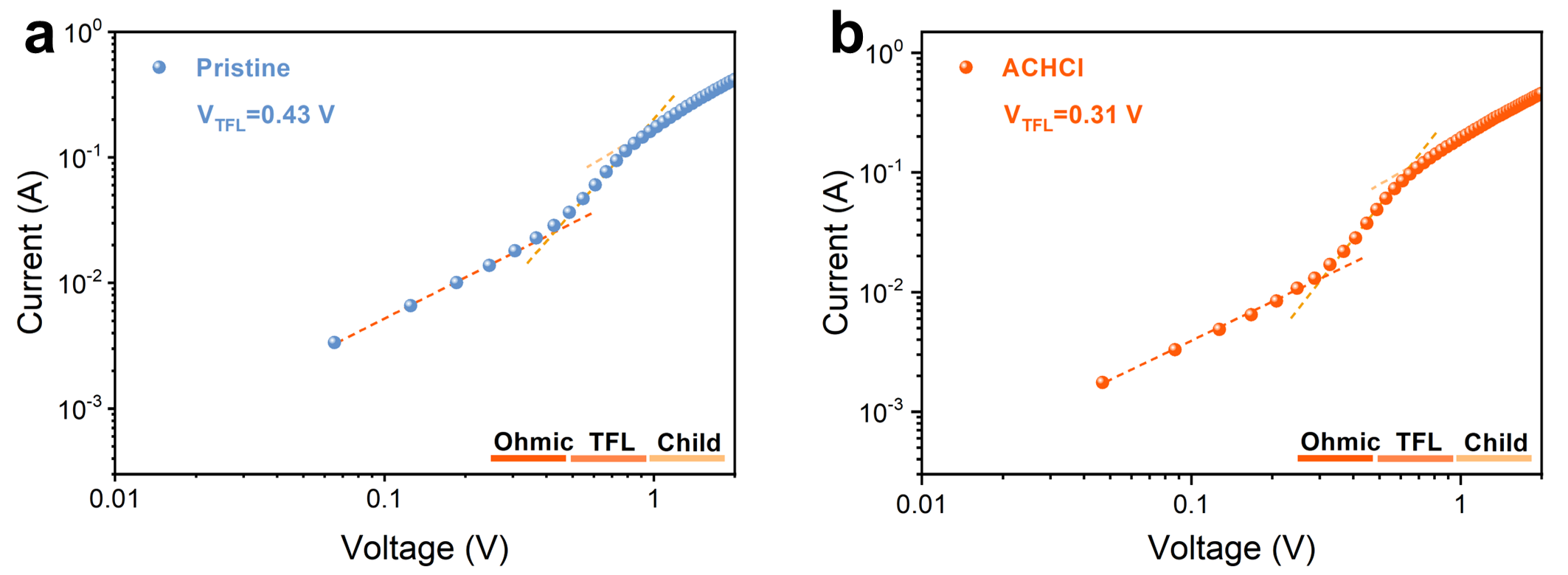
**

**Fig. S29** The SCLC curves of pristine and ACHCl-treated electron-only devices


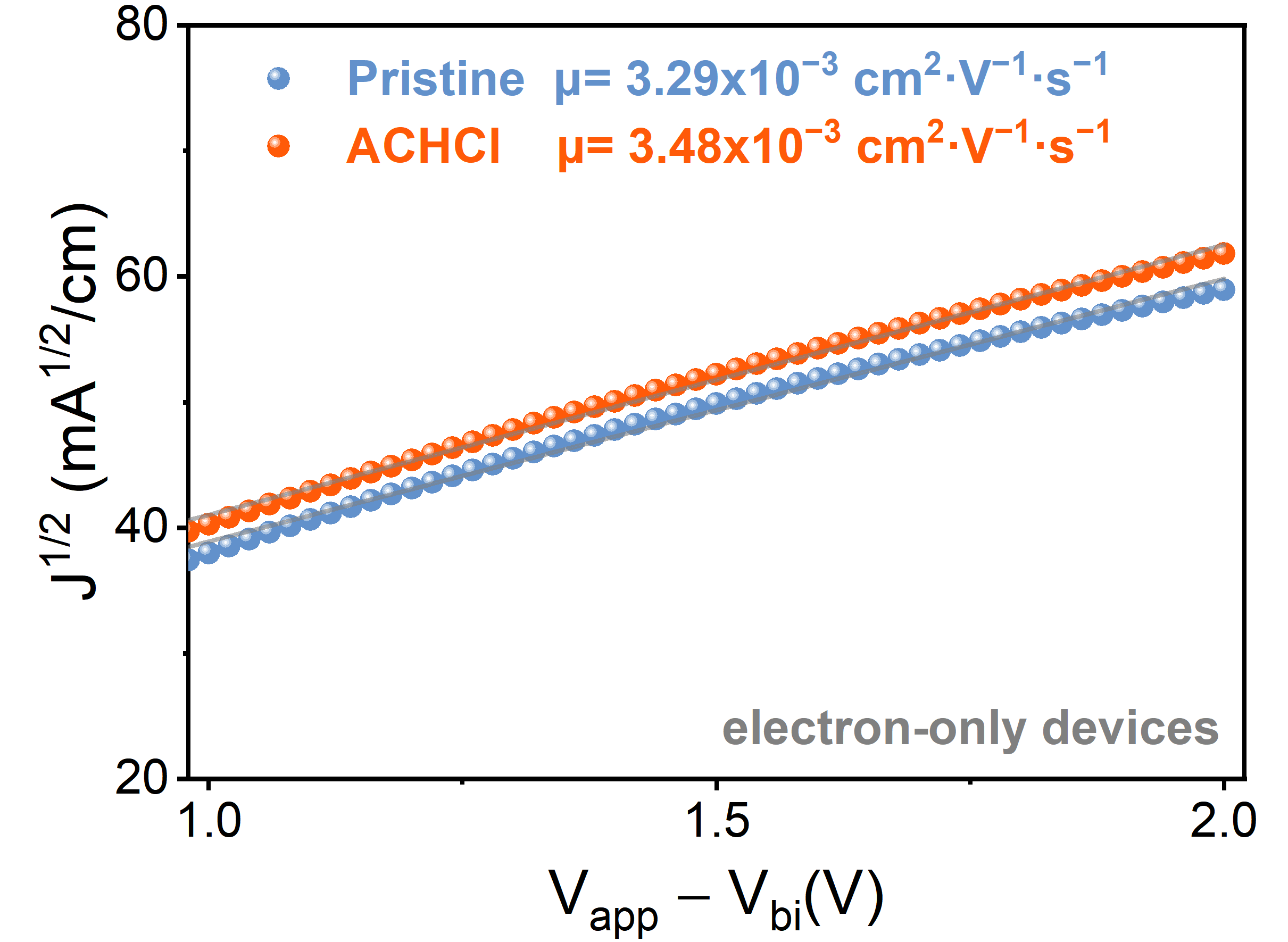


**Fig. S30** J^1/2^-V plots of pristine and ACHCl-treated the electron-only devices

**
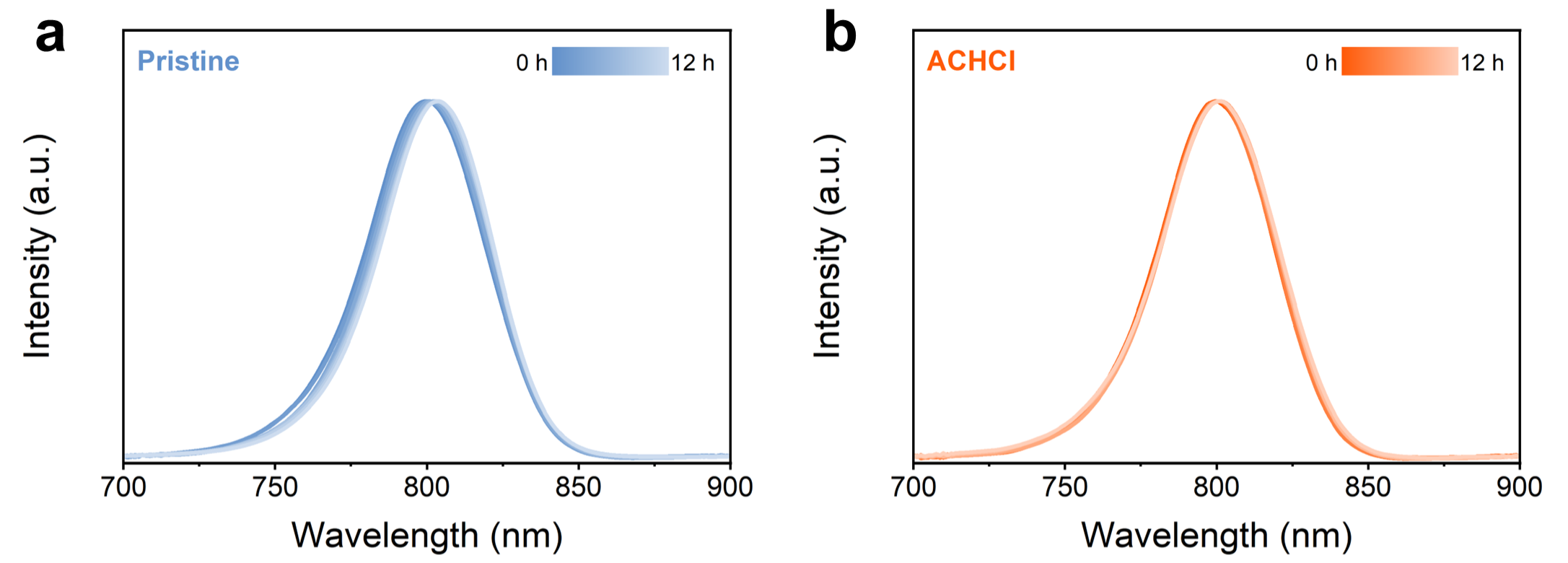
**

**Fig. S31** PL spectra of the pristine and ACHCl-treated perovskite films recorded at different illumination times under continuous one-sun irradiation

**
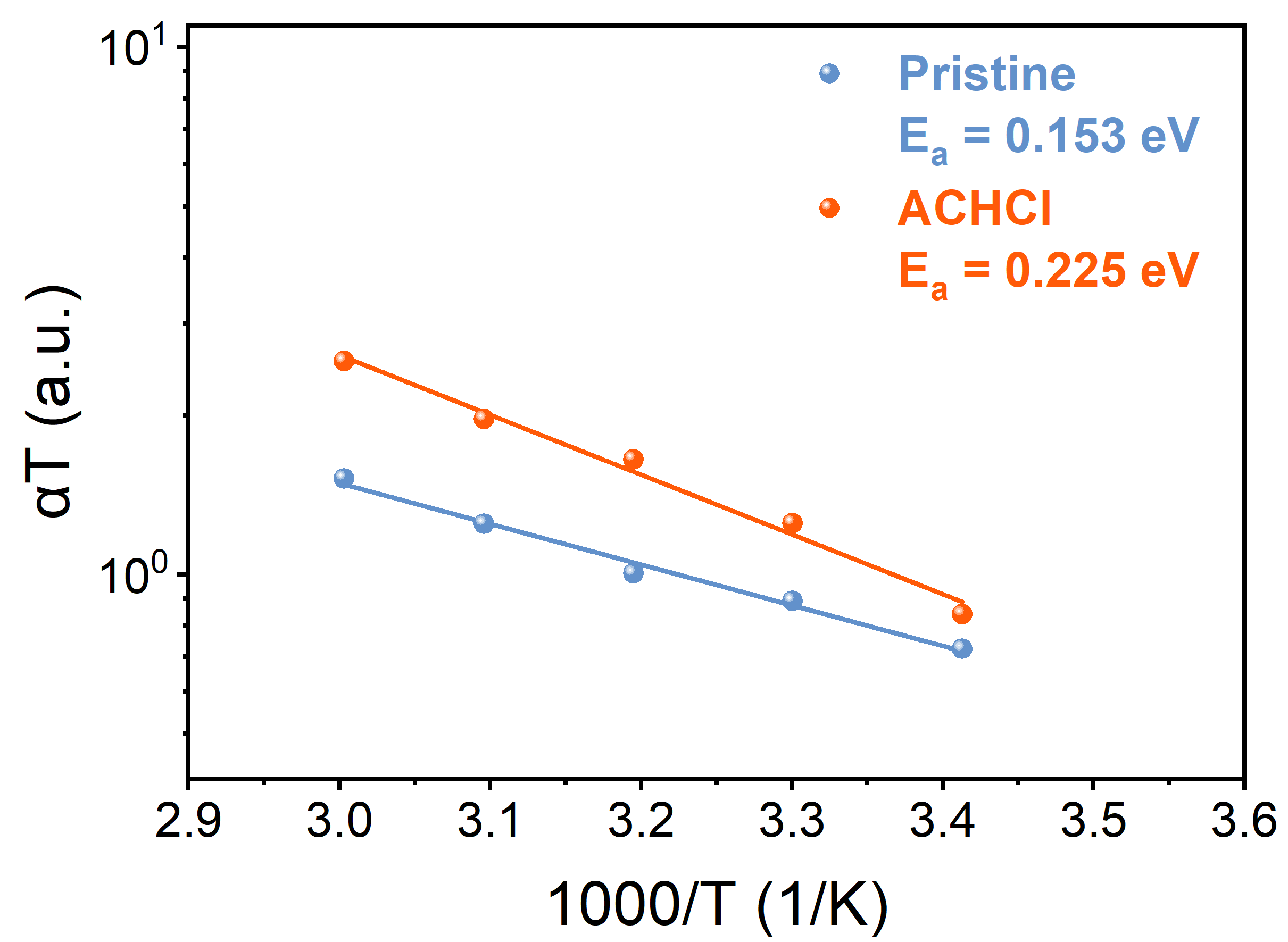
**

**Fig. S32** Temperature-dependent conductivity measurements of pristine and ACHCl-treated devices with an ITO/PVK/Ag structure


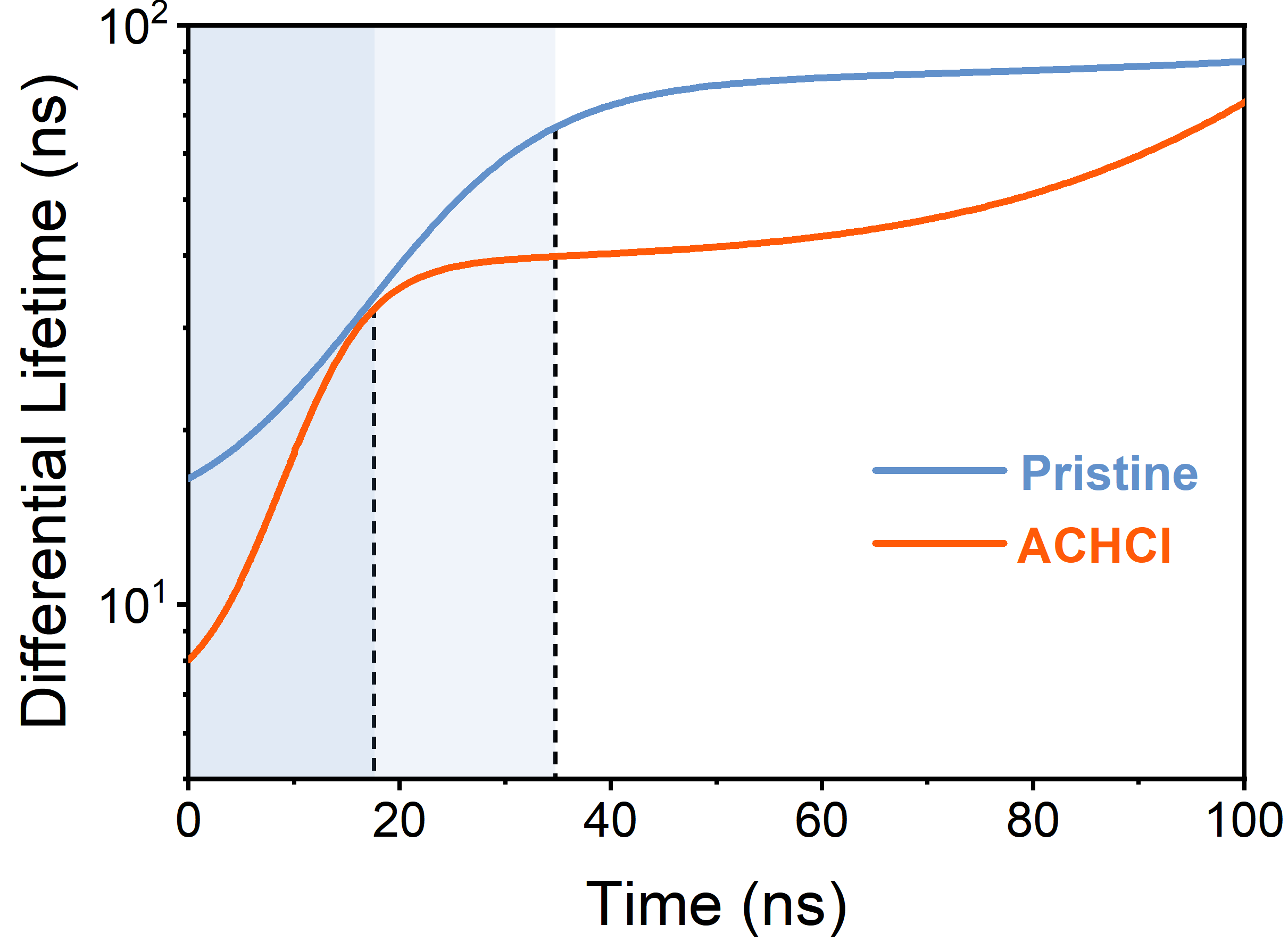


**Fig. S33** Differential lifetimes of pristine and ACHCl-treated perovskite films


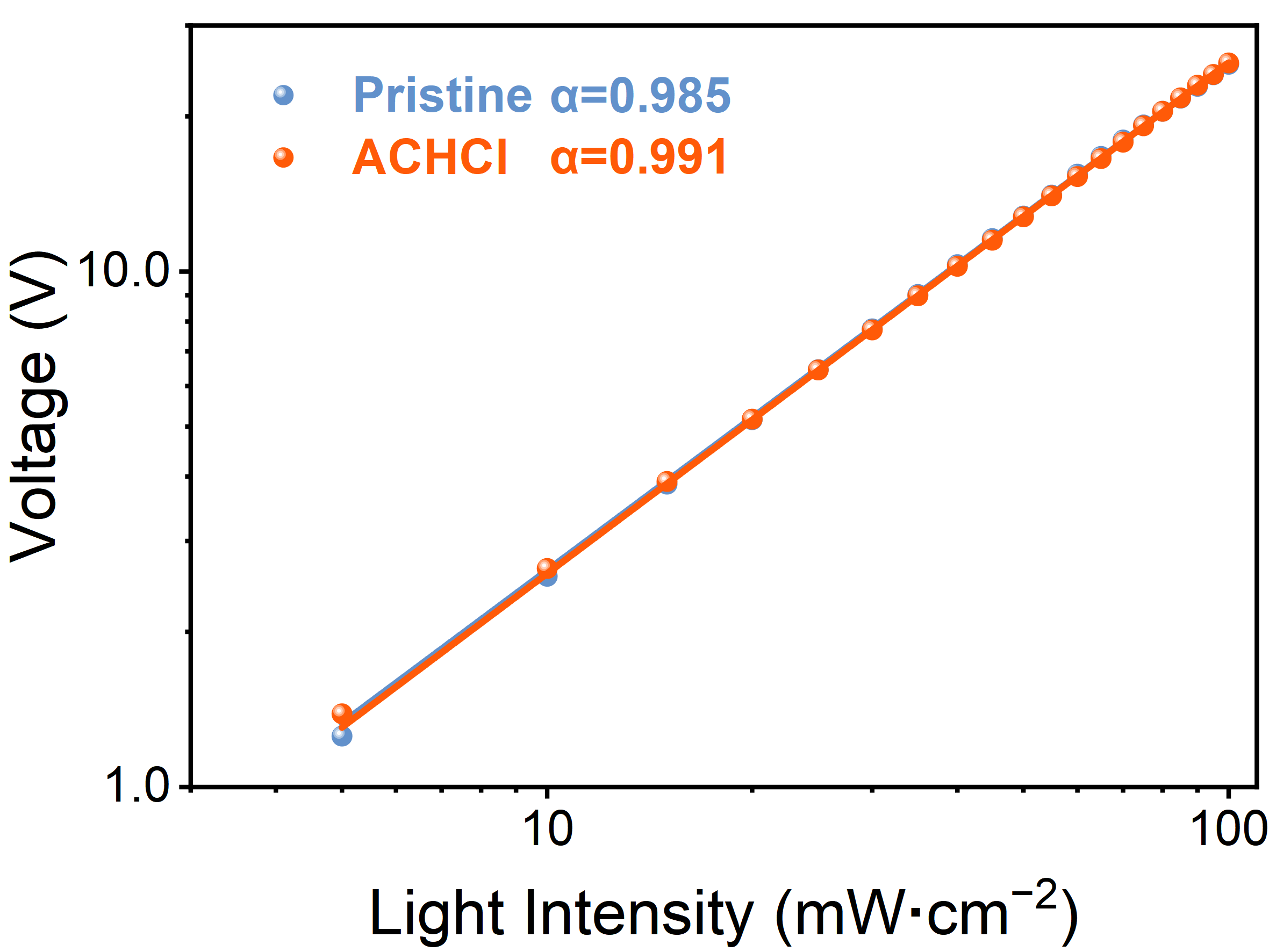


**Fig. S34** *J_SC_* versus light intensity curves for the pristine and ACHCl-treated devices


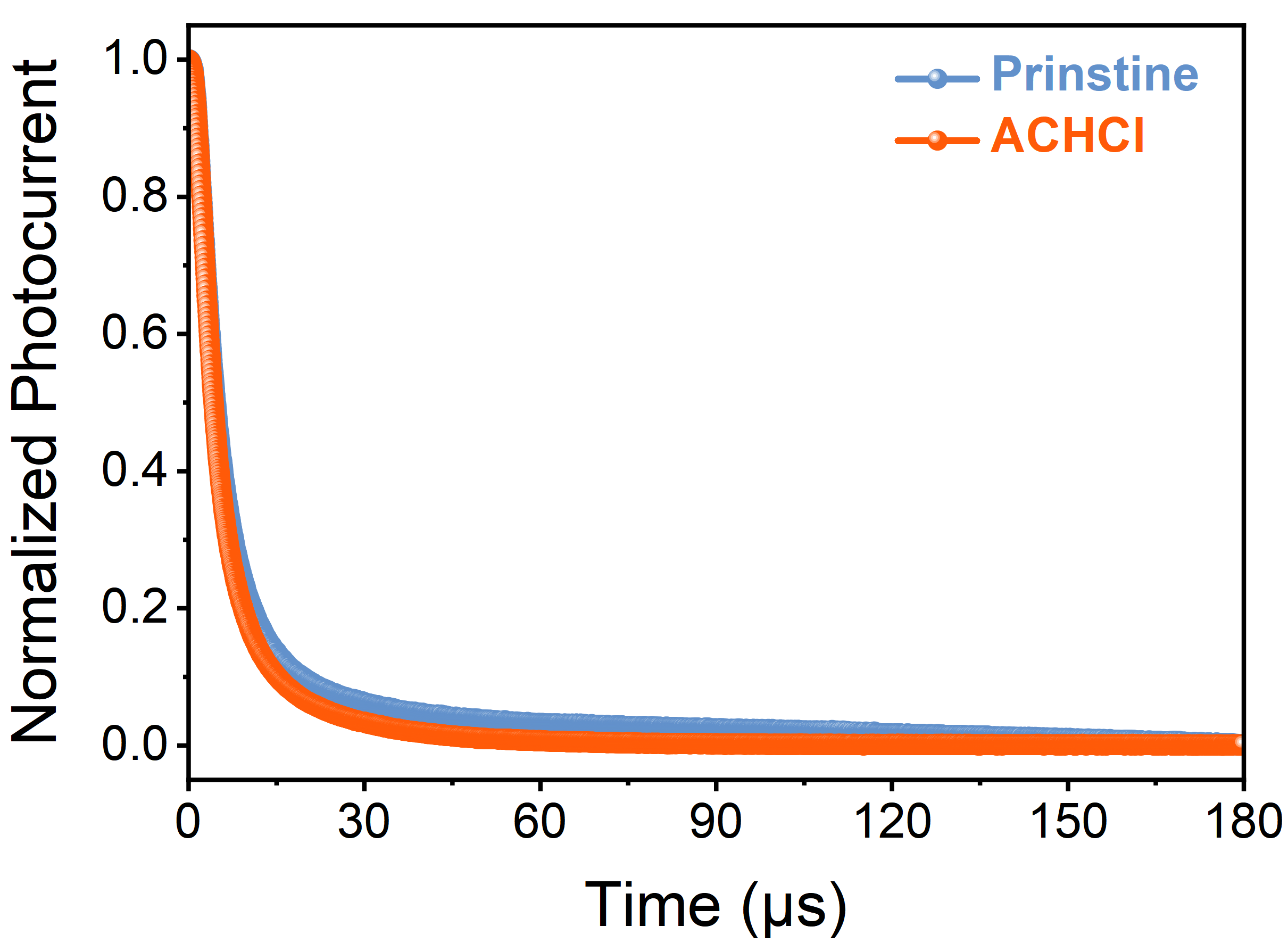


**Fig. S35** TPC curves of the pristine and ACHCl-treated devices

**
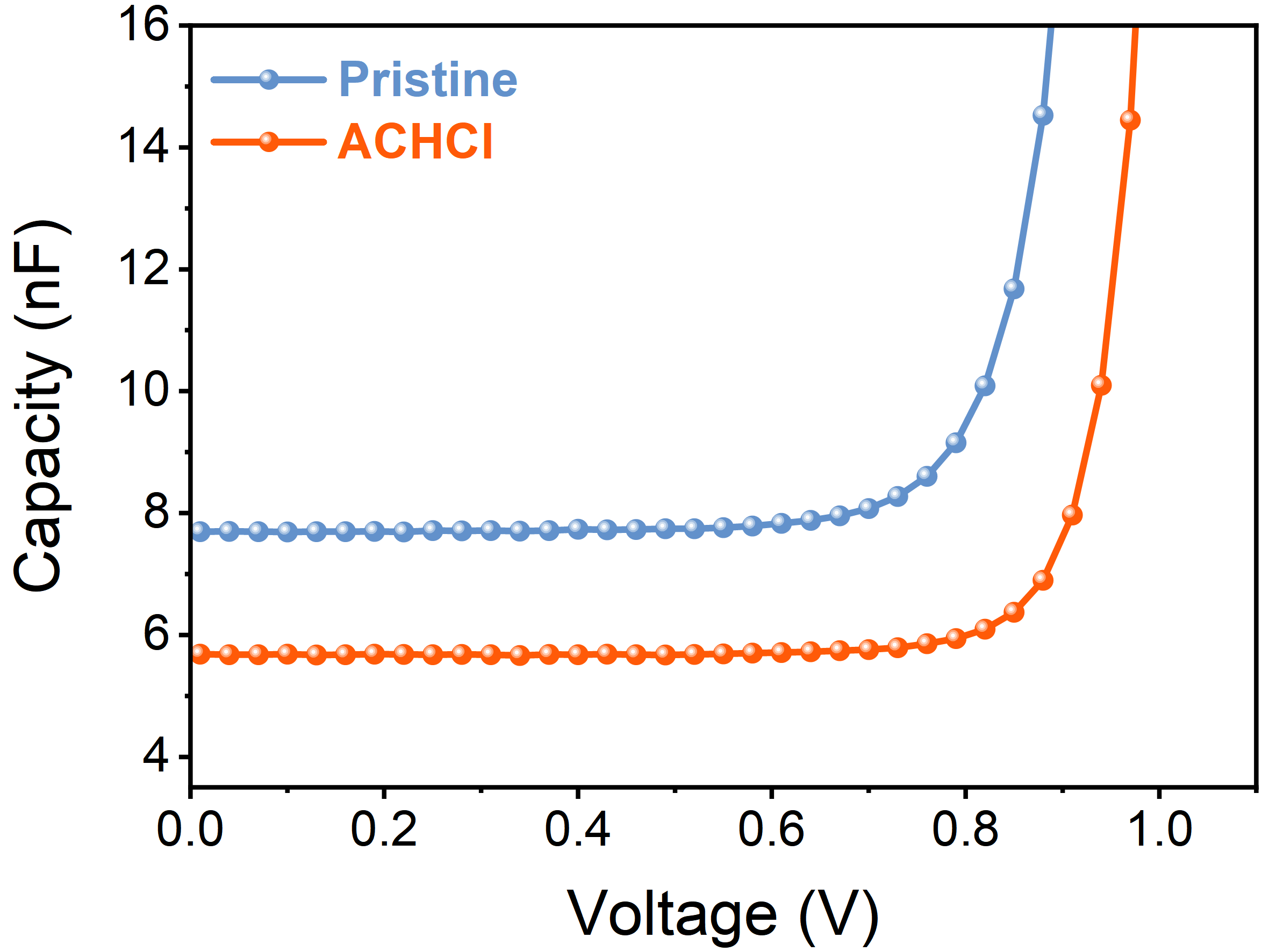
**

Fig. S36 C-V curves of the pristine and ACHCl-treated devices


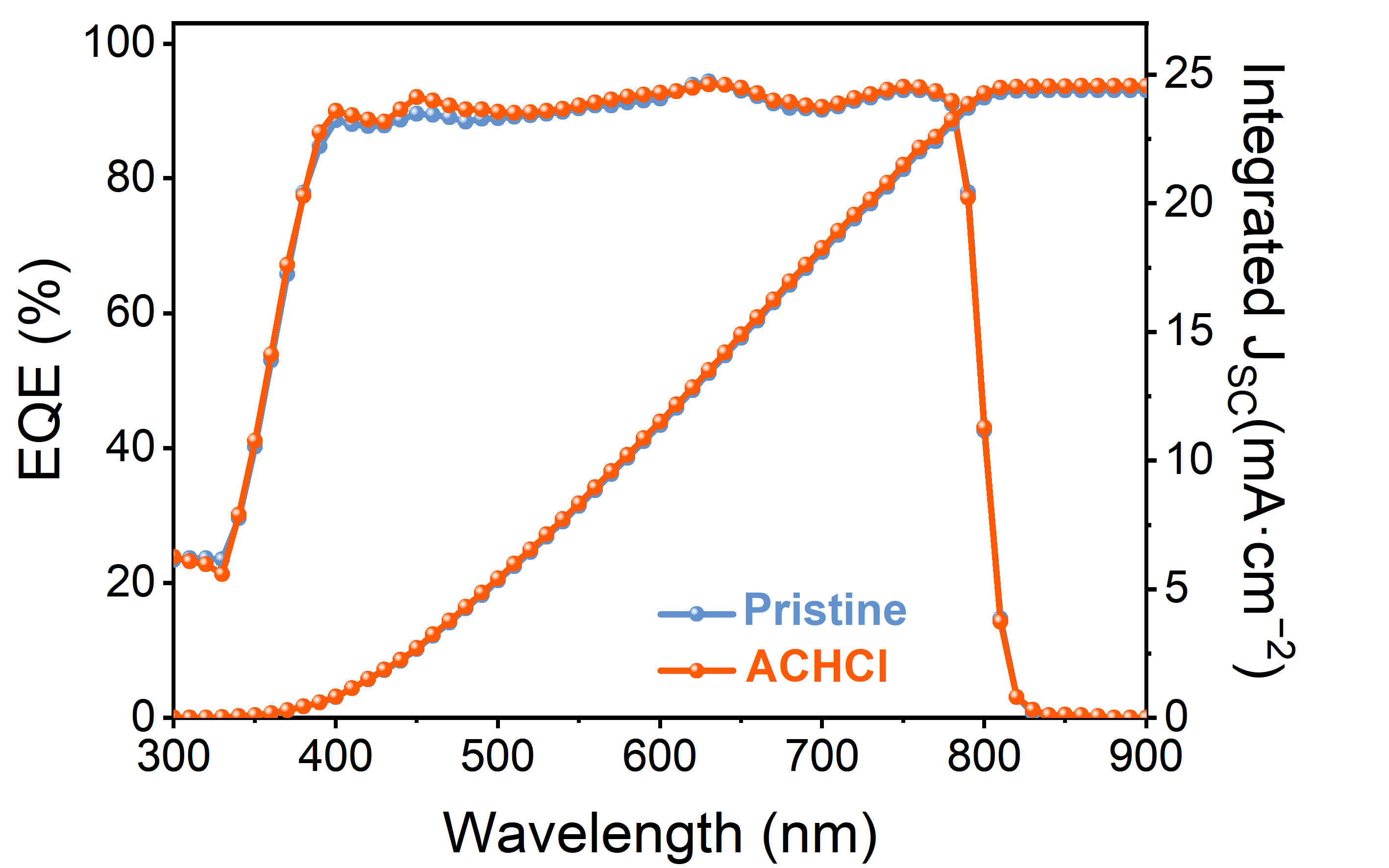


Fig. S37 IPCE spectra and integrated *J_SC_* curves of the pristine and ACHCl-treated devices


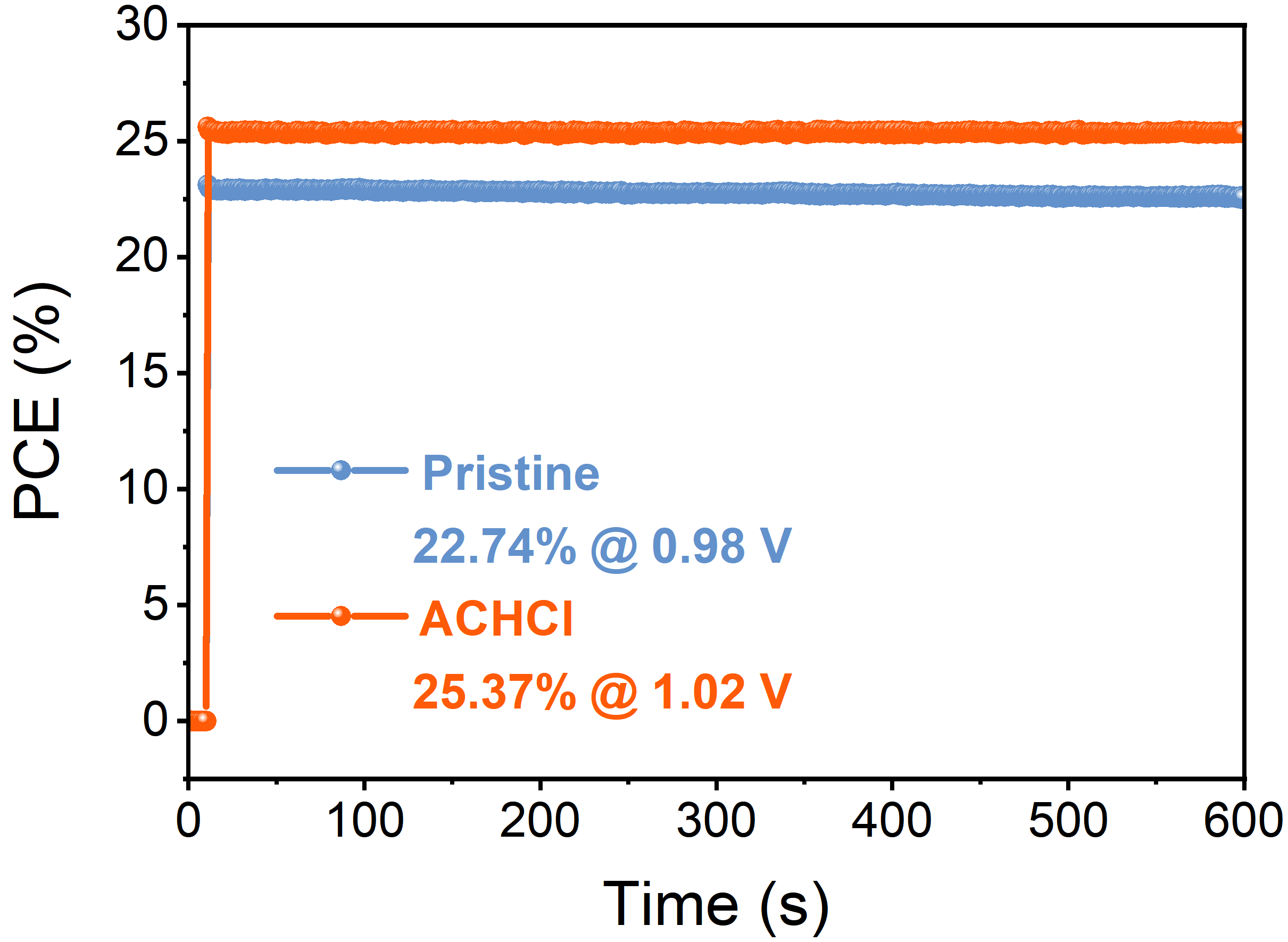


**Fig. S38** The steady-state output PCE of the pristine and ACHCl-treated devices


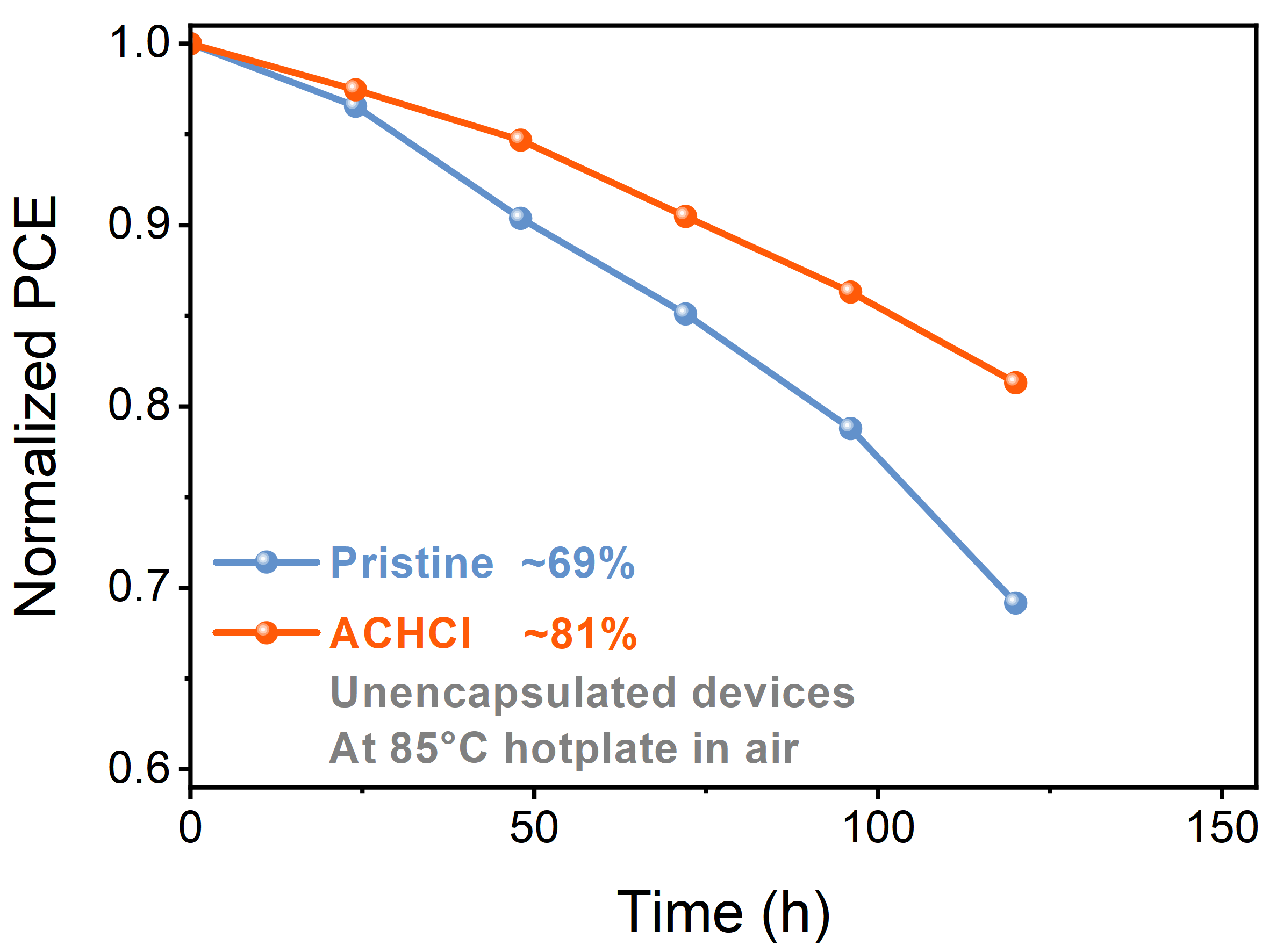


**Fig. S39** The thermal stability of the pristine and ACHCl-treated devices under heating at 85°C in air


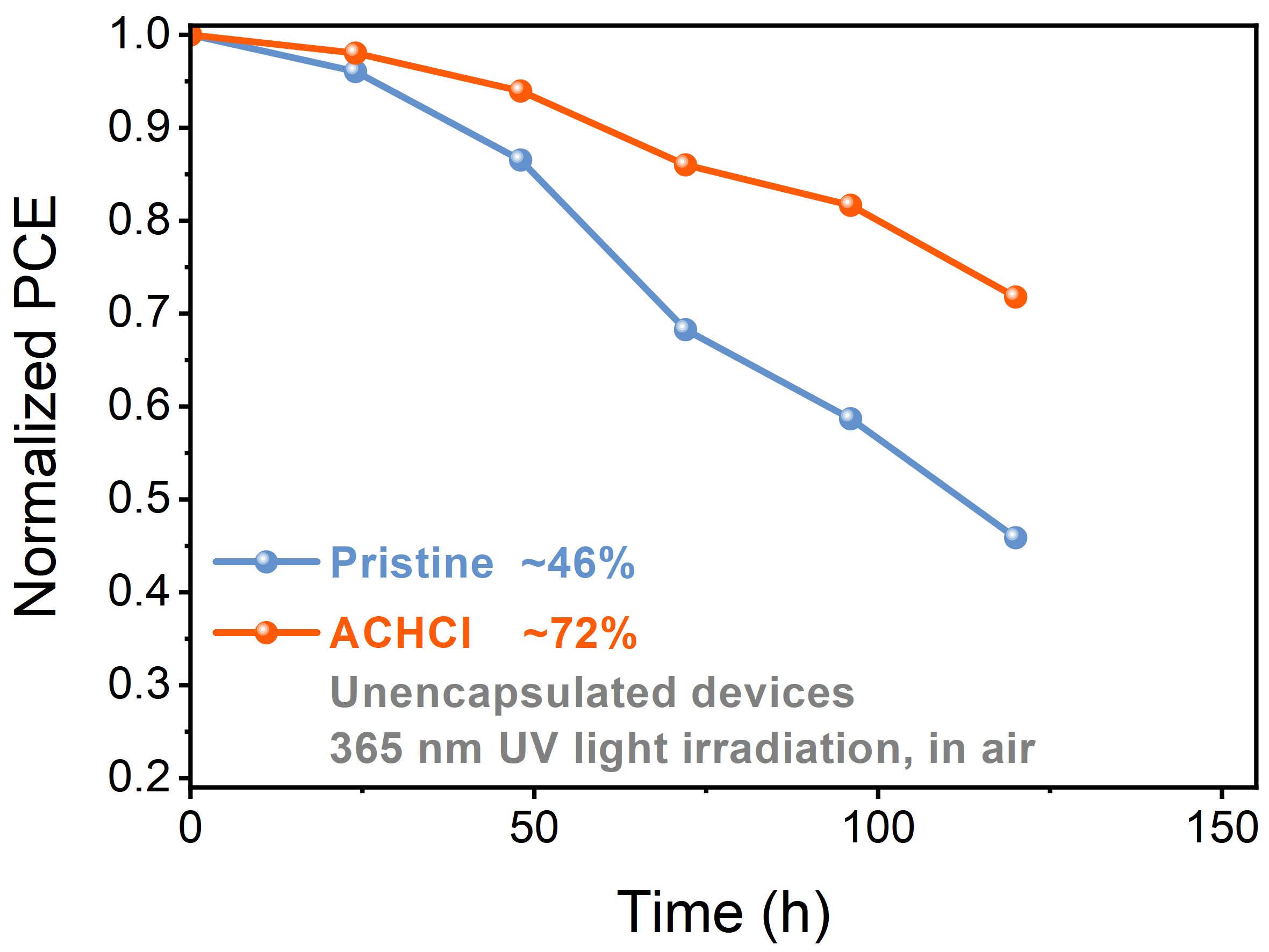


**Fig. S40** The UV-light stability of the pristine and ACHCl-treated devices under 365 nm UV irradiation (20 mW∙cm^−2^) from the glass side in air


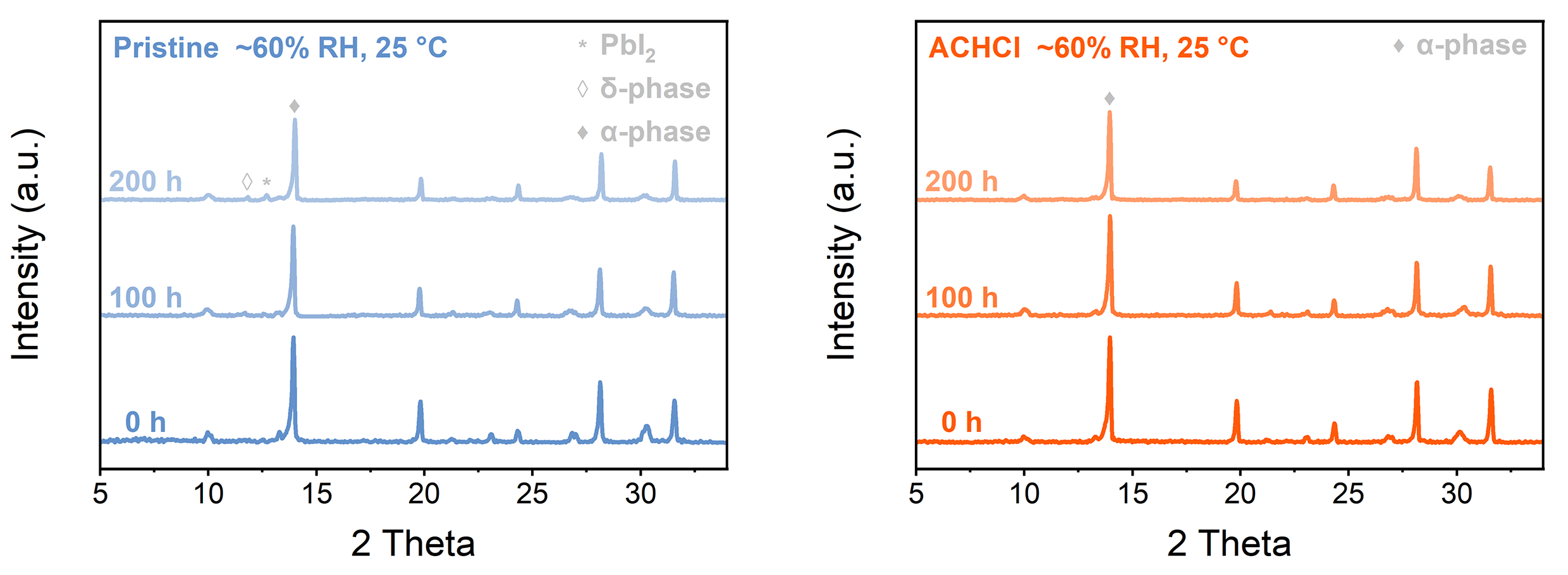


**Fig. S41** XRD pattern evolutions of the pristine and ACHCl-treated perovskite films (~60% RH)


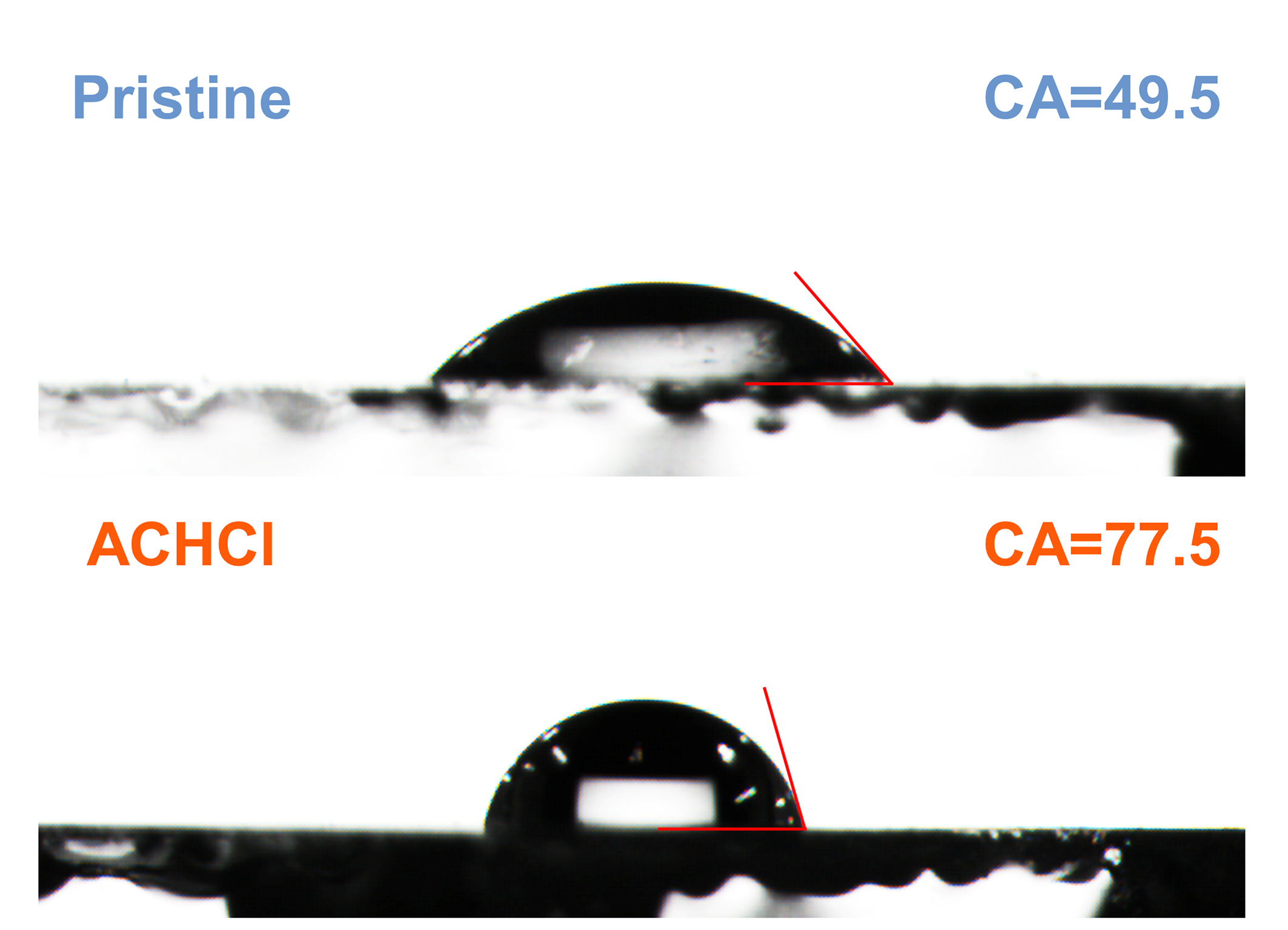


**Fig. S42** Water contact angle of the pristine and ACHCl-treated perovskite films

**S3 Supplementary Tables**

**Table S1** Photovoltaic data of devices treated with different molecules

| Device | *V_OC_* (V) | *J_SC_* (mA∙cm^−2^) | FF (%) | PCE (%) |
| --- | --- | --- | --- | --- |
| Pristine | 1.147 | 25.51 | 80.14 | 23.45 |
| CHD | 1.165 | 25.47 | 83.32 | 24.72 |
| ACHA | 1.178 | 25.50 | 81.16 | 24.38 |
| ACHCl | 1.190 | 25.53 | 84.74 | 25.75 |

**Table S2** Statistical data on the photovoltaic data of devices treated with different molecules

| Device | *V_OC_* (V) | *J_SC_* (mA∙cm^−2^) | FF (%) | PCE (%) |
| --- | --- | --- | --- | --- |
| Pristine | 1.126±0.017 | 25.39±0.17 | 78.91±0.86 | 22.55±0.51 |
| CHD | 1.144±0.016 | 25.41±0.18 | 82.38±0.81 | 23.92±0.51 |
| ACHA | 1.159±0.014 | 25.42±0.16 | 80.12±0.84 | 23.59±0.49 |
| ACHCl | 1.178±0.014 | 25.42±0.13 | 83.75±0.78 | 25.06±0.38 |

**Table S3** Statistical data on the photovoltaic data of devices at different concentrations

| Device | *V_OC_* (V) | *J_SC_* (mA∙cm^−2^) | FF (%) | PCE (%) |
| --- | --- | --- | --- | --- |
| Pristine | 1.124±0.016 | 25.31±0.18 | 80.26±0.96 | 22.84±0.57 |
| 0.5 mg∙mL^-1^ | 1.145±0.015 | 25.33±0.16 | 81.02±0.83 | 23.50±0.50 |
| 1.0 mg∙mL^-1^ | 1.153±0.013 | 25.38±0.14 | 82.46±0.83 | 24.15±0.47 |
| 1.5 mg∙mL^-1^ | 1.171±0.014 | 25.42±0.13 | 83.52±0.81 | 24.86±0.45 |
| 2.0 mg∙mL^-1^ | 1.152±0.016 | 25.35±0.14 | 80.71±0.86 | 23.58±0.49 |

**Table S4** Energy level parameters of pristine and ACHCl-treated perovskite films

| Sample | E_F_ (eV) | E_VBM_ (eV) | E_CBM_ (eV) | E_g_ (eV) |
| --- | --- | --- | --- | --- |
| Pristine | −4.58 | −5.63 | −4.08 | 1.55 |
| ACHCl | −4.40 | −5.67 | −4.12 | 1.55 |

**Table S5** Fitting parameters for the TRPL spectra of samples with ITO/PVK structure

| Sample | τ_avg_ (ns) | τ_1_ (ns) | τ_2_ (ns) | A_1_ (%) | A_2_ (%) |
| --- | --- | --- | --- | --- | --- |
| Pristine | 93.49 | 10.23 | 102.61 | 52.36 | 47.64 |
| ACHCl | 156.45 | 13.40 | 167.14 | 48.25 | 51.75 |

**Table S6** Electrical impedance for the dark-state impedance spectra of different devices

| Device | R_s_ (Ω) | R_rec_ (Ω) |
| --- | --- | --- |
| Pristine | 8.01 | 2281 |
| ACHCl | 7.62 | 3501 |

**Table S7** Statistical data on the photovoltaic data of different devices

| Device | *V_OC_* (V) | *J_SC_* (mA∙cm^−2^) | FF (%) | PCE (%) |
| --- | --- | --- | --- | --- |
| Pristine | 1.137±0.012 | 25.33±0.16 | 80.12±0.91 | 23.07±0.42 |
| ACHCl | 1.174±0.012 | 25.48±0.14 | 83.66±0.81 | 25.01±0.37 |

**Table S8** Photovoltaic data of pristine and ACHCl-modified champion devices

| Device | Scan direction | *V_OC_* (V) | *J_SC_* (mA∙cm^−2^) | FF (%) | PCE (%) | Hysteresis index |
| --- | --- | --- | --- | --- | --- | --- |
| Pristine | Reverse scan | 1.153 | 25.53 | 81.47 | 23.98 | 0.039 |
|  | Forward scan | 1.150 | 25.48 | 78.65 | 23.03 |  |
| ACHCl | Reverse scan | 1.200 | 25.62 | 84.97 | 26.12 | 0.007 |
|  | Forward scan | 1.200 | 25.58 | 84.49 | 25.92 |  |

**Table S9** The FF loss of the pristine and ACHCl-modified devices

| Device | FF_SQ_ (%) | FF_max_ (%) | FF_measured_ (%) |
| --- | --- | --- | --- |
| Pristine | 90.20 | 84.51 | 81.47 |
| ACHCl | 90.20 | 86.25 | 84.97 |

**Supplementary References**

1. J. Wang, S. Fu, L. Huang, Y. Lu, X. Liu et al., Heterojunction engineering and ideal factor optimization toward efficient MINP perovskite solar cells. Adv. Energy Mater. **11**(48), 2102724 (2021). <https://doi.org/10.1002/aenm.202102724>
2. C.-H. Chen, F. Hu, Z.-H. Su, Y.-J. Yu, K.-L. Wang et al., Spring-like ammonium salt assisting stress release for low-temperature deposited FAPbI3 films toward flexible photovoltaic application. Adv. Funct. Mater. **33**(15), 2213661 (2023). <https://doi.org/10.1002/adfm.202213661>
3. J. Chen, X. Zhao, S.-G. Kim, N.-G. Park, Multifunctional chemical linker imidazoleacetic acid hydrochloride for 21% efficient and stable planar perovskite solar cells. Adv. Mater. **31**(39), 1902902 (2019). <https://doi.org/10.1002/adma.201902902>
4. Y. Zhou, Z. Wang, J. Jin, X. Zhang, J. Zou et al., Manipulation of the buried interface for robust formamidinium-based Sn−Pb perovskite solar cells with NiO_x_ hole-transport layers. Angew. Chem. Int. Ed. **62**(15), e202300759 (2023). <https://doi.org/10.1002/anie.202300759>
5. G. Xing, N. Mathews, S.S. Lim, N. Yantara, X. Liu et al., Low-temperature solution-processed wavelength-tunable perovskites for lasing. Nat. Mater. **13**(5), 476–480 (2014). <https://doi.org/10.1038/nmat3911>
6. L. Yin, C. Ding, C. Liu, C. Zhao, W. Zha et al., A multifunctional molecular bridging layer for high efficiency, hysteresis-free, and stable perovskite solar cells. Adv. Energy Mater. **13**(25), 2301161 (2023). <https://doi.org/10.1002/aenm.202301161>
7. B. Liu, Y. Wang, Y. Wu, Y. Zhang, J. Lyu et al., Vitamin natural molecule enabled highly efficient and stable planar n–p homojunction perovskite solar cells with efficiency exceeding 24.2%. Adv. Energy Mater. **13**(2), 2203352 (2023). <https://doi.org/10.1002/aenm.202203352>
8. Y. Qi, M. Almtiri, H. Giri, S. Jha, G. Ma et al., Evaluation of the passivation effects of PEDOT: PSS on inverted perovskite solar cells. Adv. Energy Mater. **12**(46), 2202713 (2022). <https://doi.org/10.1002/aenm.202202713>
9. T. Yang, L. Gao, J. Lu, C. Ma, Y. Du et al., One-stone-for-two-birds strategy to attain beyond 25% perovskite solar cells. Nat. Commun. **14**(1), 839 (2023). <https://doi.org/10.1038/s41467-023-36229-1>
10. J. Zhang, S. Wu, T. Liu, Z. Zhu, A.K.Y. Jen, Boosting photovoltaic performance for lead halide perovskites solar cells with BF4− anion substitutions. Adv. Funct. Mater. **29**(47), 1808833 (2019). <https://doi.org/10.1002/adfm.201808833>
11. X. Pu, Q. Cao, J. Su, J. Yang, T. Wang et al., One-step construction of a perovskite/TiO_2_ heterojunction toward highly stable inverted all-layer-inorganic CsPbI_2_Br perovskite solar cells with 17.1% efficiency. Adv. Energy Mater. **13**(36), 2301607 (2023). <https://doi.org/10.1002/aenm.202301607>
12. S. Liu, J. Li, W. Xiao, R. Chen, Z. Sun et al., Buried interface molecular hybrid for inverted perovskite solar cells. Nature **632**(8025), 536–542 (2024). <https://doi.org/10.1038/s41586-024-07723-3>
13. T.D. Kühne, M. Iannuzzi, M. Del Ben, V.V. Rybkin, P. Seewald et al., CP2K: an electronic structure and molecular dynamics software package - Quickstep: Efficient and accurate electronic structure calculations. J. Chem. Phys. **152**(19), 194103 (2020). <https://doi.org/10.1063/5.0007045>
14. T. Lu, F. Chen, Multiwfn: a multifunctional wavefunction analyzer. J. Comput. Chem. **33**(5), 580–592 (2012). <https://doi.org/10.1002/jcc.22885>
15. J. Zhang, T. Lu, Efficient evaluation of electrostatic potential with computerized optimized code. Phys. Chem. Chem. Phys. **23**(36), 20323–20328 (2021). <https://doi.org/10.1039/D1CP02805G>
16. T. Lu, Q. Chen, Interaction Region indicator: a simple real space function clearly revealing both chemical bonds and weak interactions. Chemistry–Methods **1**(5), 231–239 (2021). <https://doi.org/10.1002/cmtd.202100007>
17. W. Humphrey, A. Dalke, K. Schulten, VMD: Visual molecular dynamics. J. Mol. Graph. **14**(1), 33–38 (1996). <https://doi.org/10.1016/0263-7855(96)00018-5>
